# Use of the prevented fraction for the population to determine deaths averted by existing prevalence of physical activity: a descriptive study

**DOI:** 10.1016/S2214-109X(20)30211-4

**Published:** 2020-06-17

**Authors:** Tessa Strain, Søren Brage, Stephen J Sharp, Justin Richards, Marko Tainio, Ding Ding, Jacques Benichou, Paul Kelly

**Affiliations:** a Medical Research Council Epidemiology Unit, University of Cambridge, Cambridge, UK; b Physical Activity for Health Research Centre, Institute of Sport, Physical Education, and Health Sciences, Moray House School of Education and Sport, University of Edinburgh, Edinburgh, UK; c Prevention Research Collaboration, Sydney School of Public Health, Faculty of Medicine and Health and Charles Perkins Centre, The University of Sydney, Camperdown, NSW, Australia; d Health Services Research Centre, Faculty of Health, Victoria University of Wellington, Wellington, New Zealand; e Sustainable Urbanisation Programme, Finnish Environment Institute, Helsinki, Finland; f Systems Research Institute, Polish Academy of Sciences, Warsaw, Poland; g Department of Biostatistics and Clinical Research, Rouen University Hospital, Rouen, France; h French National Institute of Health and Medical Research, Unit 1018, University of Rouen, Rouen, France

## Abstract

**Background:**

Disease and mortality burdens of unhealthy lifestyle behaviours are often reported. In contrast, the positive narrative around the burdens that an existing behaviour have averted is rarely acknowledged. We aimed to estimate the prevented fraction for the population (PFP) for premature mortality averted by physical activity on a global scale.

**Methods:**

In this descriptive study, we obtained previously published data on physical activity prevalence (2001–16) and relative risks of all-cause mortality for 168 countries. We combined the data in Monte-Carlo simulations to estimate country-specific, mean PFP values, corresponding to percentage of mortality averted, and their 95% CIs. High prevented fractions indicated an increased proportion of deaths averted due to physical activity. Using mortality data for all people in a country aged 40–74 years, we estimated the number of premature deaths averted for all adults and by gender. We present the median and range of the prevented fractions globally, by WHO region, and by World Bank income classification.

**Findings:**

The global median PFP was 15·0% (range 6·6–20·5), conservatively equating to 3·9 million (95% CI 2·5–5·6) premature deaths averted annually. The African region had the highest median prevented fraction (16·6% [range 12·1–20·5]) and the Americas had the lowest (13·1% [10·8–16·6]). Low-income countries tended to have higher prevented fractions (group median 17·9% [12·3–20·5]) than high-income countries (14·1% [6·6–17·8]). Globally, the median prevented fraction was higher for men (16·0% [7·8–20·7] than women (14·1% [5·0–20·4]).

**Interpretation:**

Existing physical activity prevalence has contributed to averting premature mortality across all countries. PFP has utility as an advocacy tool to promote healthy lifestyle behaviours. By making the case of what has been achieved, the prevented fraction can show the value of current investment and services, which might be conducive to political support.

**Funding:**

UK Medical Research Council, British Heart Foundation, Cancer Research UK, Economic and Social Research Council, National Institute for Health Research, Wellcome Trust, Heart Foundation Australia.

## Introduction

When making the case for the promotion of healthy lifestyle behaviours, statistics that quantify the burden of reciprocal unhealthy behaviours are often cited.^1^, ^2^, ^3^ For example, physical inactivity is responsible for 6·4% of global premature mortality, insufficient fruit and vegetable consumption for 11·3%, and harmful alcohol consumption for 5·3%;^3^, ^4^, ^5^ although methodological differences can lead to substantially different results.^6^, ^7^ Some researchers advocate that stressing the harms of unhealthy behaviours can strengthen the case in promoting healthy lifestyles.^8^ We suggest that an alternative strategy is to present the complementary positive, gain-framed message that clarifies to what extent existing healthy behaviours have already reduced burdens of disease and mortality.^9^

In this Article, we describe and explore the use of the prevented fraction for the population (PFP), defined as the proportion of a disease outcome or mortality that has been averted or prevented due to the presence of a protective factor (panel).^10^ This is not a new concept: it was first proposed by Miettinen^12^ in 1974, and is included in the International Epidemiological Association's A Dictionary of Epidemiology,^10^ and the Encyclopedia of Epidemiologic Methods of the Wiley Reference Series in Biostatistics.^13^ PFP is a concept closely related to population attributable fraction (PAF), defined as the proportion of disease cases or mortality cases attributable to a harmful risk factor^10^ (in other words, the proportion that could be averted or prevented if a harmful risk factor was eliminated). The appendix (pp 1–2) presents graphical depictions of the two concepts. PAF would provide an aspirational indicator of what could be achieved if all members of the public adopted a healthy behaviour. Under most circumstances, such widespread adoption would need new investment and resources. Conversely, PFP can be used as a positive indicator of accomplishment, in terms of how much good is being done or has been done by the amount of existing beneficial behaviours within a population. As such, PFP could be a powerful advocacy tool among policy makers, by promoting positive thinking around healthy lifestyle behaviours (ie, “look how much benefit this behaviour is already providing; let's create more benefit by increasing prevalence of the behaviour further”). Although this approach has the risk of causing complacency (ie, “the behaviour is already providing benefit, why invest in more?”), it could also be a lever to protect and maintain services. As such, this could be a potentially powerful approach with realistic targets in challenging economic climates.

Panel **Prevented fraction for the population (PFP)**

PFP for a given outcome is defined by the formula:

$$\text{PFP}=\frac{\text{incidence rate in the unexposed}-\text{incidence rate in the population}}{\text{incidence rate in the unexposed}}$$

In this equation (denoted formula 1), the exposure is protective.^10^ PFP can also be expressed in terms of the prevalence of exposure (P_e_) and the relative risk (RR) for exposure relative to non-exposure^11^ (formula 2; appendix p 8):

PFP=P_e_ (1–RR)

If potential confounders are present that affect both the prevalence of exposure and the risk of disease or mortality, the following formula (denoted formula 3; appendix p 9) is recommended,^11^ where P_d_ is the prevalence of exposure among disease cases or deceased cases:

$$\text{PFP}=\frac{{\text{P}}_{\text{d}}\left(1-\text{RR}\right)}{\left[1-\left(1-\text{RR}\right)\left(1-{\text{P}}_{\text{d}}\right)\right]}$$

The proportion estimated by each of these formulae can be interpreted as the reduction in the burden of disease or mortality that has occurred due to existing exposure levels, compared with if the whole population was unexposed.

For example, in the present study of physical activity and premature mortality, we estimated the global median PFP to be 15·0% (range 6·6–20·5). This means that, due to existing physical activity levels, the burden of premature mortality is 15% lower than it would have been if the whole population was inactive.

The hypothetical denominator is analogous to a situation in which an individual buys a discounted item. The individual might pay £0·80 for an item (analogous to the observed number of deaths), and be told that they have saved £0·20 (analogous to the estimated number of cases that have been averted). The original £1·00 cost of the item was never experienced (ie, the hypothetical total number of deaths), but it is the correct denominator of the fraction to calculate the percentage saved (20%).

**Research in context**

**Evidence before this study**

The case for promoting healthy lifestyle behaviours is often framed negatively in terms of the harm (disease and death) that can be attributed to the absence of healthy behaviours or presence of unhealthy behaviours. In an initial literature search we aimed to identify examples of use of prevented fraction for the population (PFP) relating to lifestyle behaviours, such as physical activity, diet and nutrition, alcohol, and smoking. Because of the variety of terms used for the concept, we searched PubMed and WebofScience for the terms “prevented fraction”, “preventive fraction” and “preventable fraction”. We searched for papers published from database inception until June 12, 2019, without language restrictions. Compared with the thousands of studies on disease or mortality burden, we identified only 12 published studies that used a version of PFP to estimate the proportion of a disease or mortality burden that is already being averted or prevented by existing prevalence of a healthy behaviour. However, no studies had estimated prevented fraction at a global level for total physical activity behaviour, nor had the recommended formula for addressing confounding been used.

**Added value of this study**

To the best of our knowledge, this study is the first to quantify the effect that existing total physical activity prevalence has had on averting premature mortality at the global, regional, and country levels. We showed that during 2001–16 across 168 countries, the median PFP for premature mortality averted by existing physical activity prevalence was 15·0%, conservatively equating to 3.9 million deaths averted annually. We also provided information at a regional level: Africa had the highest median PFP, indicating the greatest percentage of premature mortality averted by physical activity. The Americas had the lowest prevented fraction, closely followed by the Eastern Mediterranean region. We further identified a tendency towards increased prevented fractions in lower income countries compared with higher income countries, and in men compared with women.

**Implications of all the available evidence**

PFP makes the complementary positive case for physical activity promotion, focusing on the benefits currently accomplished rather than the harms from insufficient compliance to recommended levels. This might be a powerful advocacy tool in terms of persuading policy makers and wider societies that physical activity behaviour has quantifiable value, and that existing prevalence should be at least preserved.

The few studies that have used PFP are not easy to trace because of inconsistency in terminology. The terms preventive fraction and preventable fraction are variously used. We searched the literature for the three different terms and found the concept in question has been used primarily in oral health, vaccine, and genetic research (appendix pp 3–7). We found 12 examples of use that quantified the proportion of disease burden or mortality burden that had been averted or could have been averted due to a current exposure, in relation to lifestyle behaviours or their physiological markers. Specifically, these studies quantified averted disease or mortality burden due to cycling or walking behaviour or fitness, fruit and vegetable or vitamin consumption, aspirin intake, use of oral contraceptives, sunscreen use, preventive measures for problem drinking, and interventions to prevent fractures. All of these studies determined PFP with formula 2, presented in the panel. Formula 2 is appropriate for scenarios with no confounding factors affecting the relationship between exposure prevalence and the risk of the disease outcome.^14^ As the absence of confounding is a rarity in the study of lifestyle behaviours, formula 3 (panel) is preferable, which addresses the potential sources of confounding.^11^ To the best of our knowledge, such an approach has not been applied before to global physical activity prevalence.

In this study, we present the first global application of PFP, using formula 3, to obtain estimates of the proportion and number of premature deaths averted by the existing prevalence of physical activity. We obtained results for 168 countries with two counterfactual scenarios for comparison: no activity in the population, and a plausible minimum physical activity prevalence.

## Methods

### Study design and formulae application

We did a descriptive study to estimate PFP from the existing prevalence of physical activity for all-cause mortality in 168 countries, using the formula that addresses potential confounding factors (formula 3; panel). The study protocol including all modifications is provided in the appendix (pp 10–11).

We defined physical activity as meeting the WHO global recommendation of at least 150 min of moderate-intensity aerobic activity, or 75 min of vigorous-intensity activity, or an equivalent combination, throughout one week.^15^ We used population-level, age-standardised and harmonised prevalence data on physical activity for the years 2001–16 for 168 countries, published by Guthold and colleagues,^16^ to estimate the prevalence of physical activity among people who died (P_d_), with the formula:

$${\text{P}}_{\text{d}}=\left({\text{p}}_{\text{e}}\times \text{RR}\right)/\left({\text{p}}_{\text{e}}\times \text{RR}\right)+\left(1-{\text{P}}_{\text{e}}\right)$$

In this formula (denoted formula 4; appendix p 9), P_e_ is the prevalence of activity in the population^16^ and RR is the unadjusted relative risk of premature all-cause mortality for active individuals of the population, compared with inactive individuals. The unadjusted RR was taken from a meta-analysis on the association between physical inactivity and all-cause mortality by Lee and colleagues.^17^ We estimated 95% CIs for P_d_ using the 95% CIs for P_e_ in this formula.

We used the P_d_ estimates and adjusted RR, also estimated by Lee and colleagues,^17^ in formula 3 to estimate PFP for each country. To estimate the error conservatively, we generated distributions for P_d_ and adjusted RR that reflected their 95% CIs. We used a binomial distribution for P_d_ and a normal distribution for the natural log of RR.

Subsequently, we did 10 000 Monte-Carlo simulations, in which each simulation selected one value from the generated P_d_ and RR distributions, and used them in formula 2 (panel). We considered the 10 000 estimates produced by the simulations to represent the probability distribution of the true PFP. We present the mean of these simulated values, with the 95% CIs represented by the 2·5th and 97·5th percentiles of the distribution. The appendix presents a graphical overview of the method (p 12), and the parameters for generated prevalence distributions of RR and P_d_ (pp 13–21).

### Mortality averted estimation

To convert PFP into an estimate of number of premature deaths averted, we obtained the country-specific number of all-cause deaths between the ages of 40–74 years from the mortality datasets of the UN World Population Prospects tool. We chose this age range to reflect the ages at which premature mortality could most plausibly be caused by inactivity. We selected mortality data from July 1, 2010, to June 30, 2015, which is within the period represented by prevalence data, and divided by 5 to estimate annual figures. By dividing the observed number of deaths by 1 minus the prevented fraction, we obtained estimates for the hypothetical total number of deaths that would have been observed had there been no activity in the population in each nation (ie, a zero counterfactual). The total number of premature deaths averted was the difference between the observed and hypothetical totals. A 95% CI range for the estimate of deaths averted was calculated with the lower and upper 95% CIs of the prevented fraction.

In addition to presenting the country-level estimates, we summarised the results by WHO region, and by 2019 World Bank income classification;^18^ each of these groupings were further subdivided into gender-specific results according to the UN mortality datasets. Countries without an income categorisation (Cook Islands, Niue, occupied Palestinian territory, and Tokelau) were classified according to similar neighbouring economies. As these groups of countries were small (often <20) and the estimates did not typically follow a normal distribution, we present group-level medians and ranges to summarise the data; summary values representative of all 168 countries are presented in the same way for comparability.

For 14 nations we were unable to obtain mortality data; these made up less than 0·01% of the world's population (appendix p 22). For these countries, we imputed mortality data at ages 40–74 years on the basis of the median ratio of deaths to the total population in countries in the same WHO region and World Bank income categories; these median ratios were multiplied by the population of the country without mortality data.

### Sensitivity analysis

We did three sensitivity analyses. The first two used formula 2: with the unadjusted RR to provide unadjusted results, and with the adjusted RR as this was the method most commonly used in the literature to date (appendix pp 4–7). In the third analysis we used a plausible minimum physical activity prevalence counterfactual. This comparison attempted to focus on the burden averted due to modifiable physical activity prevalence, not including the residual levels (ie, individuals who would be active regardless of initiatives to promote activity). For this comparison, we used the median of the lowest observed prevalence of activity from each region. We opted for this measure rather than the observed lowest prevalence from a single nation because environmental and cultural factors vary widely between regions and might influence activity levels. Using formula 4, we obtained a P_d_ for this minimum prevalence of activity. We adapted formula 3 to allow for a non-zero counterfactual, analogous to the population impact fraction formulae often used for PAF (denoted formula 5; appendix pp 23–25).^11^ Countries with a prevalence of activity lower than the chosen counterfactual value were excluded from this analysis. All analyses were done in STATA (version 15.1), and forest plots of the data were produced in RStudio (version 1.2.5033).

### Role of the funding source

The funders of the study had no role in study design, data collection, data analysis, data interpretation, or writing of the report. The corresponding author had full access to all the data in the study and had final responsibility for the decision to submit for publication.

## Results

For the 168 nations included in this study, the global median PFP (determined with formula 3) was 15·0% (range 6·6–20·5), equating to 3·9 million (95% CI 2·5–5·6) premature deaths averted per year (figure, table).

**Figure:**
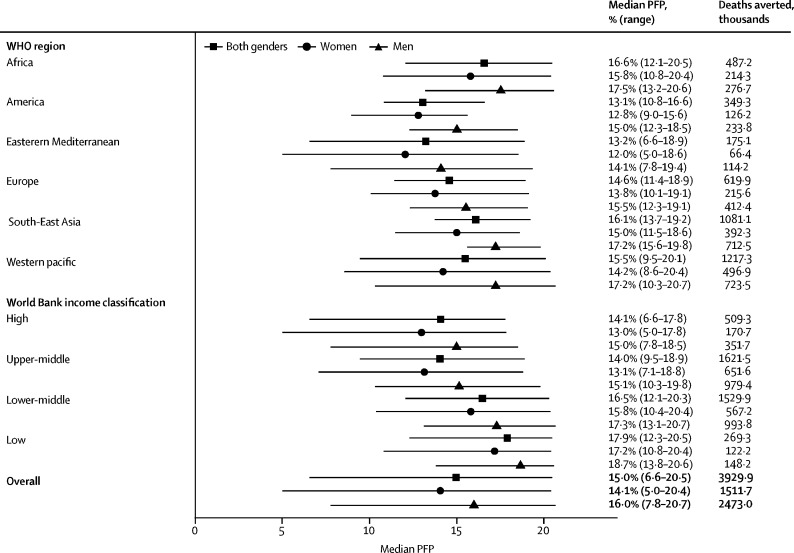

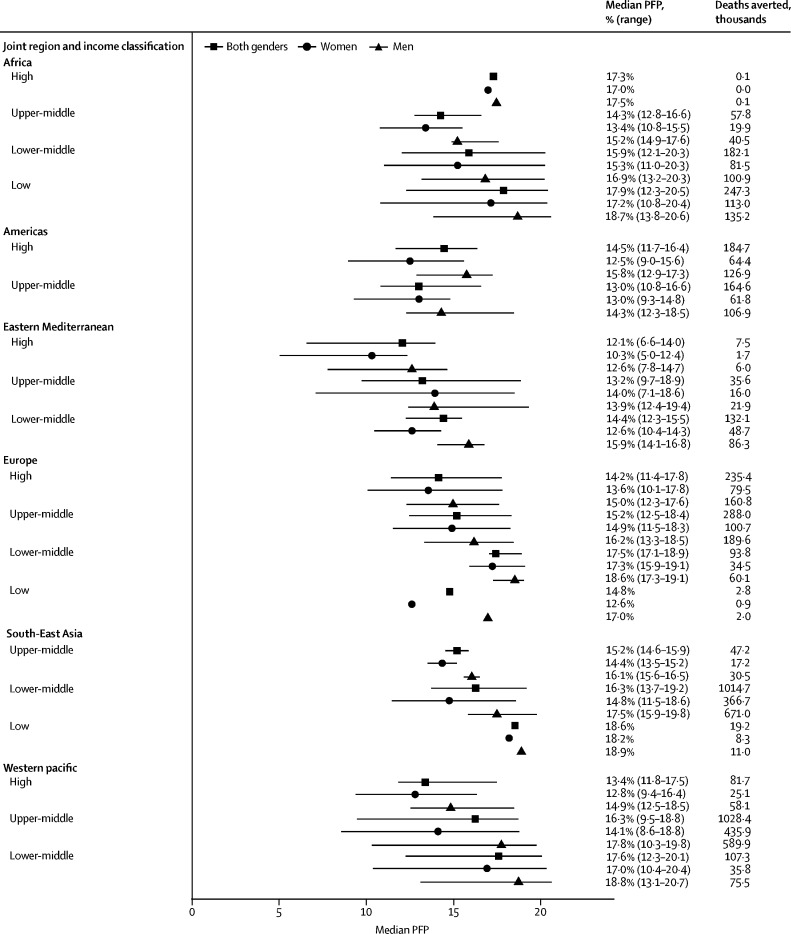
Median PFP estimates and corresponding number of deaths averted No range is presented for region and income groups with only one country. Countries in each region and income group are shown in the appendix (pp 29–36). Deaths averted is for the age range 40–74 years. Number of deaths averted for men and women might not add up to the estimate for both genders because the distributions of deaths across men and women are not even, and differences are magnified when multiplied by a derivative of activity prevalence, which also varies considerably by gender. PFP=prevented fraction for the population.

**Table:** PFP estimates and corresponding number of deaths averted in main and sensitivity analyses

			**PFP, %**												**Deaths averted between ages 40–74 years, thousands (Main analysis)**		
			Main adjusted estimate (0% activity counterfactual)*			Unadjusted estimate (0% activity counterfactual)†			Partially adjusted estimate (0% activity counterfactual)‡			Adjusted estimate (54·3% active counterfactual)§					
			Both genders	Women	Men	Both genders	Women	Men	Both genders	Women	Men	Both genders	Women	Men	Both genders	Women	Men
Overall			15·0% (6·6–20·5)	14·1% (5·0–20·4)	16·0% (7·8–20·7)	22·9% (10·5–30·2)	21·6% (8·1–30·1)	24·2% (12·4–30·5)	15·6% (7·2–20·6)	14·7% (5·5–20·6)	16·6% (8·4–20·8)	4·5% (0·3–10·5)	5·3% (0·2–12·1)	4·3% (0·0–9·6)	3929·9 (2480·3–5602·4)	1511·7 (934·5–2188·9)	2473·0 (1583·6–3488·0)
WHO region																	
	Africa		16·6% (12·1–20·5)	15·8% (10·8–20·4)	17·5% (13·2–20·6)	25·0% (18·8–30·2)	24·0% (16·8–30·1)	26·4% (20·3–30·4)	17·1% (12·8–20·6)	16·4% (11·5–20·6)	18·0% (13·8–20·7)	6·1% (1·1–10·5)	6·9% (1·4–12·1)	6·0% (1·0–9·4)	487·2 (315·1–680·7)	214·3 (138·2–300·4)	276·7 (180·6–385·0)
	Americas		13·1% (10·8–16·6)	12·8% (9·0–15·6)	15·0% (12·3–18·5)	20·1% (16·9–25·1)	19·7% (14·2–23·7)	22·9% (19·0–27·7)	13·8% (11·6–17·1)	13·5% (9·7–16·2)	15·6% (13·0–18·9)	3·3% (0·3–6·2)	4·0% (0·2–6·8)	3·2% (0·4–7·1)	349·3 (221·9–498·2)	126·2 (79·1–182·6)	233·8 (150·2–329·8)
	Eastern Mediterranean		13·2% (6·6–18·9)	12·0% (5·0–18·6)	14·1% (7·8–19·4)	20·4% (10·5–28·2)	18·7% (8·1–27·7)	21·6% (12·4–28·7)	13·9% (7·2–19·2)	12·8% (5·5–18·8)	14·7% (8·4–19·6)	3·1% (1·1–8·8)	3·3% (0·9–10·0)	2·3% (0·1–8·0)	175·1 (106·9–256·7)	66·4 (39·0–99·8)	114·2 (72·1–163·1)
	Europe		14·6% (11·4–18·9)	13·8% (10·1–19·1)	15·5% (12·3–19·1)	22·3% (17·8–28·3)	21·1% (15·8–28·5)	23·6% (19·1–28·5)	15·2% (12·1–19·3)	14·4% (10·8–19·5)	16·1% (13·1–19·4)	3·9% (0·3–8·9)	4·7% (0·6–10·7)	3·6% (0·0–7·8)	619·9 (399·4–873·5)	215·6 (137·8–304·6)	412·4 (266·8–576·7)
	South-East Asia		16·1% (13·7–19·2)	15·0% (11·5–18·6)	17·2% (15·6–19·8)	24·4% (21·2–28·6)	22·9% (17·9–27·8)	25·9% (23·7–29·4)	16·7% (14·4–19·5)	15·6% (12·3–18·9)	17·7% (16·2–20·1)	5·6% (2·9–9·1)	6·1% (2·2–10·1)	5·6% (3·8–8·6)	1081·1 (655·8–1586·2)	392·3 (223·4–597·3)	712·5 (446·8–1020·6)
	Western Pacific		15·5% (9·5–20·1)	14·2% (8·6–20·4)	17·2% (10·3–20·7)	23·6% (14·9–29·8)	21·8% (13·6–30·1)	25·9% (16·2–30·5)	16·1% (10·2–20·3)	14·9% (9·2–20·5)	17·7% (11·1–20·8)	5·2% (0·5–10·1)	5·9% (0·7–12·0)	5·9% (0·3–9·6)	1217·3 (781·2–1707·2)	496·9 (316·9–704·2)	723·5 (467·1–1012·8)
World Bank income group classification																	
	High		14·1% (6·6–17·8)	13·0% (5·0–17·8)	15·0% (7·8–18·5)	21·6% (10·5–26·7)	20·1% (8·1–26·9)	22·9% (12·4–27·7)	14·7% (7·2–18·2)	13·8% (5·5–18·4)	15·7% (8·4–18·9)	3·4% (0·3–7·5)	4·1% (0·6–9·3)	3·1% (0·0–7·1)	509·3 (319·6–733·0)	170·7 (105·5–248·5)	351·7 (222·8–500·9)
	Upper-middle		14·0% (9·5–18·9)	13·1% (7·1–18·8)	15·1% (10·3–19·8)	21·5% (14·9–28·2)	20·3% (11·3–28·1)	23·1% (16·2–29·4)	14·7% (10·2–19·2)	13·8% (7·7–19·1)	15·8% (11·1–20·0)	3·8% (0·3–8·8)	4·4% (0·2–10·3)	3·5% (0·1–8·6)	1621·5 (1046·2–2269·0)	651·6 (417·7–920·0)	979·4 (635·8–1365·6)
	Lower-middle		16·5% (12·1–20·3)	15·8% (10·4–20·4)	17·3% (13·1–20·7)	24·9% (18·8–29·9)	24·0% (16·3–30·1)	26·0% (20·2–30·5)	17·0% (12·8–20·5)	16·4% (11·1–20·5)	17·8% (13·8–20·8)	6·0% (1·1–10·3)	7·0% (0·9–12·0)	5·7% (0·9–9·6)	1529·9 (938·8–2226·4)	567·2 (331·7–850·7)	993·8 (627·9–1416·6)
	Low		17·9% (12·3–20·5)	17·2% (10·8–20·4)	18·7% (13·8–20·6)	26·9% (19·1–30·2)	25·9% (16·9–30·1)	27·8% (21·2–30·4)	18·3% (13·0–20·6)	17·7% (11·5–20·6)	19·0% (14·5–20·7)	7·7% (1·3–10·5)	8·5% (1·4–12·1)	7·3% (1·7–9·4)	269·3 (175·7–374·0)	122·2 (79·5–169·7)	148·2 (97·1–204·9)
WHO region and World Bank income group classification																	
	Africa																
		High	17·3%	17·0%	17·5%	26·0%	25·6%	26·4%	17·7%	17·5%	17·9%	6·9%	8·3%	5·9%	0·1 (0·1–0·1)	0·0 (0·0–0·0)	0·1 (0·0–0·1)
		Upper-middle	14·3% (12·8–16·6)	13·4% (10·8–15·5)	15·2% (14·9–17·6)	21·9% (19·8–25·0)	20·7% (16·8–23·6)	23·2% (22·7–26·5)	14·9% (13·5–17·0)	14·1% (11·5–16·1)	15·9% (15·5–18·2)	3·6% (1·9–6·2)	4·3% (1·4–6·6)	3·4% (3·0–6·1)	57·8 (36·4–82·9)	19·912·3–29·2)	40·5 (26·2–57·0)
		Lower-middle	15·9% (12·1–20·3)	15·3% (11·0–20·3)	16·9% (13·2–20·3)	24·1% (18·8–29·9)	23·2% (17·2–30·0)	25·4% (20·3–29·9)	16·5% (12·8–20·5)	15·9% (11·7–20·5)	17·4% (13·8–20·4)	5·4% (1·1–10·3)	6·4% (1·7–12·0)	5·2% (1·0–9·1)	182·1 (117·3–254·3)	81·5 (52·3–114·4)	100·9 (65·7–141·0)
		Low	17·9% (12·3–20·5)	17·2% (10·8–20·4)	18·7% (13·8–20·6)	26·9% (19·1–30·2)	25·9% (16·9–30·1)	27·8% (21·2–30·4)	18·3% (13·0–20·6)	17·7% (11·5–20·6)	19·0% (14·5–20·7)	7·7% (1·3–10·5)	8·5% (1·4–12·1)	7·3% (1·7–9·4)	247·3 (161·3–343·4)	113·0 (73·6–156·9)	135·2 (88·6–186·9)
	Americas																
		High	14·5% (11·7–16·4)	12·5% (9·0–15·6)	15·8% (12·9–17·3)	22·2% (18·1–24·8)	19·4% (14·2–23·7)	24·0% (20·0–26·0)	15·1% (12·4–17·0)	13·2% (9·7–16·2)	16·4% (13·6–17·7)	3·8% (0·6–6·0)	3·9% (1·1–6·8)	4·0% (0·7–5·7)	184·7 (117·9–262·9)	64·4 (40·4–93·2)	126·9 (81·9–178·4)
		Upper-middle	13·0% (10·8–16·6)	13·0% (9·3–14·8)	14·3% (12·3–18·5)	20·1% (16·9–25·1)	20·1% (14·6–22·6)	22·0% (19·0–27·7)	13·7% (11·6–17·1)	13·7% (10·0–15·4)	15·0% (13·0–18·9)	2·2% (0·3–6·2)	4·0% (0·2–5·8)	2·4% (0·4–7·1)	164·6 (104·0–235·4)	61·8 (38·7–89·4)	106·9 (68·3–151·4)
	Eastern Mediterranean																
		High	12·1% (6·6–14·0)	10·3% (5·0–12·4)	12·6% (7·8–14·7)	18·7% (10·5–21·5)	16·2% (8·1–19·1)	19·5% (12·4–22·4)	12·8% (7·2–14·6)	11·0% (5·5–13·0)	13·3% (8·4–15·3)	2·2% (1·1–3·2)	1·0% (0·9–3·1)	1·8% (0·3–2·7)	7·5 (4·6–10·9)	1·7 (1·0–2·7)	6·0 (3·7–8·6)
		Upper-middle	13·2% (9·7–18·9)	14·0% (7·1–18·6)	13·9% (12·4–19·4)	20·4% (15·3–28·2)	21·4% (11·3–27·7)	21·3% (19·3–28·7)	13·9% (10·5–19·2)	14·6% (7·7–18·8)	14·6% (13·1–19·6)	2·7% (2·3–8·8)	6·2% (2·8–10·0)	1·8% (0·1–8·0)	35·6 (22·5–50·9)	16·0 (10·3–22·6)	21·9 (13·9–31·1)
		Lower-middle	14·4% (12·3–15·5)	12·6% (10·4–14·3)	15·9% (14·1–16·8)	22·1% (19·0–23·6)	19·6% (16·4–21·9)	24·1% (21·6–25·4)	15·0% (13·0–16·1)	13·4% (11·2–14·9)	16·5% (14·7–17·3)	3·7% (1·3–5·0)	3·5% (1·0–5·3)	4·1% (2·0–5·1)	132·1 (79·8–194·8)	48·7 (27·7–74·5)	86·3 (54·5–123·3)
	Europe																
		High	14·2% (11·4–17·8)	13·6% (10·1–17·8)	15·0% (12·3–17·6)	21·7% (17·8–26·7)	20·8% (15·8–26·9)	22·9% (19·1–26·5)	14·8% (12·1–18·2)	14·3% (10·8–18·4)	15·7% (13·1–18·0)	3·4% (0·3–7·5)	4·5% (0·6–9·3)	3·1% (0·0–6·1)	235·4 (150·7–333·3)	79·5 (50·5–113·0)	160·8 (103·4–225·9)
		Upper-middle	15·2% (12·5–18·4)	14·9% (11·5–18·3)	16·2% (13·3–18·5)	23·2% (19·3–27·5)	22·8% (18·0–27·3)	24·5% (20·6–27·6)	15·8% (13·3–18·7)	15·6% (12·3–18·7)	16·7% (14·1–18·8)	4·6% (1·5–8·2)	6·0% (2·2–9·7)	4·4% (1·2–7·0)	288·0 (186·2–404·8)	100·7 (64·5–141·6)	189·6 (123·0–263·9)
		Lower-middle	17·5% (17·1–18·9)	17·3% (15·9–19·1)	18·6% (17·3–19·1)	26·2% (25·7–28·3)	26·0% (24·2–28·5)	27·7% (26·0–28·5)	17·9% (17·6–19·3)	17·8% (16·5–19·5)	18·9% (17·7–19·4)	7·1% (6·7–8·9)	8·6% (7·1–10·7)	7·1% (5·7–7·8)	93·8 (60·7–131·3)	34·5 (22·2–48·7)	60·1 (39·1–84·1)
		Low	14·8%	12·6%	17·0%	22·6%	19·6%	25·6%	15·4%	13·4%	17·5%	4·1%	3·4%	5·3%	2·8 (1·8–4·0)	0·9 (0·6–1·3)	2·0 (1·3–2·8)
	South-East Asia																
		Upper-middle	15·2% (14·6–15·9)	14·4% (13·5–15·2)	16·1% (15·6–16·5)	23·2% (22·3–24·1)	22·1% (20·9–23·3)	24·3% (23·7–25·0)	15·8% (15·2–16·5)	15·1% (14·2–15·9)	16·6% (16·2–17·1)	4·6% (3·9–5·4)	5·4% (4·4–6·3)	4·3% (3·8–4·8)	47·2 (30·4–66·2)	17·2 (11·0–24·6)	30·5 (19·9–43·0)
		Lower-middle	16·3% (13·7–19·2)	14·8% (11·5–18·6)	17·5% (15·9–19·8)	24·6% (21·2–28·6)	22·5% (17·9–27·8)	26·3% (24·1–29·4)	16·8% (14·4–19·5)	15·4% (12·3–18·9)	17·9% (16·5–20·1)	5·8% (2·9–9·1)	5·8% (2·2–10·1)	6·0% (4·1–8·6)	1014·7 (612·9–1493·3)	366·7 (207·0–561·2)	671·0 (419·7–962·5)
		Low	18·6%	18·2%	18·9%	27·7%	27·3%	28·1%	18·9%	18·6%	19·2%	8·4%	9·7%	7·5%	19·2 (12·6–26·6)	8·3 (5·4–11·5)	11·0 (7·2–15·2)
	Western Pacific																
		High	13·4% (11·8–17·5)	12·8% (9·4–16·4)	14·9% (12·5–18·5)	20·7% (18·4–26·3)	19·9% (14·8–24·7)	22·7% (19·4–27·7)	14·1% (12·5–17·9)	13·5% (10·1–16·9)	15·5% (13·2–18·9)	2·6% (0·8–7·2)	3·9% (1·9–7·6)	2·9% (0·3–7·1)	81·7 (46·3–125·8)	25·1 (13·6–39·7)	58·1 (33·7–87·9)
		Upper-middle	16·3% (9·5–18·8)	14·1% (8·6–18·8)	17·8% (10·3–19·8)	24·6% (14·9–28·0)	21·7% (13·6–28·1)	26·6% (16·2–29·4)	16·8% (10·2–19·1)	14·8% (9·2–19·1)	18·2% (11·1–20·0)	7·0% (0·5–8·6)	6·4% (0·7–10·3)	6·4% (0·8–8·6)	1028·4 (666·7–1428·8)	435·9 (280·9–612·7)	589·9 (384·6–819·3)
		Lower-middle	17·6% (12·3–20·1)	17·0% (10·4–20·4)	18·8% (13·1–20·7)	26·5% (19·0–29·8)	25·6% (16·3–30·1)	27·9% (20·2–30·5)	18·1% (13·0–20·3)	17·4% (11·1–20·5)	19·1% (13·8–20·8)	7·3% (1·3–10·1)	8·2% (0·9–12·0)	7·4% (0·9–9·6)	107·3 (68·2–152·6)	35·8 (22·5–51·9)	75·5 (48·8–105·6)

**Data** are median (range) or number (95% CIs). No range is presented for combined region and income groups with only one country. Countries in each region and income group are shown in the appendix (pp 29–36). Number of deaths averted for men and women might not add up to the estimate for both genders because the distributions of deaths across men and women are not even, and differences are magnified when multiplied by a derivative of activity prevalence, which also varies considerably by gender. PFP=prevented fraction for the population. RR=relative risk.

* The main estimates calculated with formula 3 (panel), the adjusted RR, and 0% activity counterfactual.

† The unadjusted estimates calculated with formula 2 (panel), the unadjusted RR, and 0% activity counterfactual.

‡ The partially adjusted estimates calculated using formula 2 (panel), the adjusted RR, and 0% activity counterfactual.

§ The adjusted estimates calculated with formula 5 (appendix pp 23–25), the adjusted RR, and 54·3% active counterfactual; countries with a negative PFP (activity prevalence <54.3%) were excluded.

The WHO region with the highest PFP was Africa (16·6% [12·1–20·5]; equating to 0·5 million deaths (95% CI 0·3–0·7) averted annually) and the region with the lowest prevented fraction was the Americas (13·1% [10·8–16·6]; 0·3 million deaths (95% CI 0·2–0·5) averted annually) closely followed by the Eastern Mediterranean region (13·2% [6·6–18·9]; 0·2 million deaths (95% CI 0·1–0·3); figure, table). The countries with the highest prevented fractions were Mozambique and Uganda (both 20·5% [95% CI 14·4–26·2]) and the country with the lowest prevented fraction was Kuwait (6·5% [4·0–9·6]; appendix pp 26–36)**.** Similar results were observed for country-level data classified by gender, with women in Niue and men in Tokelau also ranking among the populations with the highest prevented fractions (appendix pp 37–51). The median prevented fraction in countries classified as low income was higher than that in lower-middle-income countries (17·9% [range 12·3–20·5] *vs* 16·5% [12·1–20·3]; figure, table). In turn, lower-middle-income countries showed a higher prevented fraction than countries classified as upper-middle income (14·0% [9·5–18·9]) or high income (14·1% [6·6–17·8]). We identified a broad pattern of higher median prevented fractions for countries of lower income classifications within most regions, but the ranges overlapped.

The global median PFP was 14·1% (5·0–20·4) for women and 16·0% (7·8–20·7) for men (figure, table, appendix pp 37–51). The regional median prevented fraction values were between 1·7 and 3·0 percentage points lower for women, with the greatest difference in the Western Pacific region. The median prevented fraction values based on World Bank income classification were between 1·5 and 2·0 percentage points lower for women, with slightly greater differences between men and women in higher income countries than in lower income countries. We estimated that 1·5 million (95% CI 0·9–2·2) annual deaths had been averted for women and 2·5 million (1·6–3·5) annual deaths for men by existing physical activity prevalence across all countries.

Sensitivity analyses with formula 2 produced higher estimates of the prevented fractions than our main analysis with formula 3. The global median prevented fraction with an unadjusted RR was 22·9% (range 10·5–30·2) and with an adjusted RR was 15·6% (7·2–20·6; table). In our third sensitivity analysis, we used a counterfactual of the median lowest observed prevalence of activity for each region, as opposed to 0% of the population meeting the physical activity guidelines (formula 5). The median lowest prevalence was 54·3%, which corresponded to a P_d_ of 44·7%. Countries that had a prevalence of activity lower than this counterfactual value were excluded (six countries overall; on gender-specific analyses, nine for women and four for men). In this analysis, global median prevented fraction was 4·5% (0·3–10·5%), which was 11·5 percentage points lower than our previous estimate (table). In all sensitivity analyses, the same patterns according to region, income classification, and gender were evident.

## Discussion

Across 168 countries, the median percentage of premature mortality averted was 15·0%, conservatively equating to 3·9 million deaths per year. We identified variations by WHO region and World Bank income classification: Africa had the highest median PFP, while the Americas and Eastern Mediterranean regions had the lowest. Countries with low-income classification tended to have higher prevented fractions than high-income countries. The prevented fractions were higher for men than for women, with similar patterns across regions and income classifications. To the best of our knowledge, these are the first national and global estimates of PFP associated with total physical activity. Furthermore, they are the first estimates derived from a formula that minimises bias from potential confounding in the disease–exposure relationship, and from the unequal distribution of activity across potential confounders. Use of PFP in this way has the potential to create a positive discourse around the existing efforts to promote physical activity.

Global differences in the prevented fractions reflect the differential prevalence of physical activity; estimations of averted deaths also factor in population size and mortality rates among people aged 40–75 years. The policy implications of the differences in activity prevalence have been discussed elsewhere.^16^ We view the implications of the present study to be similar across all nations and regions: a positive case can be made about existing amounts of physical activity, and we believe the results and the approach in general are powerful advocacy tools for policy makers and other stakeholders in public health. Use of PFP could support a positive position on physical activity that might be more conducive to political support than criticism of current policy, by showing the value of existing investment and services, particularly during economic hardship. We encourage the incorporation of these positive messages into the discourse and advocacy around physical activity policy at the global, regional, and country levels; for example, via scenario modelling to support the WHO Global Action Plan on Physical Activity^1^ and the contribution of physical activity to the attainment of the Sustainable Development Goals.^19^

PFP is a complementary statistic to PAF, with each supporting a different approach to framing public health advocacy and messaging. PFP compares the current situation with the worst-case scenario, whereas PAF compares against the best-case scenario. Therefore, if activity prevalence increases, we would expect PFP to increase (representing increased benefits being accrued), and PAF to decrease (representing decreased potential for further gains). However, they are not directly comparable as proportions because they have different denominators: the number of deaths in a hypothetical worst-case scenario (PFP), and the number of deaths in the current situation (PAF). With that in mind, we compare our PFP estimate of 15·0% for physical activity with a PAF for physical inactivity, derived with similar methods by Ding and colleagues,^4^ of 6·4%. These statistics indicate that, although mortality burden could be reduced by 6·4% by increases in activity, if the prevalence of activity were to decrease to zero, mortality would be 15·0% higher.

Despite PAF being a standard epidemiological approach, one caveat, which also applies to PFP, is that the counterfactual is the most extreme scenario. Some researchers might argue that 0% or 100% of a population meeting the global physical activity recommendations is implausible; others might debate whether a minimum physical activity prevalence exists.^20^ We therefore also estimated PFP using a minimum plausible activity prevalence of 54·3% as a counterfactual. The global median prevented fraction was 11·5 percentage points lower than when a 0% activity prevalence counterfactual was used (4·5% *vs* 15·0%). Levine^21^ argued that PAF estimates are only meaningful if an intervention can be identified that could cause the assumed reduction in disease or mortality. The equivalent for PFP would be identifying the residual amount of activity that exists regardless of intervention.^22^ However, the burden that governments or individual initiatives claim to prevent becomes the quantity that they are responsible for maintaining, and so we believe these statistics have merit as an advocacy tool to start that discussion. Regarding the interpretation of these statistics, two further points should be considered. Firstly, changes in activity prevalence are unlikely to occur independent of other risk factors such as obesity, which causes difficulty in attributing any burden of mortality entirely to one risk factor.^23^ Secondly, reductions in premature mortality, despite being a good health indicator, do not necessarily translate to an extended healthy lifespan or a reduced overall burden on the health system.^14^, ^24^

Assumptions to note in the present study include our treatment of physical activity as a binary exposure, implying no benefits of undertaking a level of activity that is insufficient to meet the global recommendation. We know this not to be the case: the greatest reduction in mortality risk is evident with initial increases in activity at the low end of the activity level spectrum, and further benefits are associated with doing more than the minimum recommended amount.^25^, ^26^ Therefore, we expect our estimates to be conservative. Our method is in line with previous work on the global burden of physical activity that used PAF estimates,^4^, ^17^, ^27^ but differs from the approach of Global Burden of Disease (GBD) studies, which used a multilevel categorisation of physical activity and the equivalent of our formula 2.^6^, ^7^ These factors, along with the choices of prevalence and mortality data, and of RR values, make a considerable difference to estimates of PAF.^6^, ^7^ For example, estimates from two different approaches gave a difference of 2 million deaths attributable to physical activity.^6^, ^7^ We expect similar variation with regard to PFP and estimated number of deaths averted. A 2020 study suggests that the magnitude of RR values for mortality associated with inactivity are increased when derived from accelerometer-measured behaviours.^28^ When global activity prevalence estimates are derived from standardised accelerometry protocols, the overall effect on estimates of PFP will be interesting to reassess.

We chose to be conservative by limiting data to deaths occurring between age 40–74 years. This age range matches that of many studies used to derive RR, and avoids counting causes of death that are not directly attributable to physical activity, such as neonatal complications or deaths among the oldest age groups that stretch the definition of avertable. We considered estimating the number of averted deaths in populations with non-communicable disease, but the available data were not provided for each country by age group. This avenue should be considered by future work if the data become available, although the events around coronavirus disease 2019 have brought into focus the role that physical activity might have in promoting immune function and preventing deaths from communicable disease.^29^ The number of assumptions required, and the variability in estimates derived from different data sources led Lee and colleagues^6^ to question whether such differences make estimates of disease or mortality burden worthless. They concluded that their provision of a reference point still had substantial utility. We concur that these statistics are helpful indications of prevented, preventable, and attributable disease and mortality burdens; a way of translating the results of epidemiological or intervention studies into public health policy and practice. Some situations such as cross-country comparisons will favour use of the prevented fraction, while others such as within-country advocacy might suit the use of the absolute number of deaths averted.

In conclusion, we estimated the median global burden of premature mortality averted by physical activity to be 15·0%, conservatively equating to 3·9 million deaths annually. We propose use of PFP as a complimentary statistic to PAF in promoting healthy lifestyle behaviours, to make the cases of both what has been and of what could be achieved.

## Data sharing

All data used in this study are publicly available and are referenced in the manuscript. The STATA code used to run the analyses is available on request.
