# Supplementary material for: Use of the prevented fraction for the population to determine deaths averted by existing prevalence of physical activity: a descriptive study
Source: Lancet Glob Health. 2020 Jun 17;8(7):e920–30. doi: 10.1016/S2214-109X(20)30211-4 (PMC7303949; doi:10.1016/S2214-109X(20)30211-4)
Supplement: Supplementary appendix [file mmc1.pdf]

THE LANCET
Global Health

Supplementary appendix

This appendix formed part of the original submission and has been peer reviewed. We post it as supplied by the authors.

Supplement to: Strain T, Brage S, Sharp SJ, et al. Use of the prevented fraction for the population to determine deaths averted by existing prevalence of physical activity: a descriptive study. *Lancet Glob Health* 2020; **8:** e920–30.

Appendix 1. Graphical depictions of the Population Attributable Fraction and the Prevented Fraction for the Population using an example of physical activity.

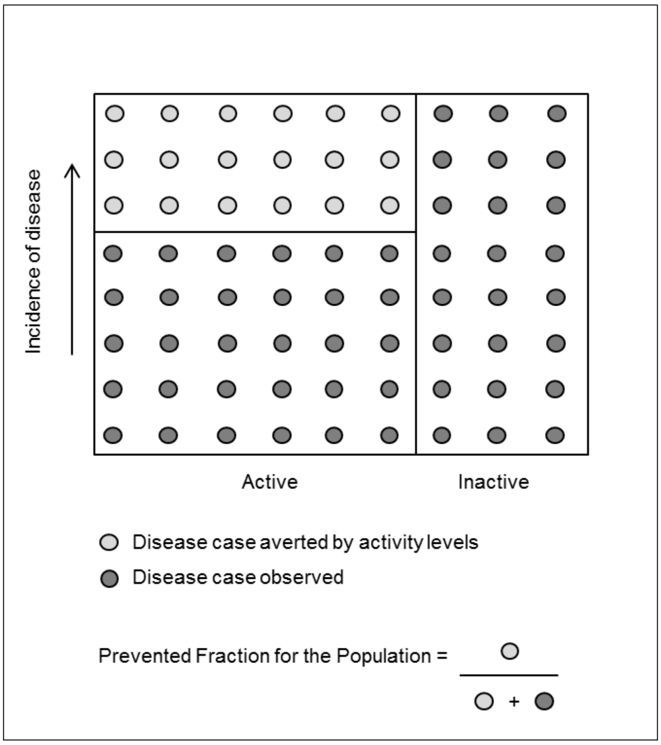

Figure 1A. The Prevented Fraction for the Population.

The observed disease cases (the dark grey circles) occur in both the active and the inactive, but the incidence is higher amongst the inactive. The number of cases that are averted by activity levels (the light grey circles) will be determined by the difference in incidence rates and by the prevalence of activity. The sum of the dark and light grey circles represents the hypothetical total number of cases that would be expected if the whole population was inactive. The Prevented Fraction for the Population expresses the number of averted cases (the light grey circles) as a proportion of the hypothetical total number of cases if the whole population were inactive (light + dark grey circles).

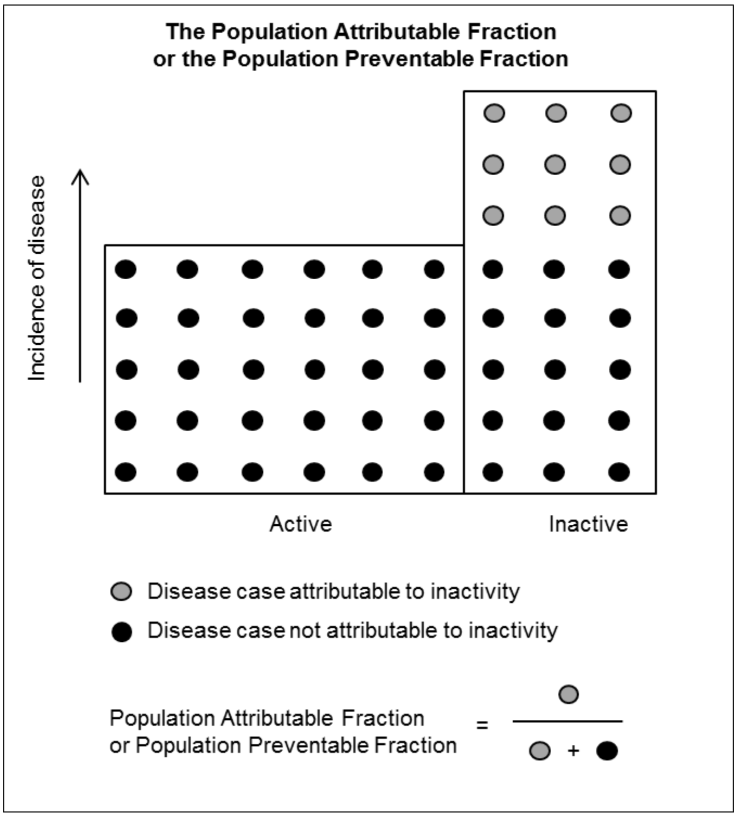

Figure 1B. The Population Attributable Fraction (also known as the Population Preventable Fraction) All circles in this figure represent observed disease cases. The black circles represent cases that occur in both the active and inactive (a 'baseline' level of disease incidence). The dark grey circles represent cases that occur in the inactive group, above this 'baseline' level. As such, they are considered attributable to inactivity levels. The Population Attributable Fraction and the Population Preventable Fraction express the attributable disease cases (dark grey circles) as a proportion of all observed disease cases (dark grey + black circles), i.e. the potential proportion of cases that could be eliminated if everyone was active.

Figures 1A and 1B adapted from Koepsell, Zatsick, & Rivara (2011) and Spasoff (1999).

Koepsell TD, Zatzick DF, Rivara FP. Estimating the Population Impact of Preventive Interventions from Randomized Trials. Am J Prev Med 2011; 40: 191-8.

Spasoff RA. Epidemiologic Methods for Health Policy. New York: Oxford University Press; 1999.

Appendix 2. A literature search to identify examples of the Prevented Fraction for the Population relating to lifestyle behaviours.

Our aim was to identify examples of use of the Prevented Fraction for the Population relating to lifestyle behaviours, such as physical activity, diet and nutrition, alcohol, and smoking. Because of the variety of terms used for the concept, we searched PubMed and WebofScience search engines for the terms “prevented fraction”, “preventive fraction” and “preventable fraction”. We performed this search on the 12th March 2019 and updated it on 12th June 2019 with no language restrictions.

We obtained the following number of hits

	PubMed	Web of Knowledge	Excluding duplicates
Prevented Fraction	125	98	134
Preventive Fraction	72	54	77
Preventable Fraction	81	63	92

We categorised the studies based on titles as follows:

	Prevented Fraction	Preventive Fraction	Preventable Fraction
Lifestyle behaviours	8	9	12
Statistical methods	15	0	8
Oral health	64	15	1
Vaccination (animal and human)	27	11	24
Genetic (mainly but not exclusively relating to human leukocyte antigen)	4	23	3
Cancer screening or surgery	7	0	1
Cancer descriptive epidemiology	2	1	1
HIV, sexual health and pregnancy	4	5	4
Suicide and mental health	2	3	3
Other non-behavioural	1	10	25
No title	0	0	10

We read the full texts of all those identified in the ‘lifestyle behaviours’ category to ascertain whether they used the Prevented Fraction for the Population as defined by the International Dictionary of Epidemiology and described in our paper. i.e. they were estimating the proportion of a disease burden that had been averted due to current levels of a risk factor, or that could have been averted due to a given level of a risk factor. These 10 papers are highlighted in grey in the table below along with two further articles, identified through further reading that met these criteria. The other papers generally estimated the proportion of a disease burden that could be averted due to future changes in a risk factor.

**Article**	**Identified under Search Term**	**Description of use**
Cuipers et al. (2004)<br>Addiction<br>99(7):839-845	Prevented Fraction	They estimated the proportion of deaths prevented by interventions for problem-drinking.
Iversen et al. (2018)<br>BMJ 362:k3609	Prevented Fraction	They estimated the proportion of ovarian cancers prevented by hormonal contraception use.
Jordan et al. (2015)<br>Aus N Z Public Health<br>39(5):441-445	Prevented Fraction	They estimated the proportion of endometrial and ovarian cancers prevented through use of the combined oral contraceptive pill.
Jordan et al. (2015)<br>Aus N Z Public Health<br>39(5):418-421	Prevented Fraction	They estimated the proportion of breast cancers that could be prevented if breast feeding prevalence and durations were increased.
McIsaac et al. (2015)<br>Can J Public Health<br>106(4):E217-E222	Prevented Fraction	They estimated the proportion of sudden infant death and other diseases that could be prevented by breast feeding.
Olson et al. (2015)<br>Aus N Z Public Health<br>39(5):471-476	Prevented Fraction	They estimated the proportion of squamous cell carcinoma and melanomas prevented by sun screen use.
Poulstrup et al. (2000)<br>Eur J Pub Health<br>10(1):45-50	Prevented Fraction	They estimated the proportion of fractures prevented during an 18-month intervention compared to a control group.
Wilson et al. (2015)<br>Aus N Z J Public Health<br>39(5):414-417	Prevented Fraction	They estimated the proportion of colorectal and oesophageal cancers prevented by daily aspirin use.
Caru et al. 2018<br>World J Cardiol<br>10(4):26-34	Preventive Fraction	They estimated the proportion of various health outcomes prevented by different levels of physical fitness amongst cardiac patients.
Carnethon et al. 2010<br>Hypertension<br>56(1):49-55	Preventive Fraction	They estimated the proportion of hypertension cases that could be prevented if participants increased their fitness.
Iversen et al. 2017<br>Am J Obstet Gynecol<br>216(6):580	Preventive Fraction	They estimated the percentage of cancer reduction that could be prevented by combined oral contraception use.

Lemay et al. 2019<br>J Pediatr Hematrol Oncol<br>doi as online only:<br>10.1097/MPH.0000000000001426	Preventive Fraction	They estimated the proportion of various health outcomes prevented by an increase in fitness level.
Mayne et al. 2018<br>Circulation<br>138(6):557-566	Preventive Fraction	They estimated the proportion of cardiovascular disease cases that could be prevented by smoke-free policies.
N'Krumah et al. 2016<br>PLoS Negl Trop Dis<br>10(1): e0004327	Preventive Fraction	Unclear. Implies they estimated the proportion of Buruli ulcer cases that could be prevented by various protective strategies.
Salihu et al. 2011<br>J Matern<br>Fetal Neonatal Med<br>24(9):1088-1094	Preventive Fraction	They estimated the proportion of neonatal complications and maternal morbidities that could be prevented with the elimination of maternal obesity.
Seddon et al. 2006<br>Arch Opthalmol<br>124(7):995-1001	Preventive Fraction	They estimated the proportion of age-related macular degeneration that could be prevented by increased omega-3 fatty acid levels consumption.
Trajkova et al. 2017<br>Int J Public Health<br>62(7):775-786	Preventive Fraction	They estimated the burden of stroke that could be prevented if all smokers became former smokers.
Bendich et al. (1997)<br>West J Med<br>166(5):306-312	Preventable Fraction	They estimated the proportion of disease that could have been prevented had vitamin intake been at the recommended levels.
Center for Disease Control<br>MMWR Rcomm Rep<br>44(RR10):1-10	Preventable Fraction	A description on the process of evaluating disease and injury prevention programs.
Fitzgerald et al. (2002)<br>Nutr Cancer<br>43(2):127-132	Preventable Fraction	They estimated the proportion of cancer incidence that could be prevented by improvements in diet quality.
Hagel et al. (2001)<br>Inj Prev<br>7(1):78	Preventable Fraction	A comment on Kopjar (2002)
Kopjar (2002)<br>Inj Prev<br>6(3):235-238	Preventable Fraction	The author estimated the proportion of injuries that could be prevented due to cycling helmet use.*
Mo et al. (2019)<br>BMC Public Health<br>19(1):707	Preventable Fraction	They estimated the proportion of disability-adjusted life years for cardiovascular diseases associated with five scenarios of fruit and vegetable intake.
Norat et al. (2002)<br>Int J Cancer<br>98(2):241-256	Preventable Fraction	They estimated the proportion of colorectal cancer that could be prevented if red meat intake is reduced to 70 g/week.**
Patel et al. (2015)<br>Ann Intern Med<br>163(4):245-253	Preventable Fraction	They estimated the proportion of cardiovascular mortality that could be prevented with the elimination of elevated cholesterol levels,

		diabetes, hypertension, obesity, and smoking.
Peleteiro et al. (2016)<br>Br J Nutr<br>115(5):851-859	Preventable Fraction	They estimated the proportion and absolute number of gastric cancer cases that could have been prevented had fruit and vegetable intake been at the theoretical minimum risk level in 2012.
Tamakoshi et al. (2009)<br>Preventive Medicine<br>48(5):486-492	Preventable Fraction	They estimated the proportion of mortality that could be prevented by improving lifestyle behaviours.
Tominaga (1998)<br>Gan To Kagaku Ryoho<br>25(6):789-796	Preventable Fraction	They estimated the proportion of cancers that could be prevented by reducing smoking levels.
Vainio et al. (2006)<br>Nutr Cancer<br>54(1):111-142	Preventable Fraction	They estimated the proportion of cancers that could be preventable by increasing fruit and vegetable intake.
Andersen et al. (2018)<br>J Trans Health<br>9:217-225	Identified through own reading	They estimated the proportion of disease burden prevented due to cycling behaviour.
Kahlmeier et al. (2017)<br>World Health Organization report	Identified through own reading	The report summarizes the method behind the Health Economic Assessment tool (HEAT) for walking and cycling that can be used to quantify the reduction in mortality risk of current levels of walking or cycling.

*This paper uses the terminology of ‘the proportion of injuries that have been prevented’ due to a given level of cycling helmet use, but further inspection of the formulae presented and the text description confirmed it did not meet the definition of the Prevented Fraction for the Population described in the paper.

**This paper is ambiguous in its language and formulae referred to.

We also selected a random sample of papers in the other categories, across the search terms used, to investigate whether the concept in question was being used. We identified examples across all categories e.g.

Oral health:

Richards D. Substantial reduction in caries from regular fluoride varnish application. Evid Based Dent 2013; 14(3): 72-3.

Vaccine:

Kostova D, Reed C, Finelli L, et al. Influenza Illness and Hospitalizations Averted by Influenza Vaccination in the United States, 2005-2011. PloS one 2013; 8(6): e66312.

Genetic research:

Stutzmann F, Vatin V, Cauchi S, et al. Non-synonymous polymorphisms in melanocortin-4 receptor protect against obesity: the two facets of a Janus obesity gene. Human molecular genetics 2007; 16(15): 1837-44.

Cancer screening:

Stock C, Knudsen AB, Lansdorp-Vogelaar I, Haug U, Brenner H. Colorectal cancer mortality prevented by use and attributable to nonuse of colonoscopy. Gastrointest Endosc 2011; 73(3): 435-43.

Descriptive cancer epidemiology:

Whiteman DC, Webb PM, Green AC, et al. Cancers in Australia in 2010 attributable to modifiable factors: summary and conclusions. Australian and New Zealand Journal of Public Health 2015; 39(5): 477-84.

HIV, sexual health and pregnancy

Steiner MJ, Taylor DJ, Feldblum PJ, Wheeless AJ. How well do male latex condoms work? Pregnancy outcome during one menstrual cycle of use. Contraception 2000;62:315-9.

Suicide and mental health

Knox KL, Litts DA, Talcott GW, Feig JC, Caine ED. Risk of suicide and related adverse outcomes after exposure to a suicide prevention programme in the US Air Force: cohort study. BMJ 2003;327:1376.

Appendix 3: Derivation of alternative Prevented Fraction for the Population formula (Formula 2).

Formulae 1 and 2, both presented in Box 1, are equivalent. Prevented Fraction for the Population is abbreviated to PFP in the following formulae.

$$PFP = \frac{I_u - I_p}{I_u} \qquad \text{(Formula 1)}$$

I_p can be expressed as

$$I_p = P_u \cdot I_u + P_e \cdot I_e \qquad \text{(B)}$$

P_u can be expressed as

$$P_u = 1 - P_e \qquad \text{(C)}$$

And I_e can be expressed as

$$I_e = I_u \cdot RR \qquad \text{(D)}$$

Substituting Formulae C and D into B, I_p can be expressed as

$$I_p = (1 - P_e) \cdot I_u + P_e \cdot I_u \cdot RR \qquad \text{(E)}$$

Substituting Formula E into Formula 1, the PFP can be defined as:

$$PFP = \frac{I_u - ((1 - P_e) \cdot I_u + P_e \cdot I_u \cdot RR)}{I_u} \qquad \text{(F)}$$

This can be rearranged to the following Formulae G and H, eventually simplifying to Formula 2:

$$PFP = \frac{I_u - I_u + P_e \cdot I_u - P_e \cdot I_u \cdot RR}{I_u} \qquad \text{(G)}$$

$$PFP = \frac{P_e \cdot I_u \ (1 - RR)}{I_u} \qquad \text{(H)}$$

$$PFP = P_e(1 - RR) \qquad \text{(Formula 2)}$$

Appendix 4: Derivation of ‘adjusted’ Prevented Fraction for the Population formula (Formula 3)

There are two stages to the derivation of this formula: calculating P_d from P_e and then substituting that into Formula 2. Prevented Fraction for the Population is abbreviated to PFP in the following formulae.

The prevalence of activity amongst cases (P_d) is defined as the number of cases that were active over the total number of cases:

$$P_d = (P_e \cdot I_e \cdot n)/((P_e \cdot I_e \cdot n) + (P_u \cdot I_u \cdot n)) \quad \text{(A)}$$

Divide numerator and denominator by I_u, cancel out n, replace P_u as $1\text{-}P_e$

$$P_d = (P_e \cdot RR)/((P_e \cdot RR) + (1 - P_e)) \quad \text{(Formula 4)}$$

Rearrange this to solve for P_e

$$P_d(P_e \cdot RR - P_e + 1) = P_e \cdot RR \quad \text{(B)}$$

$$P_e\left(P_d \cdot RR - P_d + \frac{P_d}{P_e}\right) = P_e \cdot RR \quad \text{(C)}$$

$$P_d \cdot RR - P_d - RR = -\frac{P_d}{P_e} \quad \text{(D)}$$

$$P_e = \frac{P_d}{(1-(1-P_d)(1-RR))} \quad \text{(E)}$$

Now substitute into Formula 2:

$$PFP = \frac{P_d(1-RR)}{(1-(1-P_d)(1-RR))} \quad \text{(Formula 3)}$$

Appendix 5: Study Protocol

<u>Final analysis plan – confirmed 24/04/19*</u>

Aim: to estimate the Prevented Fraction for the Population from physical activity for all-cause mortality for 168 countries

Data sources: prevalence data from Guthold et al. (2018); relative risks from Lee et al. (2012); number of premature deaths in country from United Nations (downloaded 2019).

Formula used in Monte-Carlo simulations:

- Adjusted Prevented Fraction for the Population, zero counterfactual (main analyses)
- Unadjusted Prevented Fraction for the Population, zero counterfactual
- Adjusted Prevented Fraction for the Population, minimum plausible risk counterfactual

Minimum plausible risk determined by median of the lowest observed prevalence of activity in each region*.

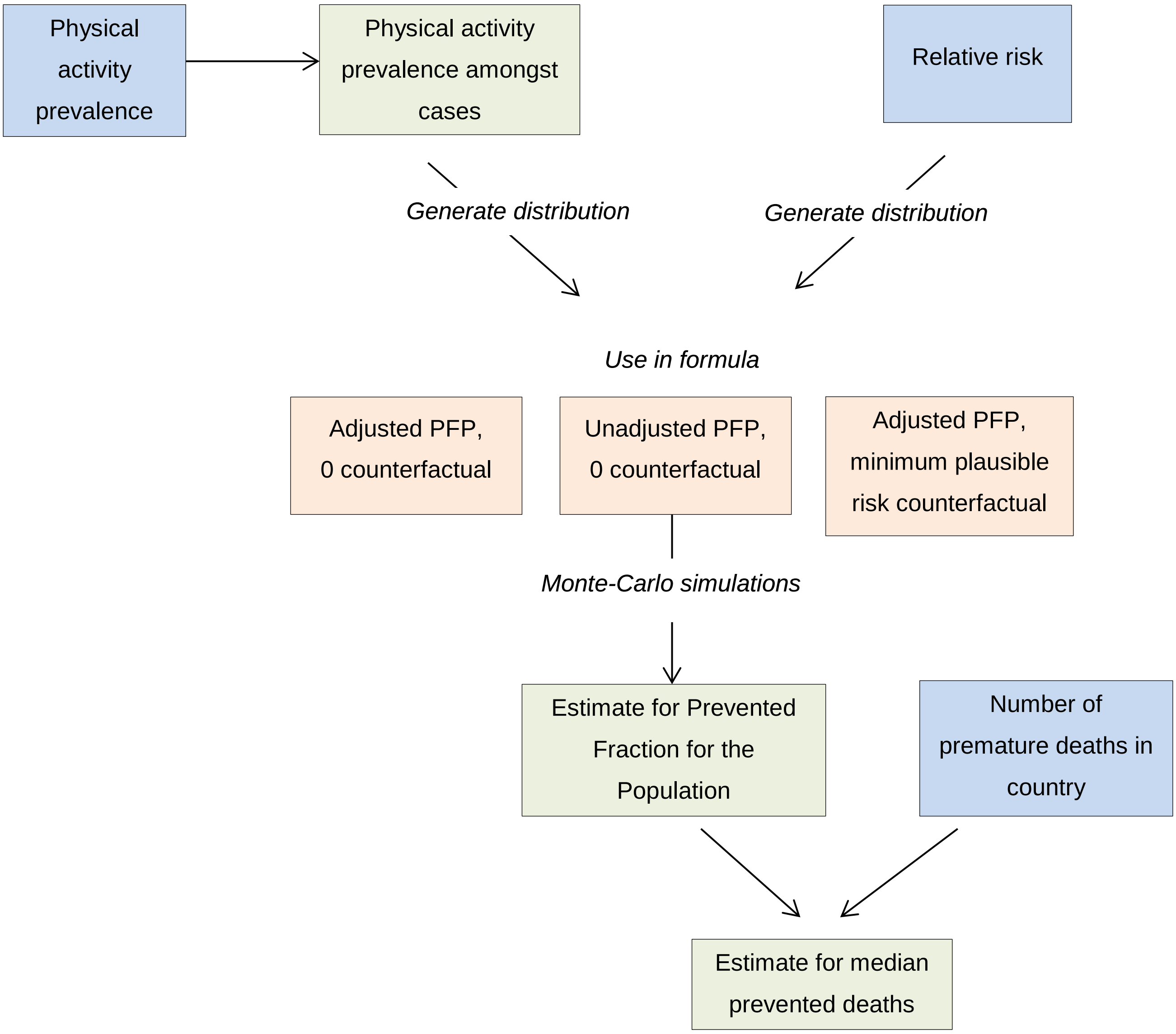

*****the decision to use this counterfactual rather than the lowest observed national prevalence was made in July 2019 following discussions within the author team. The reasons for this decision are explained in the manuscript.

Modifications made to the analysis plan 2017-2019:

Version 1:

- Unadjusted and adjusted analyses
- Zero counterfactual
- All cause mortality and coronary heart disease
- No prediction of number of deaths
- Using prevalence data from Ding et al. (2016)
- Using relative risks from Lee et al. (2012) and Sattlemair et al. (2011)

Version 2:

- Add in a non-zero counterfactual because of concerns that 0% of the population meeting the physical activity guidelines is not realistic.
- To be conservative in the estimates of the benefits of physical activity, we considered a 10% level as a counterfactual.
- We looked at using adjusted and unadjusted non-zero counterfactual formulae

Version 3:

- We could not find justification for a 10% counterfactual, or a reasonable justification for any absolute level.
- We therefore decided to use the minimum observed levels for a country, and term this the minimum plausible risk.
- Guthold et al. (2018) published more up to date, harmonised, prevalence data for more nations so we changed our data source
- We removed the coronary heart disease analyses to streamline the paper's results
- We removed the unadjusted non-zero counterfactual results to streamline the paper
- We decided to estimate the number of deaths corresponding to the estimated Prevented Fractions for the Population to aid interpretation

Edits made after Review 1 from Lancet Global Health (Feb 2020):

- We added in sex-specific results.
- We confirmed that the mortality data from the United Nations was the most appropriate to use. Although there are other sources of non-communicable disease deaths, they are not by age group

Appendix 6. Overview of method.

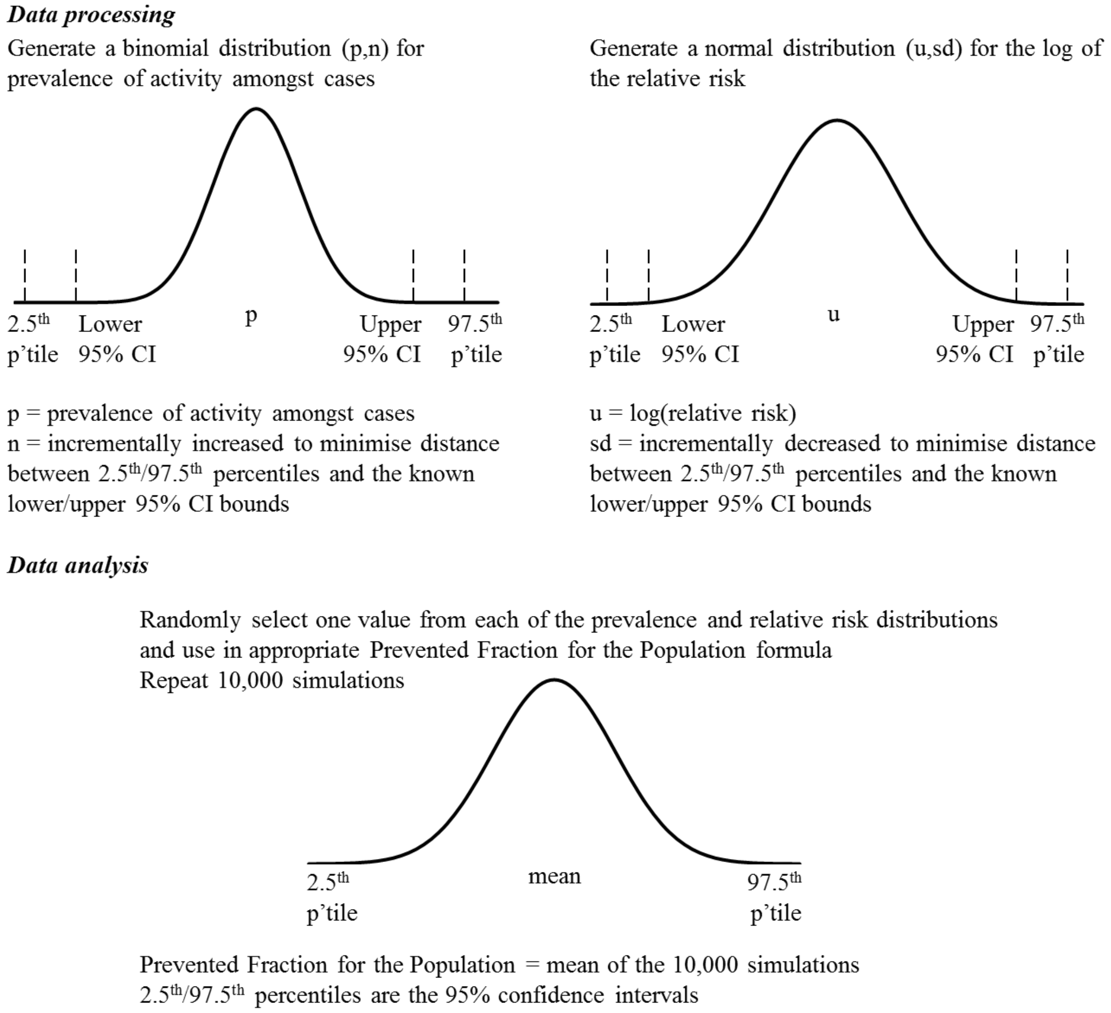

When estimating the 'unadjusted' Prevented Fraction for the Population, the prevalence distribution was the prevalence of activity (P_e).

We did not model any variance around the prevalence of activity (P_e) or prevalence of activity amongst cases (P_d) for the counterfactual scenarios.

Appendix 7. Obtained and generated distributions for the relative risks of all-cause mortality due to physical activity

	Obtained data		Generated distribution	
	RR (95% CI) of inactivity obtained from Lee et al. (2012)	RR (95% CI) for activity	Standard deviation parameter	RR (95% CI) for activity
Unadjusted	1.47 (1.38-1.57)	0.68 (0.64-0.72)	0.04	0.68 (0.63-0.74)
Adjusted	1.28(1.21-1.36)	0.78 (0.74-0.83)	0.04	0.78 (0.72-0.85)

Appendix 8. Obtained and generated prevalence distributions for the 168 countries.

World Health Organization region	World Bank Income classification	Country	Data from Guthold et al. (2018)		Generated distribution		Prevalence of activity amongst those that died (%), (95% CI)	Generated distribution	
			Year	Prevalence of activity (%), (95% CI)	n for binomial distribution	Prevalence of activity (%), (95% CI)		n for binomial distribution	Prevalence of activity (%), (95% CI)
Africa									
	High								
		Seychelles	2013	81.2 (74.8-86.2)	138	81.2 (74.6-87.7)	74.6 (66.9-80.9)	117	74.6 (66.7-82.1)
	Upper-middle								
		Algeria	2003	66.4 (57.3-74.5)	100	66.4 (57.0-75.0)	57.3 (47.7-66.5)	93	57.4 (47.3-66.7)
		Botswana	2014	78.3 (72.5-83.1)	192	78.3 (72.4-83.9)	71.0 (64.2-77.0)	161	71.0 (64.0-77.6)
		Gabon	2009	74.7 (66.5-81.5)	107	74.7 (66.4-82.2)	66.8 (57.4-75.0)	93	66.7 (57.0-76.3)
		Mauritius	2003	70.2 (62.2-77.2)	121	70.2 (62.0-78.5)	61.6 (52.8-69.7)	114	61.6 (52.6-70.2)
		Namibia	2003	66.6 (58.9-73.6)	138	66.6 (58.7-74.6)	57.6 (49.4-65.5)	135	57.6 (48.9-65.9)
		South Africa	2009	61.8 (53.2-69.9)	119	61.8 (52.9-70.6)	52.4 (43.6-61.2)	115	52.4 (43.5-61.7)
	Lower-middle								
		Cabo Verde	2007	80.3 (74.7-85.0)	193	80.3 (74.6-86.0)	73.5 (66.8-79.4)	162	73.5 (66.7-80.3)
		Cameroon	2003	71.5 (64.9-77.3)	170	71.5 (64.7-78.2)	63.0 (55.7-69.8)	157	63.0 (55.4-70.7)
		Congo	2003	72.0 (64.5-78.4)	135	72.0 (64.4-79.3)	63.6 (55.3-71.2)	122	63.6 (54.9-72.1)
		Côte d'Ivoire	2005	66.9 (60.6-73.3)	190	66.9 (60.0-73.7)	57.9 (51.1-65.1)	165	57.9 (50.3-65.5)
		Eswatini	2013	72.0 (67.1-76.9)	302	72.0 (66.9-77.2)	63.6 (58.1-69.4)	253	63.6 (57.7-69.6)
		Ghana	2009	78.2 (73.7-82.7)	288	78.2 (73.3-83.0)	70.9 (65.6-76.5)	240	70.9 (65.0-76.7)
		Kenya	2015	84.6 (82.2-86.9)	853	84.6 (82.2-87.0)	78.9 (75.8-81.9)	676	78.9 (75.7-82.0)
		Lesotho	2012	93.7 (91.4-95.5)	430	93.7 (91.4-95.8)	91.0 (87.8-93.5)	312	91.0 (87.8-93.9)
		Mauritania	2006	58.7 (50.8-66.6)	139	58.7 (50.4-66.9)	49.1 (41.2-57.6)	126	49.1 (40.5-57.9)
		Nigeria	2011	72.9 (66.5-78.5)	185	72.9 (66.5-79.5)	64.7 (57.4-71.3)	164	64.7 (57.3-72.0)
		São Tomé and Príncipe	2009	84.5 (79.8-88.3)	227	84.5 (79.7-89.0)	78.8 (72.9-83.7)	180	78.7 (72.8-84.4)
		Zambia	2008	77.9 (73.2-82.7)	259	77.9 (72.6-83.0)	70.6 (65.0-76.5)	207	70.5 (64.3-76.8)

	Low								
		Benin	2015	84.1 (79.4-87.9)	233	84.1 (79.4-88.8)	78.2 (72.4-83.2)	188	78.2 (72.3-84.0)
		Burkina Faso	2013	79.7 (74.5-84.9)	204	79.7 (74.0-85.3)	72.7 (66.5-79.3)	158	72.7 (65.8-79.8)
		Central African Republic	2010	85.7 (81.5-89.1)	264	85.7 (81.4-89.8)	80.3 (75.0-84.8)	211	80.3 (74.9-85.3)
		Chad	2008	76.7 (70.0-83.4)	136	76.7 (69.1-83.8)	69.1 (61.3-77.4)	109	69.1 (60.6-78.0)
		Comoros	2011	85.7 (82.1-89.3)	324	85.7 (81.8-89.5)	80.3 (75.7-85.0)	239	80.3 (75.3-85.4)
		Democratic Republic of the Congo	2005	76.2 (70.0-81.4)	176	76.2 (69.9-82.4)	68.5 (61.3-74.8)	157	68.5 (61.2-75.8)
		Eritrea	2010	77.6 (74.1-81.2)	482	77.6 (73.9-81.3)	70.2 (66.0-74.6)	388	70.2 (65.7-74.7)
		Ethiopia	2015	85.1 (82.4-87.9)	569	85.1 (82.1-88.1)	79.5 (76.1-83.2)	444	79.5 (75.7-83.3)
		Gambia	2010	78.9 (72.9-84.0)	180	78.9 (72.8-84.4)	71.8 (64.7-78.1)	149	71.8 (64.4-79.2)
		Guinea	2009	85.5 (80.9-89.2)	219	85.5 (80.8-90.0)	80.0 (74.2-84.9)	178	80.0 (74.2-86.0)
		Liberia	2011	74.9 (68.7-80.2)	185	74.9 (68.7-81.1)	67.0 (59.9-73.4)	161	67.0 (59.6-73.9)
		Madagascar	2205	82.8 (78.0-86.8)	231	82.8 (77.9-87.5)	76.6 (70.7-81.7)	190	76.6 (70.5-82.6)
		Malawi	2009	84.4 (82.1-86.7)	885	84.4 (82.0-86.8)	78.6 (75.7-81.6)	672	78.6 (75.5-81.7)
		Mali	2007	59.6 (52.7-66.4)	183	59.6 (52.5-66.7)	50.1 (43.1-57.3)	170	50.1 (42.4-57.7)
		Mozambique	2005	94.4 (92.4-95.9)	513	94.4 (92.4-96.3)	92.0 (89.2-94.1)	370	92.0 (89.2-94.6)
		Niger	2007	77.6 (70.4-83.5)	128	77.6 (70.3-84.4)	70.2 (61.8-77.5)	109	70.2 (61.5-78.9)
		Rwanda	2012	85.4 (81.1-88.9)	253	85.4 (81.0-89.7)	79.9 (74.5-84.5)	199	79.9 (74.4-85.4)
		Senegal	2015	76.9 (72.5-81.3)	325	76.9 (72.3-81.5)	69.4 (64.2-74.7)	256	69.4 (63.7-75.0)
		Sierra Leone	2009	85.7 (81.3-89.2)	240	85.7 (81.3-90.0)	80.3 (74.7-84.9)	189	80.3 (74.6-85.7)
		Togo	2011	90.2 (87.2-92.6)	382	90.2 (87.2-93.2)	86.2 (82.2-89.5)	287	86.2 (82.2-90.2)
		Uganda	2014	94.5 (92.4-96.0)	448	94.5 (92.2-96.4)	92.1 (89.2-94.2)	324	92.1 (89.2-95.1)
		United Republic of Tanzania	2011	93.5 (91.4-95.2)	523	93.5 (91.4-95.6)	90.7 (87.8-93.1)	394	90.7 (87.8-93.4)
		Zimbabwe	2003	73.2 (65.7-79.5)	131	73.2 (65.7-80.9)	65.0 (56.6-72.5)	119	65.0 (56.3-73.1)
Americas									

	High								
		Argentina	2013	58.4 (50.0-66.4)	134	58.4 (50.0-66.4)	48.8 (40.5-57.3)	123	48.8 (39.8-57.7)
		Bahamas	2011	56.7 (48.1-65.1)	122	56.7 (47.5-65.6)	47.1 (38.7-55.9)	119	47.1 (37.8-56.3)
		Barbados	2012	57.1 (48.3-65.9)	110	57.1 (48.2-66.4)	47.5 (38.8-56.8)	103	47.5 (37.9-57.3)
		Bermuda	2014	73.8 (65.4-80.8)	106	73.8 (65.1-82.1)	65.7 (56.2-74.1)	93	65.7 (55.9-75.3)
		British Virgin Islands	2009	72.6 (65.1-79.1)	128	72.6 (64.8-80.5)	64.3 (55.9-72.0)	122	64.3 (55.7-73.0)
		Canada	2003	71.4 (63.7-78.1)	123	71.4 (63.4-78.9)	62.9 (54.4-70.8)	120	62.9 (54.2-71.7)
		Cayman Islands	2012	70.9 (62.5-78.1)	106	70.9 (62.3-79.3)	62.4 (53.1-70.8)	104	62.3 (52.9-71.2)
		Chile	2009	73.4 (66.4-80.4)	136	73.4 (66.2-80.9)	65.2 (57.3-73.6)	116	65.3 (56.9-74.1)
		Saint Kitts and Nevis	2007	67.8 (58.8-75.7)	101	67.8 (58.4-76.2)	58.9 (49.2-67.9)	96	58.9 (49.0-68.8)
		Trinidad and Tobago	2011	61.8 (50.4-72.2)	66	61.8 (50.0-72.7)	52.4 (40.9-63.8)	65	52.4 (40.0-64.6)
		United States of America	2014	60.0 (52.7-67.0)	164	60.0 (52.4-67.7)	50.5 (43.1-58.0)	163	50.5 (42.9-58.3)
		Uruguay	2013	77.6 (71.0-83.1)	151	77.6 (70.9-84.1)	70.2 (62.5-77.0)	127	70.2 (62.2-78.0)
	Upper-middle								
		Bolivarian Republic of Venezuela	2008	68.6 (60.0-76.0)	112	68.6 (59.8-76.8)	59.8 (50.5-68.3)	100	59.8 (50.0-69.0)
		Brazil	2016	53.0 (44.7-61.1)	133	53.0 (44.4-61.7)	43.4 (35.5-51.6)	131	43.4 (35.1-51.9)
		Colombia	2010	56.0 (47.3-64.6)	119	56.0 (47.1-64.7)	46.4 (37.9-55.4)	111	46.4 (36.9-55.9)
		Costa Rica	2014	53.9 (45.6-62.1)	133	53.9 (45.1-62.4)	44.3 (36.3-52.7)	130	44.3 (36.2-53.1)
		Cuba	2010	63.1 (54.8-70.8)	123	63.1 (54.5-71.5)	53.8 (45.2-62.2)	125	53.8 (44.8-62.4)
		Dominica	2007	78.4 (72.0-83.7)	160	78.4 (71.9-84.4)	71.2 (63.6-77.7)	131	71.2 (63.4-78.6)
		Dominican Republic	2003	61.0 (52.3-69.1)	119	61.0 (52.1-69.8)	51.5 (42.7-60.3)	116	51.5 (42.2-60.3)
		Ecuador	2013	72.8 (65.4-80.3)	120	72.8 (65.0-80.8)	64.5 (56.2-73.5)	97	64.5 (54.6-74.2)
		Grenada	2011	71.3 (62.8-78.6)	104	71.3 (62.5-79.8)	62.8 (53.4-71.4)	98	62.8 (53.1-72.5)
		Guatemala	2003	62.9 (52.6-72.1)	84	62.9 (52.4-72.6)	53.5 (43.0-63.7)	78	53.5 (42.3-64.1)

		Jamaica	2007	67.4 (59.2-74.8)	122	67.4 (59.0-75.4)	58.4 (49.7-66.9)	121	58.4 (49.6-66.9)
		Mexico	2016	71.1 (64.4-77.0)	176	71.1 (64.2-77.8)	62.6 (55.2-69.5)	153	62.6 (54.9-69.9)
		Paraguay	2011	62.6 (54.1-71.1)	113	62.6 (54.0-71.7)	53.2 (44.5-62.6)	103	53.2 (43.7-63.1)
		Saint Lucia	2012	60.2 (50.1-69.7)	88	60.2 (50.0-70.5)	50.7 (40.6-61.0)	81	50.7 (39.5-61.7)
		Suriname	2013	55.6 (47.3-63.6)	130	55.6 (46.9-63.9)	46.0 (37.9-54.3)	128	46.0 (37.5-54.7)
Eastern Mediterranean									
	High								
		Kuwait	2014	33.0 (25.7-41.4)	115	33.0 (24.4-41.7)	25.1 (19.0-32.4)	132	25.1 (18.2-32.6)
		Oman	2010	67.1 (58.2-74.8)	102	67.1 (57.8-76.5)	58.1 (48.6-66.9)	105	58.1 (48.6-67.6)
		Qatar	2012	63.2 (53.8-71.6)	99	63.2 (53.5-72.7)	53.9 (44.2-63.2)	94	53.9 (43.6-63.8)
		Saudi Arabia	2010	46.9 (38.4-55.5)	121	46.9 (38.0-56.2)	37.5 (29.8-45.9)	119	37.5 (28.6-46.2)
		United Arab Emirates	2012	58.6 (50.1-67.2)	117	58.6 (49.6-67.5)	49.0 (40.6-58.2)	109	49.0 (39.5-58.7)
	Upper-middle								
		Iraq	2015	48.0 (40.2-55.9)	144	48.0 (39.6-56.3)	38.6 (31.4-46.3)	144	38.6 (30.6-46.5)
		Islamic Republic of Iran	2011	66.8 (58.6-74.1)	120	66.8 (58.3-75.0)	57.8 (49.0-66.0)	115	57.8 (48.7-67.0)
		Jordan	2007	88.1 (83.6-91.6)	201	88.1 (83.6-92.5)	83.4 (77.6-88.1)	151	83.4 (77.5-89.4)
		Lebanon	2008	63.6 (54.6-71.7)	105	63.6 (54.3-72.4)	54.3 (45.0-63.3)	99	54.3 (44.4-63.6)
		Libya	2009	63.6 (54.3-72.0)	98	63.6 (54.1-73.5)	54.3 (44.7-63.6)	93	54.3 (44.1-64.5)
	Lower-middle								
		Egypt	2011	69.0 (60.5-76.4)	108	69.0 (60.2-77.8)	60.2 (51.0-68.8)	108	60.2 (50.9-69.4)
		Morocco	2008	73.8 (64.6-81.4)	84	73.8 (64.3-83.3)	65.7 (55.4-74.8)	73	65.7 (54.8-76.7)
		Pakistan	2014	66.3 (53.0-79.7)	38	66.3 (50.0-81.6)	57.2 (43.4-72.7)	32	57.2 (40.6-75.0)
		Occupied Palestine Territory	2010	59.5 (51.0-67.5)	122	59.5 (50.8-68.0)	50.0 (41.4-58.5)	122	50.0 (41.0-59.0)
		Tunisia	2003	69.6 (61.3-76.9)	113	69.6 (61.1-77.9)	60.9 (51.9-69.4)	103	60.9 (51.5-69.9)
Europe									

	High								
		Andorra	2004	61.6 (53.4-69.2)	128	61.6 (53.1-69.5)	52.2 (43.8-60.4)	129	52.2 (43.4-60.5)
		Austria	2013	69.9 (63.2-75.9)	173	69.9 (63.0-76.3)	61.2 (53.9-68.2)	162	61.2 (53.7-68.5)
		Belgium	2013	64.3 (57.2-70.7)	165	64.3 (57.0-71.5)	55.0 (47.6-62.1)	167	55.0 (47.3-62.9)
		Croatia	2013	68.9 (61.0-75.9)	130	68.9 (60.8-76.9)	60.1 (51.5-68.2)	121	60.1 (51.2-68.6)
		Cyprus	2013	55.6 (47.9-63.2)	151	55.6 (47.7-63.6)	46.0 (38.5-53.9)	142	46.0 (38.0-54.2)
		Czechia	2013	68.9 (61.2-75.8)	136	68.9 (61.0-76.5)	60.1 (51.7-68.0)	128	60.1 (51.6-68.8)
		Denmark	2013	71.5 (65.0-77.3)	182	71.5 (64.8-78.0)	63.0 (55.8-69.8)	162	63.0 (55.6-70.4)
		Estonia	2013	68.0 (60.2-74.9)	135	68.0 (60.0-75.6)	59.1 (50.7-67.0)	125	59.1 (50.4-68.0)
		Finland	2013	83.4 (79.0-87.1)	266	83.4 (79.0-87.6)	77.4 (71.9-82.1)	216	77.4 (71.8-82.9)
		France	2013	70.7 (64.1-76.5)	175	70.7 (64.0-77.1)	62.1 (54.8-68.9)	163	62.1 (54.6-69.3)
		Germany	2016	57.8 (50.8-64.5)	178	57.8 (50.6-65.2)	48.2 (41.2-55.3)	180	48.2 (41.1-55.6)
		Greece	2013	62.3 (55.1-69.1)	171	62.3 (55.0-69.6)	52.9 (45.5-60.3)	160	52.9 (45.0-60.6)
		Hungary	2013	61.5 (53.2-69.1)	123	61.5 (52.9-69.9)	52.1 (43.6-60.3)	125	52.1 (43.2-60.8)
		Ireland	2015	67.3 (60.5-73.5)	174	67.3 (60.3-74.1)	58.3 (51.0-65.3)	171	58.3 (50.9-65.5)
		Italy	2013	58.6 (51.2-65.7)	161	58.6 (50.9-65.8)	49.0 (41.6-56.6)	160	49.0 (41.3-56.9)
		Latvia	2013	70.5 (62.7-77.2)	128	70.5 (62.5-78.1)	61.9 (53.3-69.7)	117	61.9 (53.0-70.9)
		Lithuania	2013	73.5 (66.4-79.5)	142	73.5 (66.2-80.3)	65.3 (57.3-72.5)	128	65.4 (57.0-73.4)
		Luxembourg	2013	71.6 (65.1-77.3)	183	71.6 (65.0-78.1)	63.2 (55.9-69.8)	167	63.1 (55.7-70.1)
		Malta	2013	58.3 (49.7-66.4)	125	58.3 (49.6-67.2)	48.7 (40.2-57.3)	123	48.7 (39.8-57.7)
		Netherlands	2013	72.8 (66.5-78.4)	197	72.8 (66.5-78.7)	64.5 (57.4-71.2)	166	64.5 (57.2-71.7)
		Norway	2014	68.3 (61.3-74.6)	162	68.3 (61.1-75.3)	59.4 (51.9-66.6)	155	59.4 (51.6-67.1)
		Poland	2013	67.5 (59.5-74.7)	125	67.5 (59.2-75.2)	58.5 (50.0-66.8)	119	58.5 (49.6-67.2)
		Portugal	2013	56.6 (47.8-65.0)	114	56.6 (47.4-65.8)	47.0 (38.4-55.8)	112	47.0 (37.5-56.3)
		Slovakia	2013	65.1 (57.0-72.4)	127	65.1 (56.7-73.2)	55.9 (47.4-64.1)	125	55.9 (47.2-64.8)
		Slovenia	2013	67.8 (59.9-74.8)	129	67.8 (59.7-76.0)	58.9 (50.4-66.9)	120	58.9 (50.0-67.5)
		Spain	2013	73.2 (66.8-78.8)	180	73.2 (66.7-79.4)	65.0 (57.8-71.6)	163	65.0 (57.7-72.4)
		Sweden	2013	76.9 (71.1-81.8)	200	76.9 (71.0-82.5)	69.4 (62.6-75.3)	173	69.3 (62.4-76.3)
		Switzerland	2012	76.3 (69.7-81.8)	158	76.3 (69.6-82.9)	68.6 (61.0-75.3)	135	68.6 (60.7-76.3)

		United Kingdom	2013	64.1 (57.1-70.6)	174	64.1 (56.9-71.3)	54.8 (47.5-62.0)	170	54.8 (47.1-62.4)
	Upper-middle								
		Armenia	2016	77.4 (69.5-83.6)	104	77.4 (69.2-85.6)	70.0 (60.8-77.6)	91	69.9 (60.4-79.1)
		Belarus	2016	85.9 (80.5-90.0)	153	85.9 (80.4-91.5)	80.6 (73.7-86.0)	125	80.5 (73.6-87.2)
		Bosnia and Herzegovina	2003	74.5 (66.1-81.5)	103	74.5 (66.0-82.5)	66.5 (57.0-75.0)	90	66.5 (56.7-75.6)
		Bulgaria	2013	61.4 (52.7-69.4)	120	61.4 (52.5-70.0)	52.0 (43.1-60.7)	115	52.0 (42.6-60.9)
		Kazakhstan	2003	72.5 (63.2-80.2)	86	72.5 (62.8-81.4)	64.2 (53.9-73.4)	75	64.2 (53.3-74.7)
		Romania	2013	64.6 (56.3-72.2)	118	64.6 (55.9-72.9)	55.4 (46.7-63.8)	121	55.4 (46.3-64.5)
		Russian Federation	2008	82.9 (78.2-87.6)	216	82.9 (77.8-88.0)	76.7 (70.9-82.8)	167	76.7 (70.1-83.2)
		Serbia	2006	60.5 (51.2-69.2)	102	60.5 (51.0-69.6)	51.0 (41.6-60.4)	100	51.0 (41.0-61.0)
		Turkey	2005	69.4 (61.2-76.7)	115	69.4 (60.9-77.4)	60.7 (51.7-69.1)	109	60.7 (51.4-69.7)
	Lower-middle								
		Georgia	2016	82.0 (75.4-87.2)	121	82.0 (75.2-88.4)	75.6 (67.6-82.2)	104	75.6 (67.3-83.7)
		Kyrgyzstan	2013	86.1 (79.5-90.9)	107	86.1 (79.4-92.5)	80.8 (72.5-87.2)	79	80.8 (72.2-88.6)
		Republic of Moldova	2013	88.5 (84.0-91.9)	193	88.5 (83.9-92.8)	84.0 (78.1-88.5)	150	84.0 (78.0-89.3)
		Ukraine	2003	80.4 (73.6-85.8)	128	80.4 (73.4-86.7)	73.6 (65.5-80.4)	112	73.6 (65.2-81.3)
		Uzbekistan	2014	80.9 (73.3-86.8)	101	80.9 (73.3-88.1)	74.2 (65.1-81.7)	85	74.2 (64.7-83.5)
	Low								
		Tajikistan	2016	70.7 (61.2-78.8)	87	70.7 (60.9-80.5)	62.1 (51.7-71.6)	78	62.1 (51.3-73.1)
South-East Asia									
	Upper-middle								
		Maldives	2011	69.7 (59.9-78.0)	79	69.7 (59.5-79.8)	61.0 (50.4-70.7)	76	61.0 (50.0-71.1)
		Thailand	2016	75.4 (67.7-81.8)	117	75.4 (67.5-82.9)	67.6 (58.8-75.3)	106	67.6 (58.5-76.4)
	Lower-middle								
		Bangladesh	2009	72.2 (60.9-83.6)	47	72.2 (59.6-85.1)	63.8 (51.4-77.6)	39	63.9 (48.7-79.5)

		Bhutan	2014	77.0 (72.5-81.6)	292	77.0 (72.3-81.9)	69.5 (64.2-75.1)	239	69.5 (63.6-75.3)
		India	2014	66.0 (52.3-77.7)	41	66.0 (51.2-80.5)	56.9 (42.7-70.3)	41	56.9 (41.5-70.7)
		Indonesia	2006	77.4 (69.1-84.0)	93	77.4 (68.8-86.0)	70.0 (60.3-78.1)	85	70.0 (60.0-80.0)
		Myanmar	2014	89.3 (85.0-92.5)	200	89.3 (85.0-93.5)	85.0 (79.4-89.3)	150	85.0 (79.3-90.7)
		Sri Lanka	2014	71.1 (62.1-78.7)	94	71.1 (61.7-79.8)	62.6 (52.7-71.5)	88	62.6 (52.3-72.7)
		Timor-Leste	2014	82.2 (75.0-87.7)	108	82.2 (75.0-88.9)	75.8 (67.1-82.9)	91	75.9 (67.0-84.6)
	Low								
		Nepal	2013	86.6 (84.4-88.8)	847	86.6 (84.3-88.9)	81.5 (78.6-84.4)	644	81.5 (78.4-84.5)
Western Pacific									
	High								
		Australia	2003	69.6 (62.1-76.3)	139	69.6 (61.9-77.0)	60.9 (52.7-68.6)	128	60.9 (52.3-69.5)
		Brunei Darussalam	2015	72.7 (61.7-81.5)	62	72.7 (61.3-83.9)	64.4 (52.3-75.0)	56	64.4 (51.8-76.8)
		French Polynesia	2010	82.1 (75.6-87.2)	135	82.1 (75.6-88.2)	75.7 (67.8-82.2)	108	75.7 (67.6-83.3)
		Japan	2003	64.5 (46.2-79.5)	27	64.5 (44.4-81.5)	55.3 (36.9-72.5)	26	55.3 (34.6-73.1)
		New Zealand	2015	57.6 (50.2-64.7)	164	57.6 (50.0-65.2)	48.0 (40.7-55.5)	165	48.0 (40.6-55.8)
		Palau	2016	59.1 (48.7-68.9)	79	59.1 (48.1-69.6)	49.6 (39.2-60.1)	79	49.5 (38.0-60.8)
		Republic of Korea	2013	64.6 (47.1-79.1)	26	64.6 (46.2-80.8)	55.4 (37.7-72.0)	28	55.4 (35.7-75.0)
		Singapore	2013	63.5 (45.7-78.3)	25	63.5 (44.0-80.0)	54.2 (36.4-71.0)	26	54.2 (34.6-73.1)
	Upper-middle								
		American Samoa	2004	46.6 (35.0-58.6)	64	46.6 (34.4-59.4)	37.2 (26.8-49.0)	63	37.2 (25.4-49.2)
		China	2013	85.9 (80.6-89.9)	164	85.9 (80.5-90.9)	80.6 (73.9-85.8)	133	80.5 (73.7-87.2)
		Cook Islands	2012	81.5 (75.1-86.7)	140	81.5 (75.0-87.9)	75.0 (67.2-81.6)	112	75.0 (67.0-83.0)
		Fiji	2011	82.6 (76.4-87.5)	139	82.6 (76.3-88.5)	76.3 (68.8-82.6)	118	76.4 (68.6-83.9)
		Malaysia	2015	61.2 (51.4-70.3)	90	61.2 (51.1-71.1)	51.7 (41.8-61.7)	90	51.7 (41.1-62.2)
		Marshall Islands	2002	56.5 (46.3-66.2)	83	56.5 (45.8-67.5)	46.9 (37.0-57.1)	85	46.9 (36.5-57.7)
		Nauru	2015	57.9 (46.7-68.3)	75	57.9 (46.7-69.3)	48.3 (37.3-59.4)	68	48.3 (36.8-60.3)

		Samoa	2013	87.4 (82.3-91.3)	163	87.4 (82.2-92.0)	82.5 (76.0-87.7)	124	82.5 (75.8-88.7)
		Tonga	2011	82.6 (76.4-87.6)	139	82.6 (76.3-88.5)	76.3 (68.8-82.8)	118	76.3 (68.6-83.9)
		Tuvalu	2015	72.7 (64.6-79.5)	112	72.7 (64.3-80.4)	64.4 (55.4-72.5)	100	64.4 (55.0-74.0)
	Lower-middle								
		Cambodia	2010	89.5 (84.3-93.1)	133	89.5 (84.2-94.7)	85.3 (78.5-90.2)	106	85.3 (78.3-91.5)
		Federated States of Micronesia	2016	63.4 (54.0-71.8)	95	63.4 (53.7-72.6)	54.1 (44.4-63.4)	98	54.1 (43.9-64.3)
		Kiribati	2004	59.6 (49.8-68.8)	91	59.6 (49.5-69.2)	50.1 (40.3-60.0)	91	50.1 (39.6-60.4)
		Lao People's Democratic Republic	2013	83.7 (79.7-87.7)	291	83.7 (79.4-88.0)	77.7 (72.7-82.9)	221	77.7 (72.0-83.3)
		Mongolia	2013	81.4 (75.6-86.1)	176	81.4 (75.6-86.9)	74.8 (67.8-80.8)	142	74.9 (67.6-81.7)
		Niue	2011	93.1 (90.1-95.2)	272	93.1 (90.1-96.0)	90.2 (86.1-93.1)	201	90.2 (86.1-94.0)
		Papua New Guinea	2007	85.2 (78.1-90.3)	95	85.2 (77.9-91.6)	79.7 (70.8-86.4)	78	79.7 (70.5-88.5)
		Philippines	2015	60.3 (51.4-68.7)	106	60.3 (50.9-69.8)	50.8 (41.8-59.9)	106	50.8 (41.5-60.4)
		Solomon Islands	2015	81.8 (75.2-87.0)	133	81.8 (75.2-88.0)	75.3 (67.3-82.0)	113	75.3 (67.3-83.2)
		Tokelau	2014	88.9 (83.9-92.5)	155	88.9 (83.9-93.6)	84.5 (78.0-89.3)	118	84.5 (78.0-90.7)
		Vanuatu	2011	92.0 (88.4-94.6)	215	92.0 (88.4-95.4)	88.7 (83.8-92.3)	166	88.7 (83.7-93.4)
		Viet Nam	2015	74.6 (66.9-81.1)	120	74.6 (66.7-82.5)	66.6 (57.9-74.5)	106	66.6 (57.6-75.5)

Appendix 9. Population and mortality data

Mortality data obtained from

https://population.un.org/wpp/Download/Archive/Standard/ (2017 Revision)

Population data obtained from https://population.un.org/wpp/Download/Files/1_Indicators%20(Standard)/EXCEL_FILES/1_Population/WPP2019_POP_F01_1_TOTAL_POPULATION_BOTH_SEXES.xlsx

Total world population (2015): 7,379,797,139 (7.4 billion)

Population (2015) for the 168 countries with physical activity data to be included in the present study: 7,022,019,600 (7.0 billion; 95.2% of the world population)

Population (2015) for the 14 countries for which there were no mortality data: 527,820 (<0.01% of total world population). We imputed mortality data for these countries by using the median ratio of deaths between the ages of 40-74 years to total population of countries in the same WHO region and income group. We multiplied this median value by the population of the country for which there was no mortality data. These 14 countries were:

American Samoa
Andorra
Bermuda
British Virgin Islands
Cayman Islands
Cook Islands
Dominica
Marshall Islands
Nauru
Niue
Palau
Saint Kitts and Nevis
Tokelau
Tuvalu

Appendix 10: derivation of 'adjusted' Prevented Fraction for the Population formula for non-zero counterfactuals

Prevented Fraction for the Population is abbreviated to PFP. The denotation of ' indicates a counterfactual.

The Potential Impact Fraction formula relates closely to that of the Population Attributable Fraction:

$$Population\ Attributable\ Fraction = \frac{I_p - I_u}{I_p} \qquad \text{(A)}$$

Dictionary of Epidemiology

$$Potential\ Impact\ Fraction = \frac{I_p - I_{p'}}{I_p} \qquad \text{(B)}$$

Morgenstern and Bursic 1982; Benichou 2001

Note: these two formulae define the exposure as harmful and unexposure as protective.

The Potential Impact Fraction is an extension of the Population Attributable Fraction formula that replaces the incidence rate in the unexposed with a counterfactual incidence rate.

The same can be done for the PFP.

$$PFP = \frac{I_u - I_p}{I_u} \qquad \text{(C)}$$

$$'unadjusted'PFP\ for\ a\ non-zero\ counterfactual = \frac{I_{p'} - I_p}{I_{p'}} \qquad \text{(D)}$$

The proportion of active in the counterfactual population is represented by $P_{e'}$. Formula A can be re-written as:

$$'unadjusted'PFP\ for\ a\ non-zero\ counterfactual =$$

$$\frac{\left((1-P_{e'})\cdot I_u + P_{e'}\cdot I_u\cdot RR\right) - \left((1-P_e)\cdot I_u + P_e\cdot I_u\cdot RR\right)}{\left((1-P_{e'})\cdot I_u + P_{e'}\cdot I_u\cdot RR\right)} \qquad \text{(E)}$$

This can be rearranged as:

$$'unadjusted'PFP\ for\ a\ non-zero\ counterfactual = \frac{(P_e - P_{e'})(1-RR)}{1-P_{e'}(1-RR)} \qquad \text{(F)}$$

Formulae F and B both compare the difference in disease burden between two scenarios, and express that as a proportion of the scenario with the higher disease burden. Therefore, they can be considered equivalent but differing in terms of exposure definition (harmful or protective).

To extend this formula to use P_d rather than Pe, substitute in a variant of Formula 4 (P_e defined in terms of P_d and RR):

$$P_e = \frac{P_d}{(1-(1-P_d)(1-RR))}$$ (a variant of Formula 4, see Appendix 4, p9)

$$'adjusted' PFP\ for\ a\ non-zero\ counterfactual = \frac{\left(\frac{P_d}{\left(1-(1-P_d)\cdot(1-RR)\right)} - \frac{P_{d'}}{\left(1-\left(1-P_{d'}\right)\cdot(1-RR)\right)}\right)(1-RR)}{1-\frac{P_{d'}\cdot(1-RR)}{\left(1-\left(1-P_{d'}\right)\cdot(1-RR)\right)}} \quad \text{(G)}$$

The numerator of formula G can be rearranged:

$$'adjusted' PFP\ for\ a\ non-zero\ counterfactual =$$

$$\frac{\frac{P_d}{\left(1-(1-P_d)\cdot(1-RR)\right)} - \frac{P_d\cdot RR}{\left(1-(1-P_d)\cdot(1-RR)\right)} - \frac{P_{d'}}{\left(1-(1-P_{d'})\cdot(1-RR)\right)} + \frac{P_{d'}\cdot RR}{\left(1-(1-P_{d'})\cdot(1-RR)\right)}}{1-\frac{P_{d'}\cdot(1-RR)}{\left(1-\left(1-P_{d'}\right)\cdot(1-RR)\right)}} \quad \text{(H)}$$

Next, multiply the numerator by the denominator:

$$'adjusted' PFP\ for\ a\ non-zero\ counterfactual =$$

$$\left(\frac{P_d}{(1-(1-P_d)\cdot(1-RR))} - \frac{P_d\cdot RR}{(1-(1-P_d)\cdot(1-RR))} - \frac{P_{d'}}{(1-(1-P_{d'})\cdot(1-RR))} + \frac{P_{d'}\cdot RR}{(1-(1-P_{d'})\cdot(1-RR))}\right)\cdot\frac{(1-(1-P_{d'})\cdot(1-RR))}{\left((1-(1-P_{d'})\cdot(1-RR))+P_{d'}\cdot RR-P_{d'}\right)} \quad \text{(I)}$$

This can be expanded out and cancelled to:

$$'adjusted' PFP\ for\ a\ non-zero\ counterfactual =$$

$$\frac{P_d\cdot(1-RR)\cdot\left(1-(1-P_{d'})\cdot(1-RR)\right)}{\left(1-(1-P_d)\cdot(1-RR)\right)\cdot\left((1-(1-P_{d'})\cdot(1-RR))+P_{d'}\cdot RR-P_{d'}\right)} - \frac{P_{d'}(1-RR)}{\left((1-(1-P_{d'})\cdot(1-RR))+P_{d'}\cdot RR-P_{d'}\right)} \quad \text{(J)}$$

Multiply out to get both fractions on the same denominator

$$'adjusted' PFP\ for\ a\ non-zero\ counterfactual =$$

$$\frac{P_d\cdot(1-RR)\cdot\left(1-(1-P_{d'})\cdot(1-RR)\right)-P_{d'}\cdot(1-RR)\cdot\left(1-(1-P_d)\cdot(1-RR)\right)}{(1-(1-P_d)\cdot(1-RR))\cdot\left((1-(1-P_{d'})\cdot(1-RR))+P_{d'}\cdot RR-P_{d'}\right)} \quad \text{(K)}$$

The numerator of formula K expands and cancels to:

$$Numerator(K) =$$

$$(P_d - P_d \cdot RR) \cdot (P_{d'} + RR + P_{d'} \cdot RR) - (P_{d'} - P_{d'} \cdot RR) \cdot (P_d + RR + P_d \cdot RR) \qquad \text{(L)}$$

$Numerator(K) =$

$$P_d \cdot P_{d'} + P_d \cdot RR + P_d \cdot P_{d'} \cdot RR - P_d \cdot P_{d'} \cdot RR - P_d RR^2 - P_d \cdot P_{d'} \cdot RR^2 - P_d \cdot P_{d'} - P_{d'} \cdot RR - P_d \cdot P_{d'} \cdot RR + P_d \cdot P_{d'} \cdot RR + P_{d'} RR^2 + P_d \cdot P_{d'} \cdot RR^2 \qquad \text{(M)}$$

$$Numerator(K) = P_d \cdot RR - P_d RR^2 - P_{d'} \cdot RR + P_{d'} RR^2 \qquad \text{(N)}$$

$$Numerator(K) = RR(P_d - P_d \cdot RR - P_{d'} + P_{d'} RR) \qquad \text{(O)}$$

The denominator of formula K can be partially expanded out to:

$$Denominator(K) = \big(1 - (1 - P_d) \cdot (1 - RR)\big) \cdot (P_{d'} + RR - P_{d'} \cdot RR + P_{d'} \cdot RR - P_{d'}) \qquad \text{(P)}$$

$$Denominator(K) = \big(1 - (1 - P_d) \cdot (1 - RR)\big) \cdot RR \qquad \text{(Q)}$$

Together,

$$'adjusted' PFP\ for\ a\ non-zero\ counterfactual = \frac{RR(P_d - P_d \cdot RR - P_{d'} + P_{d'} RR)}{\big(1 - (1 - P_d) \cdot (1 - RR)\big) \cdot RR} \qquad \text{(R)}$$

This cancels and simplifies to

$$'adjusted' PFP\ for\ a\ non-zero\ counterfactual = \frac{(P_d - P_{d'}) \cdot (1 - RR)}{\big(1 - (1 - P_d) \cdot (1 - RR)\big)} \qquad \text{(Formula 5)}$$

Appendix 11. The adjusted Prevented Fractions for the Population for 168 countries, representing the percentage of premature mortality averted due to current physical activity levels, by World Health Organization region and World Bank Income Classification.

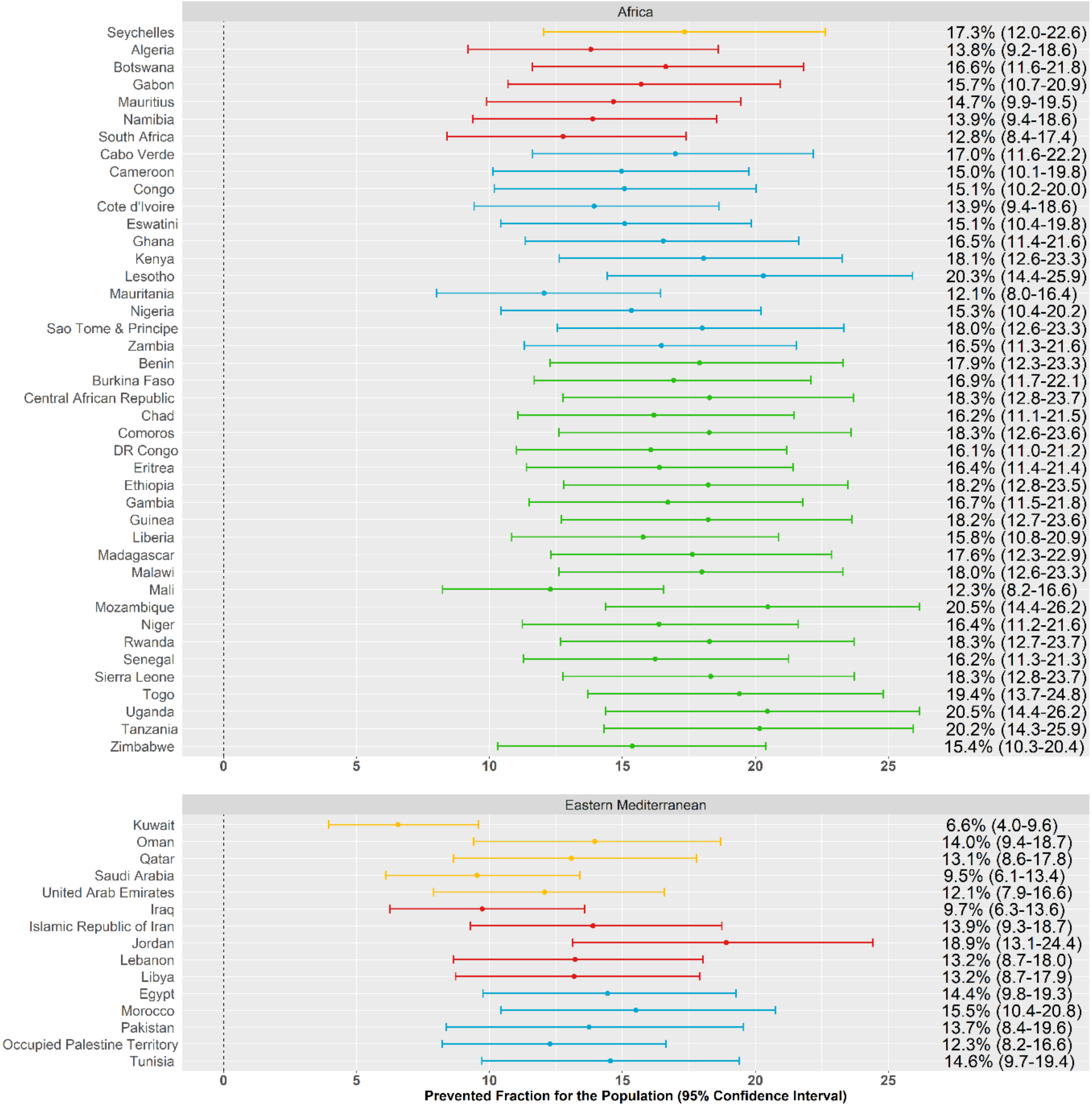

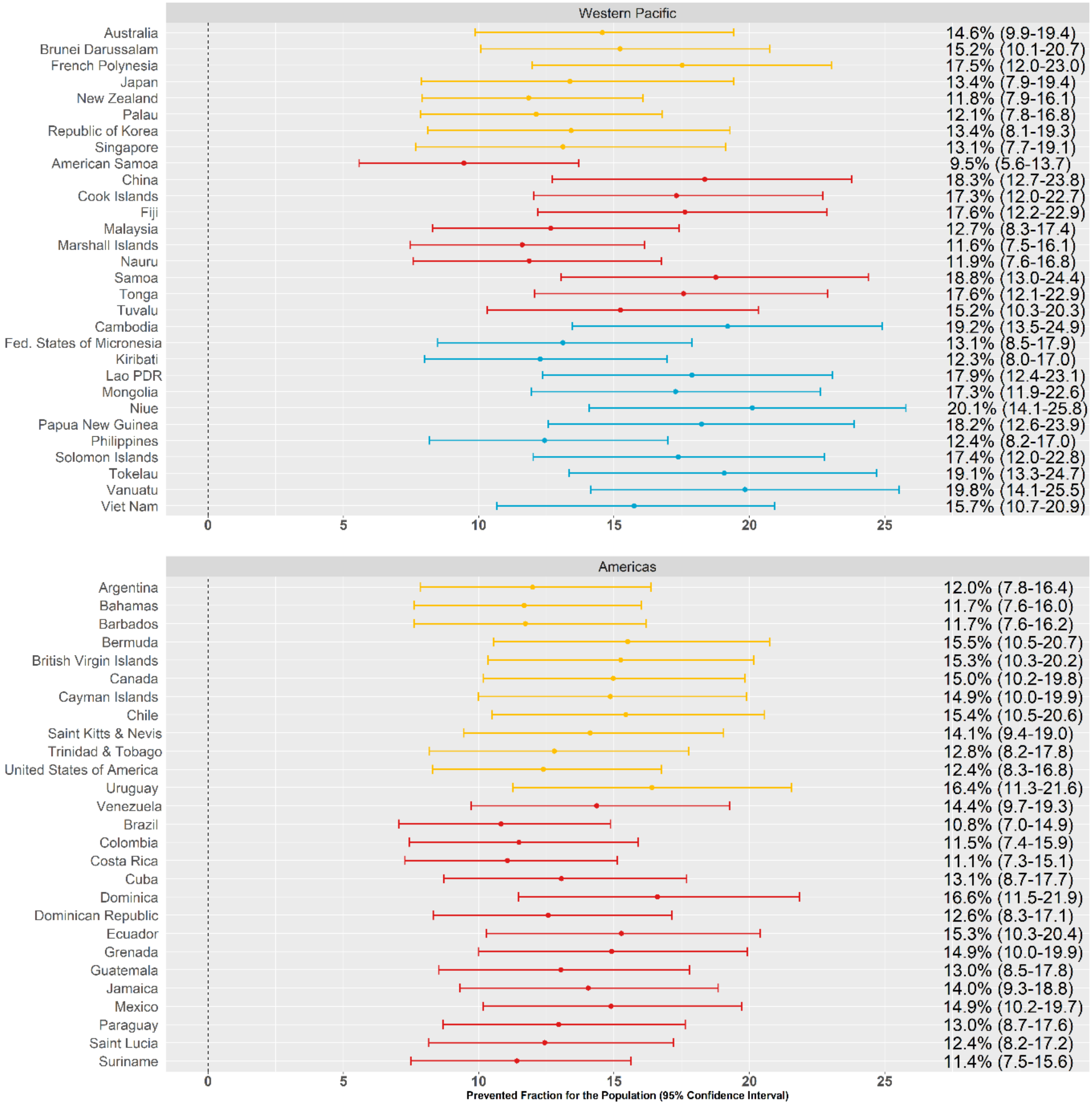
Western Pacific
Australia 14.6% (9.9-19.4)
Brunei Darussalam 15.2% (10.1-20.7)
French Polynesia 17.5% (12.0-23.0)
Japan 13.4% (7.9-19.4)
New Zealand 11.8% (7.9-16.1)
Palau 12.1% (7.8-16.8)
Republic of Korea 13.4% (8.1-19.3)
Singapore 13.1% (7.7-19.1)
American Samoa 9.5% (5.6-13.7)
China 18.3% (12.7-23.8)
Cook Islands 17.3% (12.0-22.7)
Fiji 17.6% (12.2-22.9)
Malaysia 12.7% (8.3-17.4)
Marshall Islands 11.6% (7.5-16.1)
Nauru 11.9% (7.6-16.8)
Samoa 18.8% (13.0-24.4)
Tonga 17.6% (12.1-22.9)
Tuvalu 15.2% (10.3-20.3)
Cambodia 19.2% (13.5-24.9)
Fed. States of Micronesia 13.1% (8.5-17.9)
Kiribati 12.3% (8.0-17.0)
Lao PDR 17.9% (12.4-23.1)
Mongolia 17.3% (11.9-22.6)
Niue 20.1% (14.1-25.8)
Papua New Guinea 18.2% (12.6-23.9)
Philippines 12.4% (8.2-17.0)
Solomon Islands 17.4% (12.0-22.8)
Tokelau 19.1% (13.3-24.7)
Vanuatu 19.8% (14.1-25.5)
Viet Nam 15.7% (10.7-20.9)
0 5 10 15 20 25
Americas
Argentina 12.0% (7.8-16.4)
Bahamas 11.7% (7.6-16.0)
Barbados 11.7% (7.6-16.2)
Bermuda 15.5% (10.5-20.7)
British Virgin Islands 15.3% (10.3-20.2)
Canada 15.0% (10.2-19.8)
Cayman Islands 14.9% (10.0-19.9)
Chile 15.4% (10.5-20.6)
Saint Kitts & Nevis 14.1% (9.4-19.0)
Trinidad & Tobago 12.8% (8.2-17.8)
United States of America 12.4% (8.3-16.8)
Uruguay 16.4% (11.3-21.6)
Venezuela 14.4% (9.7-19.3)
Brazil 10.8% (7.0-14.9)
Colombia 11.5% (7.4-15.9)
Costa Rica 11.1% (7.3-15.1)
Cuba 13.1% (8.7-17.7)
Dominica 16.6% (11.5-21.9)
Dominican Republic 12.6% (8.3-17.1)
Ecuador 15.3% (10.3-20.4)
Grenada 14.9% (10.0-19.9)
Guatemala 13.0% (8.5-17.8)
Jamaica 14.0% (9.3-18.8)
Mexico 14.9% (10.2-19.7)
Paraguay 13.0% (8.7-17.6)
Saint Lucia 12.4% (8.2-17.2)
Suriname 11.4% (7.5-15.6)
0 5 10 15 20 25
Prevented Fraction for the Population (95% Confidence Interval)

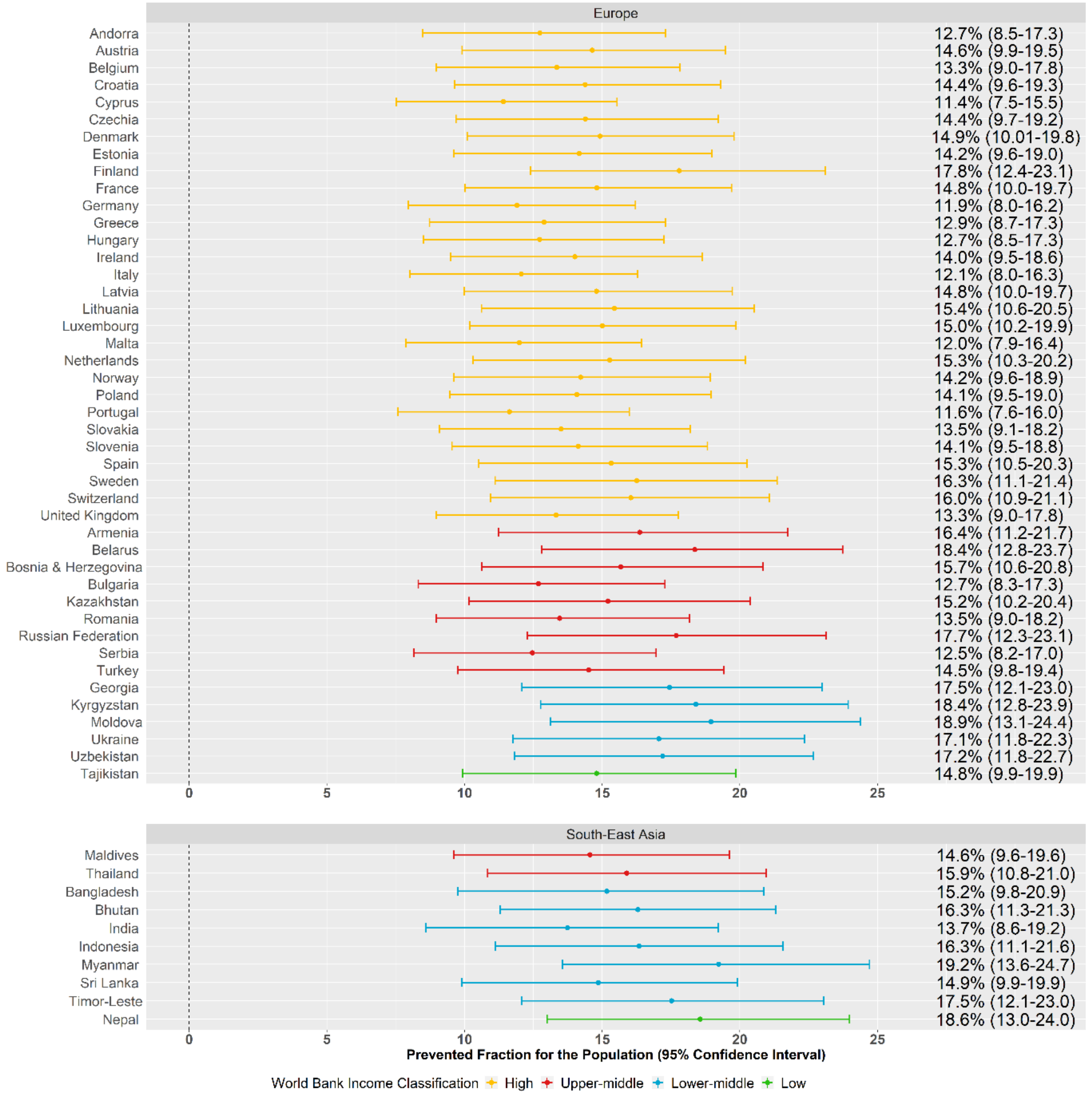
Europe
Andorra 12.7% (8.5-17.3)
Austria 14.6% (9.9-19.5)
Belgium 13.3% (9.0-17.8)
Croatia 14.4% (9.6-19.3)
Cyprus 11.4% (7.5-15.5)
Czechia 14.4% (9.7-19.2)
Denmark 14.9% (10.01-19.8)
Estonia 14.2% (9.6-19.0)
Finland 17.8% (12.4-23.1)
France 14.8% (10.0-19.7)
Germany 11.9% (8.0-16.2)
Greece 12.9% (8.7-17.3)
Hungary 12.7% (8.5-17.3)
Ireland 14.0% (9.5-18.6)
Italy 12.1% (8.0-16.3)
Latvia 14.8% (10.0-19.7)
Lithuania 15.4% (10.6-20.5)
Luxembourg 15.0% (10.2-19.9)
Malta 12.0% (7.9-16.4)
Netherlands 15.3% (10.3-20.2)
Norway 14.2% (9.6-18.9)
Poland 14.1% (9.5-19.0)
Portugal 11.6% (7.6-16.0)
Slovakia 13.5% (9.1-18.2)
Slovenia 14.1% (9.5-18.8)
Spain 15.3% (10.5-20.3)
Sweden 16.3% (11.1-21.4)
Switzerland 16.0% (10.9-21.1)
United Kingdom 13.3% (9.0-17.8)
Armenia 16.4% (11.2-21.7)
Belarus 18.4% (12.8-23.7)
Bosnia & Herzegovina 15.7% (10.6-20.8)
Bulgaria 12.7% (8.3-17.3)
Kazakhstan 15.2% (10.2-20.4)
Romania 13.5% (9.0-18.2)
Russian Federation 17.7% (12.3-23.1)
Serbia 12.5% (8.2-17.0)
Turkey 14.5% (9.8-19.4)
Georgia 17.5% (12.1-23.0)
Kyrgyzstan 18.4% (12.8-23.9)
Moldova 18.9% (13.1-24.4)
Ukraine 17.1% (11.8-22.3)
Uzbekistan 17.2% (11.8-22.7)
Tajikistan 14.8% (9.9-19.9)
0 5 10 15 20 25
South-East Asia
Maldives 14.6% (9.6-19.6)
Thailand 15.9% (10.8-21.0)
Bangladesh 15.2% (9.8-20.9)
Bhutan 16.3% (11.3-21.3)
India 13.7% (8.6-19.2)
Indonesia 16.3% (11.1-21.6)
Myanmar 19.2% (13.6-24.7)
Sri Lanka 14.9% (9.9-19.9)
Timor-Leste 17.5% (12.1-23.0)
Nepal 18.6% (13.0-24.0)
0 5 10 15 20 25
Prevented Fraction for the Population (95% Confidence Interval)
World Bank Income Classification High Upper-middle Lower-middle Low

Appendix 12. The prevalence of activity, the Prevented Fractions for the Population, and the corresponding number of deaths averted for 168 countries.

					Prevented Fractions for the Population (95% confidence intervals)				
World Health Organization region	World Bank income group classification	Country	Prevalence of activity (95% confidence intervals)	Prevalence of activity amongst those that died (95% confidence intervals)	Main adjusted estimate (0% activity counterfactual)[a]	Unadjusted estimate (0% activity counterfactual)[b]	Partially adjusted estimate (0% activity counterfactual)[c]	Adjusted estimate (54.3% active counterfactual)[d]	Number of deaths averted between ages of 40-74 years (thousands)
Africa									
	High								
		Seychelles	81.2 (74.8-86.2)	74.6 (66.9-80.9)	17.3 (12.0-22.6)	26.0 (21.3-30.7)	17.7 (12.5-22.9)	6.9 (4.3-9.7)	0.1
	Upper-middle								
		Algeria	66.4 (57.3-74.5)	57.3 (47.7-66.5)	13.8 (9.2-18.6)	21.2 (16.7-25.9)	14.5 (10.0-19.1)	3.0 (0.6-5.7)	10.6
		Botswana	78.3 (72.5-83.1)	71.0 (64.2-77.0)	16.6 (11.6-21.8)	25.0 (20.4-29.6)	17.0 (12.0-21.9)	6.2 (3.9-8.7)	1.4
		Gabon	74.7 (66.5-81.5)	66.7 (57.4-75.0)	15.7 (10.7-20.9)	23.9 (19.1-28.8)	16.3 (11.4-21.3)	5.2 (2.7-8.0)	0.9
		Mauritius	70.2 (62.2-77.2)	61.6 (52.8-69.7)	14.7 (9.9-19.5)	22.4 (18.0-27.1)	15.3 (10.6-20.0)	4.0 (1.8-6.5)	0.9
		Namibia	66.6 (58.9-73.6)	57.5 (49.3-65.5)	13.9 (9.4-18.6)	21.3 (17.0-25.7)	14.5 (10.1-19.1)	3.1 (1.0-5.5)	1.3
		South Africa	61.8 (53.2-69.9)	52.4 (43.6-61.2)	12.8 (8.4-17.4)	19.8 (15.6-24.3)	13.5 (9.4-17.8)	1.9 (-0.3-4.2)	42.7
	Lower-middle								
		Cabo Verde	80.3 (74.7-85.0)	73.5 (66.7-79.4)	17.0 (11.6-22.2)	25.7 (21.1-30.2)	17.5 (12.3-22.5)	6.7 (4.3-9.2)	0.2
		Cameroon	71.5 (64.9-77.3)	63.0 (55.7-69.8)	15.0 (10.1-19.8)	22.8 (18.5-27.2)	15.6 (10.9-20.3)	4.4 (2.3-6.7)	11.1
		Congo	72.0 (64.5-78.4)	63.6 (55.3-71.2)	15.1 (10.2-20.0)	23.0 (18.6-27.6)	15.7 (11.0-20.5)	4.5 (2.3-7.0)	2.2
		Côte d'Ivoire	66.9 (60.6-73.3)	57.9 (51.1-65.1)	13.9 (9.4-18.6)	21.4 (17.4-25.6)	14.6 (10.2-18.9)	3.2 (1.3-5.3)	14.2
		Eswatini	72.0 (67.1-76.9)	63.6 (58.1-69.4)	15.1 (10.4-19.8)	23.0 (18.9-27.1)	15.7 (11.0-20.2)	4.5 (2.7-6.5)	0.8
		Ghana	78.2 (73.7-82.7)	70.9 (65.6-76.5)	16.5 (11.4-21.6)	25.0 (20.5-29.3)	17.1 (12.1-21.9)	6.1 (4.0-8.4)	14.8
		Kenya	84.6 (82.2-86.9)	78.9 (75.8-81.8)	18.1 (12.6-23.3)	27.0 (22.3-31.5)	18.5 (13.1-23.6)	7.8 (5.4-10.2)	18.0
		Lesotho	93.7 (91.4-95.5)	91.0 (87.8-93.5)	20.3 (14.4-25.9)	29.9 (24.6-34.9)	20.5 (14.5-26.1)	10.3 (7.2-13.3)	2.7
		Mauritania	58.7 (50.8-66.6)	49.1 (41.2-57.5)	12.1 (8.0-16.4)	18.8 (14.7-22.9)	12.8 (8.9-16.9)	1.1 (-1.0-3.3)	1.2

		Nigeria	72.9 (66.5-78.5)	64.6 (57.4-71.3)	15.3 (10.4-20.2)	23.3 (19.0-27.7)	15.9 (11.2-20.6)	4.7 (2.7-7.1)	110.6
		São Tomé and Príncipe	84.5 (79.8-88.3)	78.7 (72.9-83.7)	18.0 (12.6-23.3)	27.0 (22.3-31.7)	18.4 (13.2-23.6)	7.8 (5.3-10.5)	0.1
		Zambia	77.9 (73.2-82.7)	70.6 (65.0-76.5)	16.5 (11.3-21.6)	24.9 (20.4-29.3)	17.0 (12.0-21.9)	6.0 (3.9-8.4)	6.3
	Low								
		Benin	84.1 (79.4-87.9)	78.2 (72.4-83.2)	17.9 (12.3-23.3)	26.9 (22.0-31.6)	18.3 (12.9-23.5)	7.7 (5.1-10.4)	5.2
		Burkina Faso	79.7 (74.5-84.9)	72.7 (66.5-79.3)	16.9 (11.7-22.1)	25.5 (20.8-30.2)	17.4 (12.3-22.4)	6.5 (4.2-9.1)	8.0
		Central African Republic	85.7 (81.5-89.1)	80.3 (75.0-84.7)	18.3 (12.8-23.7)	27.4 (22.5-32.0)	18.6 (13.2-23.9)	8.1 (5.6-10.8)	4.2
		Chad	76.7 (70.0-83.4)	69.1 (61.3-77.4)	16.2 (11.1-21.5)	24.5 (19.9-29.3)	16.7 (11.7-21.7)	5.7 (3.3-8.5)	6.6
		Comoros	85.7 (82.1-89.3)	80.3 (75.7-85.0)	18.3 (12.6-23.6)	27.4 (22.4-32.0)	18.7 (13.3-23.9)	8.1 (5.5-10.8)	0.4
		Democratic Republic of the Congo	76.2 (70.0-81.4)	68.5 (61.3-74.8)	16.1 (11.0-21.2)	24.4 (19.8-29.0)	16.6 (11.7-21.5)	5.6 (3.4-8.0)	33.1
		Eritrea	77.6 (74.1-81.2)	70.2 (66.0-74.6)	16.4 (11.4-21.4)	24.8 (20.4-29.1)	17.0 (12.0-21.6)	6.0 (4.0-8.1)	2.7
		Ethiopia	85.1 (82.4-87.9)	79.5 (76.1-83.2)	18.2 (12.8-23.5)	27.2 (22.5-31.8)	18.6 (13.2-23.8)	8.0 (5.5-10.4)	46.0
		Gambia	78.9 (72.9-84.0)	71.8 (64.6-78.1)	16.7 (11.5-21.8)	25.2 (20.6-29.8)	17.2 (12.0-22.1)	6.3 (4.0-8.9)	0.8
		Guinea	85.5 (80.9-89.2)	80.0 (74.2-84.9)	18.2 (12.7-23.6)	27.3 (22.4-32.0)	18.6 (13.3-23.8)	8.0 (5.5-10.8)	7.7
		Liberia	74.9 (68.7-80.2)	67.0 (59.9-73.4)	15.8 (10.8-20.9)	24.0 (19.5-28.4)	16.3 (11.4-21.1)	5.2 (3.1-7.6)	2.2
		Madagascar	82.8 (78.0-86.8)	76.6 (70.7-81.7)	17.6 (12.3-22.9)	26.5 (21.8-31.0)	18.0 (12.7-23.1)	7.4 (4.9-9.9)	11.2
		Malawi	84.4 (82.1-86.7)	78.6 (75.7-81.6)	18.0 (12.6-23.3)	26.9 (22.3-31.4)	18.4 (13.0-23.5)	7.8 (5.4-10.1)	8.3
		Mali	59.6 (52.7-66.4)	50.1 (43.1-57.3)	12.3 (8.2-16.6)	19.1 (15.2-23.0)	13.0 (9.1-16.9)	1.3 (-0.5-3.3)	5.4
		Mozambique	94.4 (92.4-95.9)	92.0 (89.2-94.1)	20.5 (14.4-26.2)	30.2 (25.0-35.0)	20.6 (14.6-26.3)	10.5 (7.4-13.5)	19.9
		Niger	77.6 (70.4-83.5)	70.2 (61.8-77.5)	16.4 (11.2-21.6)	24.8 (20.1-29.5)	16.9 (12.0-21.9)	6.0 (3.6-8.8)	8.3
		Rwanda	85.4 (81.1-88.9)	79.9 (74.5-84.5)	18.3 (12.7-23.7)	27.3 (22.4-31.9)	18.6 (13.1-23.9)	8.1 (5.5-10.7)	4.8
		Senegal	76.9 (72.5-81.3)	69.4 (64.2-74.7)	16.2 (11.3-21.3)	24.6 (20.1-28.8)	16.8 (11.8-21.4)	5.8 (3.7-8.0)	5.5
		Sierra Leone	85.7 (81.3-89.2)	80.3 (74.7-84.9)	18.3 (12.8-23.7)	27.4 (22.4-32.0)	18.8 (13.4-24.0)	8.1 (5.5-10.8)	6.0
		Togo	90.2 (87.2-92.6)	86.2 (82.2-89.5)	19.4 (13.7-24.8)	28.8 (23.9-33.6)	19.7 (13.9-25.1)	9.3 (6.5-12.3)	4.7
		Uganda	94.5 (92.4-96.0)	92.1 (89.2-94.2)	20.5 (14.4-26.2)	30.2 (25.0-35.3)	20.6 (14.7-26.4)	10.5 (7.4-13.5)	19.2
		United Republic of Tanzania	93.5 (91.4-95.2)	90.7 (87.8-93.1)	20.2 (14.3-25.9)	29.9 (24.8-34.8)	20.4 (14.5-26.0)	10.3 (7.2-13.1)	29.0
		Zimbabwe	73.2 (65.7-79.5)	65.0 (56.6-72.5)	15.4 (10.3-20.4)	23.4 (18.8-28.0)	16.0 (11.2-20.7)	4.8 (2.5-7.3)	7.9
Americas									

	High								
		Argentina	58.4 (50.0-66.4)	48.8 (40.5-57.3)	12.0 (7.8-16.4)	18.6 (14.6-22.8)	12.7 (8.8-16.8)	1.0 (-1.2-3.2)	17.5
		Bahamas	56.7 (48.1-65.1)	47.1 (38.7-55.9)	11.7 (7.6-16.0)	18.1 (14.0-22.4)	12.4 (8.5-16.5)	0.6 (-1.7-2.8)	0.2
		Barbados	57.1 (48.3-65.9)	47.5 (38.8-56.8)	11.7 (7.6-16.2)	18.2 (14.1-22.6)	12.5 (8.5-16.6)	0.7 (-1.7-3.1)	0.1
		Bermuda	73.8 (65.4-80.8)	65.7 (56.2-74.1)	15.5 (10.5-20.7)	23.6 (18.9-28.4)	16.2 (11.3-20.9)	5.0 (2.4-7.8)	0.0[e]
		British Virgin Islands	72.6 (65.1-79.1)	64.3 (55.9-72.0)	15.3 (10.3-20.2)	23.2 (18.6-27.9)	15.8 (11.1-20.7)	4.6 (2.4-7.1)	0.0[e]
		Canada	71.4 (63.7-78.1)	62.9 (54.4-70.8)	15.0 (10.2-19.8)	22.9 (18.4-27.5)	15.6 (10.8-20.5)	4.3 (2.1-6.9)	15.0
		Cayman Islands	70.9 (62.5-78.1)	62.4 (53.1-70.8)	14.9 (10.0-19.9)	22.7 (18.1-27.4)	15.5 (10.7-20.2)	4.2 (1.9-6.9)	0.0[e]
		Chile	73.4 (66.4-80.4)	65.2 (57.3-73.6)	15.4 (10.5-20.6)	23.5 (19.0-28.1)	16.0 (11.2-20.7)	4.9 (2.5-7.5)	8.4
		Saint Kitts and Nevis	67.8 (58.8-75.7)	58.9 (49.2-67.9)	14.1 (9.4-19.0)	21.7 (17.1-26.4)	14.8 (10.2-19.4)	3.4 (1.0-6.0)	0.0[e]
		Trinidad and Tobago	61.8 (50.4-72.2)	52.4 (40.9-63.8)	12.8 (8.2-17.8)	19.8 (14.9-24.8)	13.5 (9.1-18.1)	1.9 (-1.2-4.9)	1.0
		United States of America	60.0 (52.7-67.0)	50.5 (43.1-58.0)	12.4 (8.3-16.8)	19.2 (15.3-23.3)	13.1 (9.1-17.2)	1.4 (-0.4-3.4)	140.2
		Uruguay	77.6 (71.0-83.1)	70.2 (62.5-77.0)	16.4 (11.3-21.6)	24.8 (20.2-29.5)	17.0 (12.0-22.0)	6.0 (3.6-8.6)	2.3
	Upper-middle								
		Bolivarian Republic of Venezuela	68.6 (60.0-76.0)	59.8 (50.5-68.3)	14.4 (9.7-19.3)	22.0 (17.6-26.6)	14.9 (10.4-19.6)	3.6 (1.3-6.3)	13.2
		Brazil	53.0 (44.7-61.1)	43.4 (35.5-51.6)	10.8 (7.0-14.9)	16.9 (13.1-20.9)	11.6 (8.0-15.5)	-0.3 (-2.5-1.8)	71.6
		Colombia	56.0 (47.3-64.6)	46.4 (37.9-55.4)	11.5 (7.4-15.9)	17.9 (13.9-22.1)	12.2 (8.3-16.3)	0.4 (-2.0-2.8)	15.5
		Costa Rica	53.9 (45.6-62.1)	44.3 (36.3-52.7)	11.1 (7.3-15.1)	17.2 (13.5-21.3)	11.8 (8.0-15.7)	-0.1 (-2.3-2.0)	1.2
		Cuba	63.1 (54.8-70.8)	53.8 (45.2-62.2)	13.1 (8.7-17.7)	20.1 (15.9-24.6)	13.8 (9.6-18.1)	2.2 (0.0-4.5)	5.5
		Dominica	78.4 (72.0-83.7)	71.2 (63.6-77.7)	16.6 (11.5-21.9)	25.1 (20.5-29.7)	17.1 (12.1-22.1)	6.2 (3.8-8.8)	0.0[e]
		Dominican Republic	61.0 (52.3-69.1)	51.5 (42.7-60.3)	12.6 (8.3-17.1)	19.5 (15.3-24.0)	13.3 (9.1-17.6)	1.7 (-0.6-4.0)	3.8
		Ecuador	72.8 (65.4-80.3)	64.5 (56.2-73.5)	15.3 (10.3-20.4)	23.2 (18.7-28.1)	15.9 (11.1-20.7)	4.7 (2.3-7.4)	5.3
		Grenada	71.3 (62.8-78.6)	62.8 (53.4-71.4)	14.9 (10.0-19.9)	22.8 (18.1-27.5)	15.6 (10.8-20.5)	4.3 (1.9-7.0)	0.1
		Guatemala	62.9 (52.6-72.1)	53.5 (43.0-63.7)	13.0 (8.5-17.8)	20.1 (15.5-24.9)	13.7 (9.4-18.2)	2.2 (-0.6-5.0)	3.7
		Jamaica	67.4 (59.2-74.8)	58.4 (49.7-66.9)	14.0 (9.3-18.8)	21.5 (17.0-26.2)	14.7 (10.3-19.2)	3.3 (1.2-5.7)	1.1
		Mexico	71.1 (64.4-77.0)	62.6 (55.2-69.5)	14.9 (10.2-19.7)	22.7 (18.5-27.1)	15.5 (10.8-20.1)	4.2 (2.2-6.6)	41.3
		Paraguay	62.6 (54.1-71.1)	53.2 (44.5-62.6)	13.0 (8.7-17.6)	20.0 (15.7-24.5)	13.6 (9.4-18.0)	2.1 (-0.3-4.6)	2.1
		Saint Lucia	60.2 (50.1-69.7)	50.7 (40.6-61.0)	12.4 (8.2-17.2)	19.3 (14.8-24.1)	13.2 (8.9-17.6)	1.5 (-1.2-4.2)	0.1

		Suriname	55.6 (47.3-63.6)	46.0 (37.9-54.3)	11.4 (7.5-15.6)	17.8 (13.9-21.9)	12.2 (8.4-16.1)	0.3 (-1.9-2.5)	0.2
Eastern Mediterranean									
	High								
		Kuwait	33.0 (25.7-41.4)	25.1 (19.0-32.4)	6.6 (4.0-9.6)	10.5 (7.5-14.0)	7.2 (4.6-10.1)	-5.1 (-8.1--2.7)	0.3
		Oman	67.1 (58.2-74.8)	58.1 (48.6-66.9)	14.0 (9.4-18.7)	21.5 (17.0-26.1)	14.6 (10.1-19.3)	3.2 (0.9-5.7)	0.7
		Qatar	63.2 (53.8-71.6)	53.9 (44.2-63.2)	13.1 (8.6-17.8)	20.2 (15.7-24.8)	13.8 (9.4-18.4)	2.2 (-0.3-4.8)	0.2
		Saudi Arabia	46.9 (38.4-55.5)	37.5 (29.8-45.9)	9.5 (6.1-13.4)	15.0 (11.3-19.0)	10.2 (6.9-13.7)	-1.8 (-4.4-0.4)	5.1
		United Arab Emirates	58.6 (50.1-67.2)	49.0 (40.6-58.2)	12.1 (7.9-16.6)	18.7 (14.6-23.1)	12.8 (8.8-16.9)	1.1 (-1.3-3.5)	1.0
	Upper-middle								
		Iraq	48.0 (40.2-55.9)	38.6 (31.4-46.3)	9.7 (6.3-13.6)	15.3 (11.8-19.1)	10.5 (7.1-14.1)	-1.6 (-3.8-0.5)	7.0
		Islamic Republic of Iran	66.8 (58.6-74.1)	57.8 (49.0-66.0)	13.9 (9.3-18.7)	21.3 (16.9-26.0)	14.6 (10.1-19.2)	3.1 (0.9-5.7)	21.9
		Jordan	88.1 (83.6-91.6)	83.4 (77.6-88.1)	18.9 (13.1-24.4)	28.2 (23.1-33.1)	19.2 (13.7-24.6)	8.8 (5.9-11.7)	3.2
		Lebanon	63.6 (54.6-71.7)	54.3 (45.0-63.3)	13.2 (8.7-18.0)	20.4 (15.9-25.0)	13.9 (9.6-18.4)	2.3 (-0.1-5.0)	1.5
		Libya	63.6 (54.3-72.0)	54.3 (44.7-63.6)	13.2 (8.7-17.9)	20.3 (16.0-24.9)	13.9 (9.6-18.3)	2.3 (-0.1-4.9)	2.0
	Lower-middle								
		Egypt	69.0 (60.5-76.4)	60.2 (51.0-68.8)	14.4 (9.8-19.3)	22.1 (17.6-26.7)	15.0 (10.5-19.7)	3.7 (1.4-6.4)	44.6
		Morocco	73.8 (64.6-81.4)	65.7 (55.4-74.8)	15.5 (10.4-20.8)	23.6 (18.7-28.7)	16.1 (11.2-21.2)	5.0 (2.3-8.0)	10.9
		Pakistan	66.3 (53.0-79.7)	57.2 (43.4-72.7)	13.7 (8.4-19.6)	21.2 (15.4-27.3)	14.5 (9.4-19.9)	3.0 (-1.1-7.1)	70.9
		Occupied Palestine Territory	59.5 (51.0-67.5)	50.0 (41.4-58.5)	12.3 (8.2-16.6)	19.0 (14.8-23.3)	13.0 (8.9-17.2)	1.3 (-0.8-3.6)	0.9
		Tunisia	69.6 (61.3-76.9)	60.9 (51.8-69.4)	14.6 (9.7-19.4)	22.2 (17.7-26.9)	15.2 (10.5-19.9)	3.9 (1.5-6.4)	4.9
Europe									
	High								
		Andorra	61.6 (53.4-69.2)	52.2 (43.8-60.4)	12.7 (8.5-17.3)	19.7 (15.5-24.0)	13.5 (9.2-17.7)	1.8 (-0.3-4.1)	0.0[e]
		Austria	69.9 (63.2-75.9)	61.2 (53.9-68.2)	14.6 (9.9-19.5)	22.3 (18.1-26.7)	15.3 (10.8-19.8)	4.0 (2.0-6.2)	4.3
		Belgium	64.3 (57.2-70.7)	55.0 (47.6-62.1)	13.3 (9.0-17.8)	20.6 (16.4-24.8)	14.0 (9.8-18.4)	2.5 (0.6-4.6)	4.9
		Croatia	68.9 (61.0-75.9)	60.1 (51.5-68.2)	14.4 (9.6-19.3)	22.0 (17.5-26.6)	15.0 (10.4-19.7)	3.7 (1.5-6.2)	3.3
		Cyprus	55.6 (47.9-63.2)	46.0 (38.5-53.9)	11.4 (7.5-15.5)	17.8 (13.9-21.9)	12.1 (8.3-16.0)	0.3 (-1.8-2.5)	0.3

		Czechia	68.9 (61.2-75.8)	60.1 (51.7-68.0)	14.4 (9.7-19.2)	22.0 (17.7-26.6)	15.0 (10.5-19.6)	3.7 (1.6-6.1)	7.3
		Denmark	71.5 (65.0-77.3)	63.0 (55.8-69.8)	14.9 (10.1-19.8)	22.8 (18.4-27.2)	15.6 (10.9-20.3)	4.3 (2.3-6.7)	3.3
		Estonia	68.0 (60.2-74.9)	59.1 (50.7-67.0)	14.2 (9.6-19.0)	21.7 (17.3-26.2)	14.8 (10.4-19.3)	3.4 (1.3-5.9)	1.0
		Finland	83.4 (79.0-87.1)	77.4 (71.9-82.1)	17.8 (12.4-23.1)	26.7 (21.9-31.3)	18.2 (12.8-23.3)	7.5 (5.0-10.1)	3.7
		France	70.7 (64.1-76.5)	62.1 (54.8-68.9)	14.8 (10.0-19.7)	22.6 (18.2-27.0)	15.4 (10.8-19.9)	4.2 (2.2-6.5)	28.5
		Germany	57.8 (50.8-64.5)	48.2 (41.2-55.3)	11.9 (8.0-16.2)	18.5 (14.7-22.5)	12.6 (8.7-16.5)	0.9 (-1.0-2.7)	37.4
		Greece	62.3 (55.1-69.1)	52.9 (45.5-60.3)	12.9 (8.7-17.3)	19.9 (16.0-24.0)	13.6 (9.5-17.8)	2.0 (0.1-4.1)	4.6
		Hungary	61.5 (53.2-69.1)	52.1 (43.6-60.3)	12.7 (8.5-17.3)	19.7 (15.6-24.0)	13.4 (9.3-17.7)	1.8 (-0.3-4.1)	8.2
		Ireland	67.3 (60.5-73.5)	58.3 (51.0-65.3)	14.0 (9.5-18.6)	21.5 (17.3-25.7)	14.7 (10.3-19.1)	3.3 (1.4-5.4)	1.7
		Italy	58.6 (51.2-65.7)	49.0 (41.6-56.6)	12.1 (8.0-16.3)	18.7 (14.9-22.7)	12.8 (9.0-16.8)	1.1 (-0.8-3.0)	21.0
		Latvia	70.5 (62.7-77.2)	61.9 (53.3-69.7)	14.8 (10.0-19.7)	22.6 (18.1-27.1)	15.4 (10.9-20.1)	4.1 (1.9-6.6)	2.2
		Lithuania	73.5 (66.4-79.5)	65.3 (57.3-72.5)	15.4 (10.6-20.5)	23.5 (19.0-28.0)	16.1 (11.1-20.8)	4.9 (2.7-7.4)	3.2
		Luxembourg	71.6 (65.1-77.3)	63.2 (55.9-69.8)	15.0 (10.2-19.9)	22.9 (18.5-27.2)	15.6 (11.0-20.1)	4.4 (2.4-6.7)	0.2
		Malta	58.3 (49.7-66.4)	48.7 (40.2-57.3)	12.0 (7.9-16.4)	18.7 (14.6-23.0)	12.7 (8.8-16.9)	1.0 (-1.2-3.2)	0.2
		Netherlands	72.8 (66.5-78.4)	64.5 (57.4-71.2)	15.3 (10.3-20.2)	23.3 (18.8-27.7)	15.9 (11.1-20.6)	4.7 (2.6-7.1)	8.1
		Norway	68.3 (61.3-74.6)	59.4 (51.8-66.6)	14.2 (9.6-18.9)	21.8 (17.5-26.2)	14.9 (10.3-19.3)	3.5 (1.6-5.7)	1.9
		Poland	67.5 (59.5-74.7)	58.5 (50.0-66.7)	14.1 (9.5-19.0)	21.6 (17.3-26.2)	14.7 (10.2-19.2)	3.3 (1.2-5.8)	27.0
		Portugal	56.6 (47.8-65.0)	47.0 (38.4-55.8)	11.6 (7.6-16.0)	18.1 (14.1-22.5)	12.4 (8.4-16.5)	0.6 (-1.7-2.9)	4.2
		Slovakia	65.1 (57.0-72.4)	55.9 (47.4-64.1)	13.5 (9.1-18.2)	20.8 (16.5-25.3)	14.2 (9.8-18.7)	2.7 (0.6-5.0)	3.6
		Slovenia	67.8 (59.9-74.8)	58.9 (50.4-66.9)	14.1 (9.5-18.8)	21.7 (17.3-26.1)	14.8 (10.3-19.4)	3.4 (1.3-5.9)	1.1
		Spain	73.2 (66.8-78.8)	65.0 (57.8-71.6)	15.3 (10.5-20.3)	23.4 (19.0-27.7)	16.0 (11.2-20.7)	4.8 (2.8-7.1)	19.3
		Sweden	76.9 (71.1-81.8)	69.4 (62.6-75.3)	16.3 (11.1-21.4)	24.6 (19.9-29.1)	16.8 (11.8-21.6)	5.8 (3.6-8.2)	4.6
		Switzerland	76.3 (69.7-81.8)	68.6 (61.0-75.3)	16.0 (10.9-21.1)	24.3 (19.7-28.8)	16.7 (11.6-21.5)	5.6 (3.3-8.1)	3.4
		United Kingdom	64.1 (57.1-70.6)	54.8 (47.5-62.0)	13.3 (9.0-17.8)	20.5 (16.5-24.7)	14.0 (9.8-18.2)	2.4 (0.6-4.5)	26.6
	Upper-middle								
		Armenia	77.4 (69.5-83.6)	70.0 (60.8-77.6)	16.4 (11.2-21.7)	24.8 (20.0-29.8)	16.9 (11.8-21.9)	5.9 (3.4-8.8)	2.4
		Belarus	85.9 (80.5-90.0)	80.5 (73.7-86.0)	18.4 (12.8-23.7)	27.5 (22.6-32.3)	18.7 (13.2-24.0)	8.2 (5.4-11.1)	13.4
		Bosnia and Herzegovina	74.5 (66.1-81.5)	66.5 (57.0-75.0)	15.7 (10.6-20.8)	23.8 (19.1-28.8)	16.3 (11.4-21.3)	5.1 (2.6-8.0)	3.0
		Bulgaria	61.4 (52.7-69.4)	52.0 (43.1-60.7)	12.7 (8.3-17.3)	19.6 (15.3-24.1)	13.4 (9.3-17.7)	1.8 (-0.4-4.2)	6.4

		Kazakhstan	72.5 (63.2-80.2)	64.2 (53.9-73.4)	15.2 (10.2-20.4)	23.2 (18.2-28.2)	15.8 (11.0-20.8)	4.6 (1.9-7.7)	13.8
		Romania	64.6 (56.3-72.2)	55.4 (46.7-63.8)	13.5 (9.0-18.2)	20.7 (16.4-25.2)	14.1 (9.7-18.6)	2.6 (0.4-5.0)	16.3
		Russian Federation	82.9 (78.2-87.6)	76.7 (70.9-82.8)	17.7 (12.3-23.1)	26.5 (21.8-31.3)	18.1 (12.9-23.1)	7.4 (4.9-10.1)	197.2
		Serbia	60.5 (51.2-69.2)	51.0 (41.6-60.4)	12.5 (8.2-17.0)	19.3 (15.0-23.9)	13.3 (9.0-17.6)	1.5 (-0.8-4.1)	6.6
		Turkey	69.4 (61.2-76.7)	60.7 (51.7-69.1)	14.5 (9.8-19.4)	22.2 (17.6-26.8)	15.1 (10.6-19.8)	3.8 (1.5-6.4)	28.8
	Lower-middle								
		Georgia	82.0 (75.4-87.2)	75.6 (67.6-82.2)	17.5 (12.1-23.0)	26.2 (21.4-31.2)	17.9 (12.5-23.1)	7.1 (4.5-10.1)	4.6
		Kyrgyzstan	86.1 (79.5-90.9)	80.8 (72.5-87.2)	18.4 (12.8-23.9)	27.5 (22.5-32.5)	18.8 (13.3-24.2)	8.2 (5.3-11.3)	3.6
		Republic of Moldova	88.5 (84.0-91.9)	84.0 (78.1-88.5)	18.9 (13.1-24.4)	28.3 (23.2-33.1)	19.3 (13.7-24.8)	8.9 (6.0-11.7)	5.5
		Ukraine	80.4 (73.6-85.8)	73.6 (65.5-80.4)	17.1 (11.8-22.3)	25.7 (20.9-30.5)	17.6 (12.5-22.8)	6.7 (4.1-9.5)	64.0
		Uzbekistan	80.9 (73.3-86.8)	74.2 (65.1-81.7)	17.2 (11.8-22.7)	25.9 (20.9-30.9)	17.7 (12.4-22.9)	6.8 (4.1-9.9)	16.1
	Low								
		Tajikistan	70.7 (61.2-78.8)	62.1 (51.7-71.6)	14.8 (9.9-19.9)	22.6 (17.9-27.5)	15.4 (10.7-20.4)	4.1 (1.6-7.0)	2.8
South-East Asia									
	Upper-middle								
		Maldives	69.7 (59.9-78.0)	61.0 (50.4-70.7)	14.6 (9.6-19.6)	22.3 (17.4-27.3)	15.2 (10.4-20.3)	3.9 (1.2-6.9)	0.1
		Thailand	75.4 (67.7-81.8)	67.6 (58.8-75.3)	15.9 (10.8-21.0)	24.1 (19.4-28.8)	16.5 (11.6-21.4)	5.4 (3.0-8.1)	47.1
	Lower-middle								
		Bangladesh	72.2 (60.9-83.6)	63.8 (51.4-77.6)	15.2 (9.8-20.9)	23.1 (17.7-28.9)	15.8 (10.7-21.2)	4.5 (0.9-8.4)	70.0
		Bhutan	77.0 (72.5-81.6)	69.5 (64.2-75.1)	16.3 (11.3-21.3)	24.6 (20.2-29.0)	16.8 (11.9-21.4)	5.8 (3.8-8.0)	0.4
		India	66.0 (52.3-77.7)	56.9 (42.7-70.3)	13.7 (8.6-19.2)	21.2 (15.4-27.1)	14.4 (9.4-19.7)	2.9 (-0.8-6.7)	708.0
		Indonesia	77.4 (69.1-84.0)	70.0 (60.3-78.1)	16.3 (11.1-21.6)	24.8 (19.8-29.8)	16.9 (11.8-22.0)	5.9 (3.3-8.8)	177.8
		Myanmar	89.3 (85.0-92.5)	85.0 (79.4-89.3)	19.2 (13.6-24.7)	28.6 (23.6-33.4)	19.5 (13.8-25.0)	9.1 (6.3-12.0)	45.9
		Sri Lanka	71.1 (62.1-78.7)	62.6 (52.7-71.5)	14.9 (9.9-19.9)	22.7 (18.0-27.7)	15.5 (10.7-20.3)	4.2 (1.7-7.0)	12.1
		Timor-Leste	82.2 (75.0-87.7)	75.8 (67.1-82.9)	17.5 (12.1-23.0)	26.3 (21.4-31.2)	18.0 (12.6-23.1)	7.2 (4.5-10.2)	0.5
	Low								
		Nepal	86.6 (84.4-88.8)	81.5 (78.6-84.3)	18.6 (13.0-24.0)	27.7 (22.9-32.3)	18.9 (13.6-24.2)	8.4 (5.8-10.9)	19.2
Western Pacific									

	High								
		Australia	69.6 (62.1-76.3)	60.9 (52.7-68.6)	14.6 (9.9-19.4)	22.3 (17.9-26.8)	15.2 (10.6-19.8)	3.9 (1.7-6.3)	7.7
		Brunei Darussalam	72.7 (61.7-81.5)	64.4 (52.3-75.0)	15.2 (10.1-20.7)	23.2 (18.1-28.6)	15.8 (10.9-21.1)	4.7 (1.6-8.0)	0.1
		French Polynesia	82.1 (75.6-87.2)	75.7 (67.8-82.2)	17.5 (12.0-23.0)	26.3 (21.3-31.2)	17.9 (12.6-23.1)	7.2 (4.5-10.0)	0.2
		Japan	64.5 (46.2-79.5)	55.3 (36.9-72.5)	13.4 (7.9-19.4)	20.6 (14.1-27.5)	14.0 (8.8-19.9)	2.5 (-2.1-7.2)	53.0
		New Zealand	57.6 (50.2-64.7)	48.0 (40.7-55.5)	11.8 (7.9-16.1)	18.4 (14.6-22.5)	12.5 (8.8-16.5)	0.8 (-1.1-2.7)	1.3
		Palau	59.1 (48.7-68.9)	49.6 (39.2-60.1)	12.1 (7.8-16.8)	18.9 (14.3-23.6)	12.9 (8.7-17.3)	1.2 (-1.6-3.9)	0.0[e]
		Republic of Korea	64.6 (47.1-79.1)	55.4 (37.7-72.0)	13.4 (8.1-19.3)	20.7 (14.0-27.6)	14.1 (8.7-20.1)	2.6 (-2.0-7.0)	17.7
		Singapore	63.5 (45.7-78.3)	54.2 (36.4-71.0)	13.1 (7.7-19.1)	20.3 (13.6-27.4)	13.9 (8.5-19.8)	2.2 (-2.5-6.8)	1.6
	Upper-middle								
		American Samoa	46.6 (35.0-58.6)	37.2 (26.8-49.0)	9.5 (5.6-13.7)	14.9 (10.5-19.7)	10.2 (6.5-14.4)	-1.9 (-5.3-1.1)	0.0[e]
		China	85.9 (80.6-89.9)	80.5 (73.9-85.8)	18.3 (12.7-23.8)	27.4 (22.5-32.2)	18.8 (13.2-24.1)	8.2 (5.5-11.0)	1016.5
		Cook Islands	81.5 (75.1-86.7)	75.0 (67.2-81.6)	17.3 (12.0-22.7)	26.0 (21.2-30.8)	17.8 (12.5-22.9)	7.0 (4.4-9.8)	0.0[e]
		Fiji	82.6 (76.4-87.5)	76.3 (68.8-82.6)	17.6 (12.2-22.9)	26.4 (21.6-31.2)	18.0 (12.7-23.2)	7.3 (4.7-10.1)	0.8
		Malaysia	61.2 (51.4-70.3)	51.7 (41.8-61.7)	12.7 (8.3-17.4)	19.5 (15.1-24.4)	13.4 (9.1-17.8)	1.7 (-0.8-4.4)	10.9
		Marshall Islands	56.5 (46.3-66.2)	46.9 (37.0-57.1)	11.6 (7.5-16.1)	18.1 (13.8-22.7)	12.3 (8.3-16.5)	0.5 (-2.1-3.2)	0.0[e]
		Nauru	57.9 (46.7-68.3)	48.3 (37.3-59.4)	11.9 (7.6-16.8)	18.5 (14.0-23.4)	12.6 (8.5-17.2)	0.9 (-2.1-3.9)	0.0[e]
		Samoa	87.4 (82.3-91.3)	82.5 (76.0-87.7)	18.8 (13.0-24.4)	28.0 (22.9-32.9)	19.1 (13.4-24.6)	8.6 (5.8-11.5)	0.1
		Tonga	82.6 (76.4-87.6)	76.3 (68.8-82.8)	17.6 (12.1-22.9)	26.4 (21.5-31.3)	18.0 (12.7-23.2)	7.3 (4.7-10.2)	0.1
		Tuvalu	72.7 (64.6-79.5)	64.4 (55.4-72.5)	15.2 (10.3-20.3)	23.2 (18.7-28.0)	15.8 (11.0-20.7)	4.6 (2.3-7.4)	0.0[e]
	Lower-middle								
		Cambodia	89.5 (84.3-93.1)	85.3 (78.5-90.2)	19.2 (13.5-24.9)	28.6 (23.5-33.6)	19.5 (13.9-25.0)	9.1 (6.2-12.2)	9.9
		Federated States of Micronesia	63.4 (54.0-71.8)	54.1 (44.4-63.4)	13.1 (8.5-17.9)	20.2 (15.6-25.0)	13.8 (9.5-18.3)	2.3 (-0.2-4.9)	0.0
		Kiribati	59.6 (49.8-68.8)	50.1 (40.3-60.0)	12.3 (8.0-17.0)	19.0 (14.6-23.7)	13.0 (8.9-17.4)	1.3 (-1.3-4.0)	0.0
		Lao People's Democratic Republic	83.7 (79.7-87.7)	77.7 (72.7-82.9)	17.9 (12.4-23.1)	26.8 (22.0-31.2)	18.3 (12.9-23.3)	7.6 (5.1-10.1)	3.9
		Mongolia	81.4 (75.6-86.1)	74.8 (67.8-80.8)	17.3 (11.9-22.6)	26.0 (21.3-30.7)	17.8 (12.7-22.7)	7.0 (4.5-9.6)	2.1
		Niue	93.1 (90.1-95.2)	90.2 (86.1-93.01)	20.1 (14.1-25.8)	29.8 (24.5-34.7)	20.3 (14.4-25.9)	10.1 (7.0-13.1)	0.0[e]
		Papua New Guinea	85.2 (78.1-90.3)	79.6 (70.8-86.4)	18.2 (12.6-23.9)	27.3 (22.1-32.4)	18.6 (13.2-24.0)	8.0 (5.1-11.1)	5.2

		Philippines	60.3 (51.4-68.7)	50.8 (41.8-59.9)	12.4 (8.2-17.0)	19.3 (14.9-23.8)	13.2 (9.1-17.6)	1.5 (-0.9-3.9)	46.2
		Solomon Islands	81.8 (75.2-87.0)	75.3 (67.3-82.0)	17.4 (12.0-22.8)	26.1 (21.3-31.0)	17.9 (12.6-23.1)	7.1 (4.5-9.8)	0.2
		Tokelau	88.9 (83.9-92.5)	84.5 (78.0-89.3)	19.1 (13.3-24.7)	28.4 (23.4-33.4)	19.4 (13.7-25.0)	9.0 (6.1-12.0)	0.0[e]
		Vanuatu	92.0 (88.4-94.6)	88.7 (83.8-92.3)	19.8 (14.1-25.5)	29.4 (24.4-34.3)	20.1 (14.3-25.6)	9.8 (6.9-12.8)	0.1
		Viet Nam	74.6 (66.9-81.1)	66.6 (57.9-74.5)	15.7 (10.7-20.9)	23.9 (19.2-28.7)	16.3 (11.5-21.3)	5.2 (2.7-7.9)	39.6

Number of deaths averted for males and females may not add up to the estimate for both sexes. This is because the distributions of deaths across males and females are not even, and differences are magnified when multiplied by a derivative of activity prevalence which also varies considerably by sex.

a: The main estimates calculated using Formula 3 (see Box 1), the adjusted relative risk, and 0% activity counterfactual.

b: The unadjusted estimates calculated using Formula 2 (see Box 1), the unadjusted relative risk, and 0% activity counterfactual.

c: The partially adjusted estimates calculated using Formula 2 (see Box 1), the adjusted relative risk, and 0% activity counterfactual.

d: The adjusted estimates calculated using Formula 5 (see Appendix 10, pp23-25), the adjusted relative risk, and 54.3% activity counterfactual.

e: Deaths averted based on an imputed estimate of total deaths between the ages of 40-79 years. This was derived using the median ratio of deaths to total population for countries in the same region/income categories (Appendix 9, p22).

Appendix 13A. The Prevented Fractions for the Population and corresponding number of deaths averted for women, by country, region and income group.

					Prevented Fractions for the Population (95% confidence intervals)				
World Health Organization region	World Bank income group classification	Country	Prevalence of activity (95% confidence intervals)	Prevalence of activity amongst those that died (95% confidence intervals)	Main adjusted estimate (0% activity counterfactual)[a]	Unadjusted estimate (0% activity counterfactual)[b]	Partially adjusted estimate (0% activity counterfactual)[c]	Adjusted estimate (54.3% active counterfactual)[d]	Number of deaths averted between ages of 40-74 years (thousands)
Africa									
	High								
		Seychelles	80.0 (72.8-85.6)	73.1 (64.5-80.2)	17.0 (11.7-22.3)	25.6 (20.7-30.5)	17.5 (12.2-22.6)	8.3 (5.3-11.5)	0.0
	Upper-middle								
		Algeria	59.8 (49.7-69.0)	50.3 (40.2-60.2)	12.4 (8.1-17.0)	19.1 (14.7-23.7)	13.0 (8.8-17.5)	3.1 (0.5-6.0)	4.2
		Botswana	73.7 (66.9-79.5)	65.6 (57.9-72.5)	15.5 (10.7-20.5)	23.6 (19.2-28.2)	16.1 (11.3-20.8)	6.6 (4.1-9.4)	0.5
		Gabon	66.8 (56.9-75.4)	57.8 (47.3-67.6)	13.8 (9.2-18.9)	21.3 (16.7-26.2)	14.6 (10.0-19.3)	4.8 (2.1-7.9)	0.4
		Mauritius	68.2 (59.5-75.7)	59.3 (50.0-67.9)	14.2 (9.6-19.2)	21.8 (17.3-26.5)	14.9 (10.4-19.5)	5.2 (2.7-8.1)	0.3
		Namibia	62.6 (53.9-70.5)	53.2 (44.3-61.9)	13.0 (8.7-17.6)	20.0 (15.7-24.5)	13.7 (9.4-18.1)	3.8 (1.5-6.5)	0.5
		South Africa	52.7 (43.0-62.3)	43.1 (33.9-52.9)	10.8 (6.9-15.1)	16.8 (12.7-21.4)	11.5 (7.8-15.7)	1.4 (-1.2-4.0)	14.0
	Lower-middle								
		Cabo Verde	75.0 (68.2-80.8)	67.1 (59.3-74.1)	15.8 (10.7-21.0)	24.0 (19.4-28.6)	16.4 (11.5-21.2)	7.0 (4.3-9.8)	0.1
		Cameroon	64.8 (57.2-71.8)	55.6 (47.6-63.4)	13.5 (9.1-18.0)	20.7 (16.5-25.2)	14.2 (9.9-18.6)	4.4 (2.2-6.9)	4.7
		Congo	68.7 (60.8-75.7)	59.9 (51.3-67.9)	14.3 (9.7-19.1)	21.9 (17.6-26.5)	15.0 (10.5-19.5)	5.3 (2.9-8.1)	1.0
		Côte d'Ivoire	62.7 (55.4-70.0)	53.3 (45.8-61.3)	13.0 (8.6-17.4)	20.0 (16.0-24.2)	13.7 (9.5-17.9)	3.8 (1.7-6.3)	5.7
		Eswatini	67.9 (61.8-74.0)	59.0 (52.4-65.9)	14.2 (9.6-18.9)	21.7 (17.5-25.9)	14.8 (10.5-19.1)	5.1 (3.0-7.5)	0.3
		Ghana	75.2 (70.2-80.1)	67.3 (61.6-73.2)	15.8 (10.7-20.8)	24.1 (19.7-28.4)	16.4 (11.6-21.2)	7.0 (4.5-9.6)	6.7
		Kenya	83.1 (80.5-85.7)	77.0 (73.7-80.3)	17.7 (12.3-22.9)	26.6 (22.0-30.9)	18.1 (12.8-23.2)	9.1 (6.2-11.8)	7.8
		Lesotho	94.0 (91.4-95.8)	91.4 (87.8-93.9)	20.3 (14.4-26.0)	30.0 (24.9-35.0)	20.5 (14.5-26.0)	12.0 (8.5-15.4)	1.4
		Mauritania	53.9 (45.6-62.3)	44.3 (36.3-52.9)	11.0 (7.1-15.2)	17.2 (13.3-21.2)	11.7 (8.0-15.6)	1.7 (-0.5-4.0)	0.5
		Nigeria	70.4 (63.7-76.3)	61.8 (54.4-68.6)	14.7 (10.0-19.5)	22.5 (18.2-27.0)	15.4 (10.8-20.0)	5.8 (3.4-8.4)	50.5

		São Tomé and Príncipe	79.0 (72.9-84.0)	71.9 (64.6-78.1)	16.7 (11.5-21.8)	25.3 (20.5-29.9)	17.3 (12.2-22.2)	8.0 (5.2-10.9)	0.0
		Zambia	75.0 (69.8-80.3)	67.1 (61.1-73.5)	15.8 (10.9-20.7)	24.0 (19.6-28.3)	16.4 (11.6-21.1)	7.0 (4.5-9.6)	2.8
	Low								
		Benin	81.9 (76.4-86.3)	75.5 (68.8-81.1)	17.5 (12.1-22.7)	26.1 (21.4-30.8)	17.9 (12.7-23.0)	8.8 (5.8-11.8)	2.5
		Burkina Faso	77.3 (71.6-83.0)	69.8 (63.2-76.8)	16.3 (11.2-21.5)	24.7 (20.2-29.3)	16.9 (11.8-21.7)	7.5 (4.9-10.4)	4.1
		Central African Republic	84.1 (79.3-87.9)	78.2 (72.3-83.2)	18.0 (12.5-23.4)	26.9 (22.0-31.5)	18.4 (13.0-23.5)	9.3 (6.4-12.5)	2.0
		Chad	73.1 (65.6-80.5)	64.9 (56.5-73.7)	15.4 (10.3-20.4)	23.4 (18.8-28.2)	16.0 (11.2-20.8)	6.5 (3.8-9.5)	3.0
		Comoros	81.0 (76.1-85.9)	74.3 (68.4-80.5)	17.2 (11.9-22.4)	25.9 (21.3-30.5)	17.7 (12.6-22.7)	8.5 (5.7-11.4)	0.2
		Democratic Republic of the Congo	73.2 (66.7-78.9)	65.0 (57.7-71.8)	15.4 (10.5-20.2)	23.4 (19.0-27.8)	16.0 (11.1-20.6)	6.5 (4.1-9.2)	15.1
		Eritrea	69.3 (64.3-74.2)	60.5 (55.0-66.2)	14.5 (9.9-19.1)	22.2 (18.0-26.2)	15.1 (10.7-19.5)	5.5 (3.5-7.7)	1.1
		Ethiopia	81.7 (78.1-85.4)	75.2 (70.8-79.9)	17.4 (12.1-22.4)	26.2 (21.6-30.6)	17.8 (12.6-22.8)	8.7 (5.9-11.5)	20.4
		Gambia	74.1 (67.0-80.2)	66.0 (58.0-73.4)	15.6 (10.6-20.7)	23.7 (19.1-28.4)	16.2 (11.4-20.9)	6.7 (4.1-9.7)	0.3
		Guinea	81.6 (75.9-86.2)	75.1 (68.2-80.9)	17.4 (12.1-22.7)	26.1 (21.3-30.6)	17.8 (12.4-22.9)	8.7 (5.7-11.8)	3.5
		Liberia	71.5 (64.5-77.6)	63.0 (55.3-70.2)	15.0 (10.2-19.9)	22.8 (18.5-27.4)	15.7 (11.0-20.3)	6.1 (3.6-8.8)	1.0
		Madagascar	78.6 (72.7-83.5)	71.4 (64.4-77.5)	16.6 (11.5-21.7)	25.1 (20.5-29.7)	17.2 (12.1-22.1)	7.8 (5.2-10.7)	4.8
		Malawi	81.8 (79.0-84.7)	75.3 (71.9-79.0)	17.4 (12.1-22.5)	26.1 (21.7-30.5)	17.8 (12.6-22.8)	8.7 (6.0-11.4)	3.4
		Mali	52.9 (45.3-60.5)	43.3 (36.0-51.0)	10.8 (7.1-14.8)	16.9 (13.2-20.9)	11.5 (7.9-15.3)	1.4 (-0.6-3.5)	2.5
		Mozambique	94.0 (91.8-95.6)	91.4 (88.4-93.7)	20.4 (14.5-26.1)	30.1 (25.0-35.1)	20.5 (14.5-26.2)	12.0 (8.5-15.4)	9.8
		Niger	75.0 (67.4-81.3)	67.1 (58.4-74.7)	15.8 (10.8-20.8)	24.0 (19.4-28.6)	16.4 (11.5-21.4)	6.9 (4.3-9.9)	3.6
		Rwanda	82.4 (77.3-86.6)	76.1 (69.8-81.5)	17.5 (11.9-22.7)	26.3 (21.5-31.0)	17.9 (12.8-23.0)	8.9 (5.9-11.9)	2.2
		Senegal	71.9 (66.0-77.7)	63.5 (56.9-70.3)	15.1 (10.4-20.0)	23.0 (18.7-27.4)	15.7 (11.0-20.3)	6.2 (3.8-8.8)	2.4
		Sierra Leone	81.7 (76.2-86.2)	75.2 (68.5-80.9)	17.5 (12.2-22.6)	26.2 (21.4-30.9)	17.8 (12.4-22.8)	8.7 (5.9-11.7)	2.9
		Togo	89.7 (86.4-92.2)	85.5 (81.2-88.9)	19.3 (13.5-24.9)	28.7 (23.6-33.5)	19.6 (14.0-25.1)	10.8 (7.5-14.1)	2.2
		Uganda	94.2 (92.0-95.8)	91.7 (88.7-93.9)	20.4 (14.4-26.2)	30.1 (25.0-35.1)	20.6 (14.6-26.2)	12.1 (8.5-15.5)	9.0
		United Republic of Tanzania	92.9 (90.5-94.7)	89.9 (86.6-92.4)	20.1 (14.2-25.7)	29.7 (24.5-34.6)	20.3 (14.4-25.8)	11.7 (8.2-15.0)	13.2
		Zimbabwe	69.3 (61.1-76.5)	60.5 (51.6-68.9)	14.4 (9.7-19.4)	22.2 (17.6-26.7)	15.1 (10.5-19.8)	5.5 (3.0-8.3)	3.7
Americas									

	High								
		Argentina	54.7 (45.8-63.4)	45.1 (36.5-54.1)	11.2 (7.1-15.5)	17.5 (13.5-21.7)	11.9 (8.1-15.8)	1.8 (-0.5-4.4)	5.9
		Bahamas	44.4 (35.3-54.0)	35.2 (27.1-44.4)	9.0 (5.5-12.9)	14.2 (10.5-18.2)	9.7 (6.4-13.3)	-0.6 (-3.2-1.8)	0.0
		Barbados	45.1 (35.5-54.6)	35.8 (27.2-45.0)	9.1 (5.7-13.0)	14.4 (10.6-18.6)	9.9 (6.5-13.6)	-0.5 (-3.0-1.9)	0.0
		Bermuda	67.9 (58.2-76.3)	59.0 (48.6-68.6)	14.2 (9.4-19.2)	21.7 (17.0-26.6)	14.8 (10.1-19.6)	5.1 (2.4-8.3)	0.0[e]
		British Virgin Islands	64.3 (55.3-72.4)	55.0 (45.7-64.1)	13.3 (8.8-18.1)	20.6 (16.1-25.3)	14.1 (9.8-18.5)	4.2 (1.8-7.0)	0.0[e]
		Canada	68.6 (60.3-75.9)	59.8 (50.8-68.2)	14.3 (9.5-19.2)	21.9 (17.6-26.6)	15.0 (10.4-19.6)	5.3 (2.8-8.1)	5.8
		Cayman Islands	61.9 (52.0-70.9)	52.5 (42.4-62.4)	12.8 (8.3-17.5)	19.7 (15.2-24.5)	13.5 (9.2-18.0)	3.6 (1.0-6.6)	0.0[e]
		Chile	71.4 (63.6-79.1)	62.9 (54.3-72.0)	15.0 (10.1-20.0)	22.9 (18.3-27.6)	15.6 (11.0-20.3)	6.0 (3.4-9.0)	3.4
		Saint Kitts and Nevis	59.6 (49.4-69.1)	50.1 (39.9-60.3)	12.2 (7.9-16.9)	19.0 (14.6-23.7)	13.0 (8.8-17.4)	3.0 (0.4-5.9)	0.0[e]
		Trinidad and Tobago	51.4 (38.8-63.7)	41.8 (30.1-54.4)	10.5 (6.3-15.2)	16.5 (11.5-21.9)	11.2 (7.2-15.9)	1.1 (-2.3-4.4)	0.3
		United States of America	52.0 (43.9-59.9)	42.4 (34.7-50.4)	10.6 (7.0-14.7)	16.7 (13.0-20.6)	11.4 (7.8-15.1)	1.2 (-0.8-3.4)	48.0
		Uruguay	74.3 (66.7-80.6)	66.3 (57.7-73.9)	15.6 (10.7-20.7)	23.7 (19.2-28.3)	16.2 (11.5-21.0)	6.8 (4.1-9.7)	0.8
	Upper-middle								
		Bolivarian Republic of Venezuela	70.5 (62.5-77.5)	61.9 (53.1-70.1)	14.8 (10.0-19.8)	22.6 (18.1-27.2)	15.4 (10.7-20.1)	5.8 (3.3-8.7)	5.5
		Brazil	46.7 (38.2-55.4)	37.3 (29.6-45.8)	9.5 (6.1-13.4)	15.0 (11.4-18.9)	10.2 (6.8-13.9)	-0.1 (-2.4-2.2)	24.7
		Colombia	51.1 (42.0-60.1)	41.5 (33.0-50.6)	10.4 (6.7-14.5)	16.4 (12.4-20.5)	11.2 (7.5-15.0)	1.0 (-1.4-3.4)	6.0
		Costa Rica	45.7 (37.1-54.5)	36.4 (28.6-44.9)	9.3 (5.9-13.1)	14.6 (11.0-18.5)	10.0 (6.7-13.4)	-0.3 (-2.6-2.0)	0.4
		Cuba	57.2 (48.3-65.7)	47.6 (38.8-56.6)	11.8 (7.7-16.0)	18.3 (14.3-22.7)	12.5 (8.6-16.7)	2.5 (0.3-4.9)	2.0
		Dominica	70.2 (61.9-77.3)	61.6 (52.5-69.8)	14.7 (9.9-19.7)	22.5 (17.9-27.2)	15.3 (10.7-20.0)	5.7 (3.2-8.6)	0.0[e]
		Dominican Republic	56.6 (47.4-65.4)	47.0 (38.0-56.2)	11.7 (7.6-16.0)	18.1 (13.9-22.6)	12.3 (8.4-16.5)	2.3 (0.0-4.9)	1.4
		Ecuador	70.3 (61.9-78.7)	61.7 (52.5-71.5)	14.7 (9.8-19.9)	22.5 (17.8-27.2)	15.4 (10.6-20.1)	5.7 (3.0-8.9)	2.1
		Grenada	64.6 (54.9-73.2)	55.4 (45.3-65.0)	13.4 (8.9-18.3)	20.7 (16.1-25.5)	14.1 (9.7-18.8)	4.3 (1.7-7.2)	0.0
		Guatemala	62.9 (52.1-72.5)	53.5 (42.5-64.2)	13.0 (8.5-17.9)	20.1 (15.4-25.2)	13.7 (9.4-18.6)	3.9 (1.1-7.0)	1.6
		Jamaica	63.4 (54.4-71.5)	54.1 (44.8-63.0)	13.1 (8.6-17.8)	20.3 (15.9-24.8)	13.8 (9.6-18.2)	4.0 (1.7-6.7)	0.4
		Mexico	67.8 (60.3-74.5)	58.9 (50.8-66.5)	14.2 (9.6-18.9)	21.7 (17.4-26.1)	14.8 (10.3-19.3)	5.1 (2.9-7.7)	16.6
		Paraguay	63.2 (54.7-71.8)	53.9 (45.1-63.4)	13.1 (8.7-17.9)	20.2 (15.8-24.7)	13.8 (9.6-18.2)	4.0 (1.5-6.8)	0.9

		Saint Lucia	47.9 (36.7-59.2)	38.5 (28.3-49.7)	9.7 (6.0-14.1)	15.4 (11.0-20.1)	10.5 (6.8-14.7)	0.2 (-2.7-3.2)	0.0
		Suriname	49.4 (40.7-58.0)	39.9 (31.8-48.4)	10.1 (6.4-14.0)	15.8 (12.1-19.8)	10.8 (7.3-14.5)	0.6 (-1.7-2.9)	0.1
Eastern Mediterranean									
	High								
		Kuwait	25.4 (18.7-33.4)	18.8 (13.5-25.4)	5.0 (2.8-7.6)	8.1 (5.4-11.2)	5.5 (3.4-8.1)	-5.0 (-7.8--2.7)	0.1
		Oman	59.8 (50.0-68.9)	50.3 (40.5-60.1)	12.4 (8.0-16.9)	19.1 (14.7-23.7)	13.0 (9.0-17.3)	3.1 (0.6-5.9)	0.2
		Qatar	51.3 (41.3-61.2)	41.7 (32.4-51.7)	10.4 (6.6-14.8)	16.4 (12.3-20.9)	11.2 (7.6-15.3)	1.0 (-1.6-3.7)	0.0
		Saudi Arabia	34.9 (26.6-44.2)	26.7 (19.8-35.0)	6.9 (4.1-10.3)	11.2 (7.8-14.9)	7.6 (4.8-10.8)	-2.8 (-5.6--0.6)	1.3
		United Arab Emirates	50.7 (41.5-59.8)	41.1 (32.5-50.3)	10.3 (6.6-14.4)	16.2 (12.3-20.4)	11.0 (7.5-15.0)	0.9 (-1.5-3.3)	0.2
	Upper-middle								
		Iraq	35.4 (27.6-44.2)	27.1 (20.6-35.0)	7.1 (4.3-10.4)	11.3 (8.1-15.0)	7.7 (5.0-10.9)	-2.7 (-5.3--0.5)	2.1
		Islamic Republic of Iran	76.9 (70.2-82.6)	69.4 (61.6-76.3)	16.3 (11.3-21.2)	24.6 (19.8-29.2)	16.8 (11.7-21.9)	7.4 (4.8-10.3)	11.3
		Jordan	86.5 (81.2-90.5)	81.3 (74.6-86.6)	18.6 (13.0-24.1)	27.7 (22.8-32.5)	18.8 (13.3-24.2)	10.0 (6.8-13.3)	1.3
		Lebanon	67.0 (58.0-75.0)	58.0 (48.4-67.1)	14.0 (9.3-18.7)	21.4 (17.0-26.1)	14.6 (10.1-19.2)	4.9 (2.3-7.8)	0.6
		Libya	58.5 (48.3-68.0)	48.9 (38.8-59.1)	12.0 (7.8-16.6)	18.7 (14.3-23.4)	12.8 (8.5-17.1)	2.8 (0.2-5.7)	0.7
	Lower-middle								
		Egypt	61.2 (51.5-70.1)	51.7 (41.9-61.4)	12.6 (8.3-17.3)	19.6 (15.1-24.3)	13.4 (9.1-17.9)	3.5 (1.0-6.3)	15.9
		Morocco	68.6 (58.3-77.4)	59.8 (48.7-70.0)	14.3 (9.4-19.3)	21.9 (17.0-26.9)	14.9 (10.2-19.7)	5.3 (2.5-8.4)	4.5
		Pakistan	56.7 (40.5-72.9)	47.1 (31.6-64.6)	11.6 (6.4-17.3)	18.1 (11.9-24.6)	12.4 (7.7-17.8)	2.3 (-2.4-6.9)	26.1
		Occupied Palestine Territory	51.2 (42.2-60.2)	41.6 (33.2-50.7)	10.4 (6.7-14.5)	16.4 (12.4-20.6)	11.2 (7.5-15.1)	1.0 (-1.3-3.4)	0.3
		Tunisia	65.9 (56.6-74.2)	56.8 (47.0-66.2)	13.7 (9.1-18.6)	21.1 (16.6-25.8)	14.4 (10.0-18.9)	4.7 (2.2-7.5)	1.8
Europe									
	High								
		Andorra	60.0 (51.5-68.0)	50.5 (41.9-59.1)	12.4 (8.2-16.8)	19.2 (15.0-23.5)	13.1 (9.0-17.3)	3.2 (0.9-5.6)	0.0[e]
		Austria	66.4 (58.9-73.2)	57.3 (49.3-65.0)	13.8 (9.3-18.4)	21.3 (16.9-25.7)	14.5 (10.1-18.9)	4.7 (2.5-7.2)	1.5
		Belgium	59.4 (51.6-66.7)	49.9 (42.0-57.7)	12.3 (8.1-16.7)	19.0 (15.0-23.1)	13.0 (9.1-17.1)	3.0 (1.0-5.3)	1.7
		Croatia	64.2 (55.3-72.1)	54.9 (45.7-63.7)	13.3 (8.8-18.1)	20.5 (16.2-25.0)	14.0 (9.6-18.5)	4.2 (1.8-7.0)	1.1
		Cyprus	49.5 (41.2-57.8)	40.0 (32.3-48.2)	10.1 (6.5-14.1)	15.8 (12.1-19.7)	10.8 (7.3-14.6)	0.6 (-1.6-2.9)	0.1

		Czechia	66.1 (57.6-73.7)	57.0 (48.0-65.6)	13.7 (9.1-18.6)	21.1 (16.8-25.9)	14.4 (10.0-19.1)	4.7 (2.3-7.4)	2.5
		Denmark	68.8 (61.6-75.2)	60.0 (52.2-67.3)	14.3 (9.8-19.0)	22.0 (17.8-26.4)	15.0 (10.6-19.6)	5.4 (3.1-7.9)	1.3
		Estonia	65.4 (56.9-73.0)	56.2 (47.3-64.8)	13.6 (9.0-18.3)	20.8 (16.4-25.3)	14.3 (9.9-18.9)	4.5 (2.1-7.2)	0.3
		Finland	84.0 (79.4-87.8)	78.1 (72.4-83.0)	17.8 (12.4-23.0)	26.9 (22.2-31.5)	18.4 (12.9-23.4)	9.3 (6.3-12.2)	1.3
		France	66.0 (58.6-72.7)	56.9 (49.0-64.4)	13.7 (9.2-18.3)	21.1 (16.8-25.5)	14.4 (10.1-18.8)	4.6 (2.5-7.1)	8.6
		Germany	55.9 (48.4-63.1)	46.3 (38.9-53.8)	11.5 (7.6-15.5)	17.8 (14.1-21.8)	12.2 (8.4-16.0)	2.1 (0.3-4.2)	12.9
		Greece	58.9 (51.0-66.3)	49.3 (41.4-57.2)	12.1 (8.0-16.5)	18.8 (14.8-23.0)	12.9 (8.8-17.0)	2.9 (0.9-5.2)	1.4
		Hungary	56.7 (47.8-65.2)	47.1 (38.4-56.0)	11.6 (7.6-16.1)	18.1 (14.1-22.4)	12.4 (8.5-16.5)	2.4 (0.1-4.9)	2.8
		Ireland	62.9 (55.3-69.9)	53.5 (45.7-61.2)	13.1 (8.8-17.6)	20.1 (16.1-24.5)	13.8 (9.5-18.1)	3.9 (1.8-6.3)	0.6
		Italy	53.8 (45.8-61.6)	44.2 (36.5-52.2)	11.1 (7.3-15.2)	17.2 (13.5-21.1)	11.7 (8.1-15.5)	1.7 (-0.4-3.8)	7.1
		Latvia	67.1 (58.5-74.7)	58.1 (48.9-66.7)	14.0 (9.3-18.8)	21.5 (17.1-26.2)	14.6 (10.2-19.3)	4.9 (2.4-7.7)	0.7
		Lithuania	70.8 (62.9-77.5)	62.2 (53.5-70.1)	14.8 (10.0-19.7)	22.6 (18.1-27.3)	15.4 (10.7-20.1)	5.9 (3.4-8.7)	1.0
		Luxembourg	69.6 (62.4-75.9)	60.9 (53.0-68.2)	14.5 (9.8-19.3)	22.3 (17.8-26.8)	15.2 (10.6-19.8)	5.6 (3.2-8.1)	0.1
		Malta	52.8 (43.6-61.7)	43.2 (34.4-52.3)	10.7 (6.9-15.0)	16.9 (13.0-21.1)	11.5 (7.8-15.5)	1.4 (-0.9-3.8)	0.1
		Netherlands	71.0 (64.0-77.1)	62.5 (54.7-69.6)	14.9 (10.0-19.6)	22.7 (18.2-27.1)	15.5 (10.7-20.3)	5.9 (3.5-8.6)	3.2
		Norway	66.2 (58.6-73.0)	57.1 (49.0-64.8)	13.8 (9.3-18.4)	21.1 (16.9-25.5)	14.4 (10.1-18.8)	4.7 (2.5-7.2)	0.7
		Poland	66.6 (58.1-74.3)	57.5 (48.5-66.3)	13.9 (9.3-18.6)	21.3 (16.9-25.8)	14.5 (10.1-19.1)	4.8 (2.4-7.6)	9.0
		Portugal	51.5 (42.4-60.5)	41.9 (33.4-51.0)	10.5 (6.7-14.7)	16.5 (12.6-20.7)	11.2 (7.6-15.1)	1.1 (-1.3-3.6)	1.3
		Slovakia	61.6 (52.9-69.7)	52.2 (43.3-61.0)	12.8 (8.4-17.3)	19.7 (15.4-24.2)	13.5 (9.3-17.7)	3.6 (1.2-6.1)	1.2
		Slovenia	63.4 (54.6-71.3)	54.1 (45.0-62.8)	13.2 (8.7-17.9)	20.3 (15.9-24.8)	13.8 (9.5-18.3)	4.0 (1.7-6.7)	0.3
		Spain	69.5 (62.3-75.8)	60.8 (52.9-68.0)	14.5 (9.8-19.3)	22.2 (17.7-26.6)	15.2 (10.6-19.6)	5.5 (3.3-8.2)	5.8
		Sweden	75.3 (68.9-80.7)	67.5 (60.1-74.0)	15.9 (10.8-20.9)	24.1 (19.5-28.6)	16.4 (11.6-21.2)	7.0 (4.4-9.9)	1.8
		Switzerland	74.3 (67.3-80.3)	66.3 (58.3-73.5)	15.6 (10.7-20.7)	23.8 (19.2-28.3)	16.2 (11.4-21.1)	6.8 (4.2-9.6)	1.2
		United Kingdom	60.0 (52.3-67.2)	50.5 (42.7-58.2)	12.4 (8.3-16.7)	19.2 (15.2-23.3)	13.1 (9.0-17.2)	3.2 (1.2-5.5)	10.0
	Upper-middle								
		Armenia	77.9 (69.7-84.4)	70.6 (61.0-78.6)	16.4 (11.2-21.8)	24.9 (20.0-30.0)	17.0 (11.8-22.0)	7.7 (4.7-10.9)	1.0
		Belarus	85.6 (79.9-89.9)	80.2 (73.0-85.8)	18.3 (12.8-23.8)	27.3 (22.4-32.2)	18.7 (13.4-24.1)	9.7 (6.6-13.0)	4.4
		Bosnia and Herzegovina	72.0 (62.7-79.7)	63.6 (53.3-72.7)	15.1 (10.2-20.2)	23.0 (18.2-28.1)	15.7 (10.9-20.7)	6.2 (3.4-9.4)	1.1

		Bulgaria	58.6 (49.3-67.2)	49.0 (39.8-58.2)	12.1 (7.9-16.6)	18.7 (14.5-23.4)	12.8 (8.7-17.2)	2.8 (0.5-5.4)	2.1
		Kazakhstan	71.3 (61.1-79.6)	62.8 (51.6-72.6)	14.9 (9.9-20.2)	22.8 (17.9-28.0)	15.6 (10.7-20.6)	6.0 (3.1-9.3)	5.0
		Romania	61.7 (52.6-70.0)	52.3 (43.0-61.3)	12.8 (8.4-17.4)	19.8 (15.4-24.2)	13.4 (9.2-17.8)	3.6 (1.2-6.3)	5.5
		Russian Federation	82.4 (77.2-87.6)	76.1 (69.7-82.8)	17.6 (12.1-22.9)	26.4 (21.6-31.1)	18.0 (12.7-23.1)	8.9 (5.9-12.0)	69.9
		Serbia	56.2 (46.4-65.6)	46.6 (37.0-56.5)	11.5 (7.4-16.0)	18.0 (13.7-22.5)	12.3 (8.3-16.5)	2.2 (-0.3-5.0)	2.4
		Turkey	61.2 (51.5-70.1)	51.7 (41.9-61.4)	12.7 (8.3-17.3)	19.6 (15.1-24.3)	13.4 (9.1-17.8)	3.5 (1.0-6.3)	9.2
	Lower-middle								
		Georgia	81.4 (74.1-87.1)	74.8 (66.0-82.1)	17.3 (11.9-22.7)	26.0 (21.0-31.0)	17.8 (12.5-23.0)	8.6 (5.5-11.9)	1.6
		Kyrgyzstan	83.3 (75.2-89.1)	77.2 (67.3-84.7)	17.8 (12.2-23.4)	26.6 (21.4-31.7)	18.2 (12.7-23.5)	9.1 (5.8-12.7)	1.2
		Republic of Moldova	89.1 (84.6-92.4)	84.7 (78.9-89.2)	19.1 (13.4-24.7)	28.5 (23.5-33.3)	19.5 (13.9-24.9)	10.7 (7.4-14.0)	2.0
		Ukraine	79.6 (72.4-85.3)	72.6 (64.1-79.8)	16.9 (11.6-22.3)	25.5 (20.7-30.4)	17.4 (12.2-22.5)	8.1 (5.2-11.4)	23.6
		Uzbekistan	75.6 (66.1-83.1)	67.8 (57.0-77.0)	15.9 (10.8-21.2)	24.2 (19.1-29.3)	16.5 (11.4-21.6)	7.1 (4.0-10.5)	6.0
	Low								
		Tajikistan	61.3 (50.1-71.5)	51.8 (40.6-63.0)	12.6 (8.1-17.5)	19.6 (15.0-24.5)	13.4 (9.1-18.0)	3.4 (0.6-6.6)	0.9
South-East Asia									
	Upper-middle								
		Maldives	65.2 (54.5-74.6)	56.0 (44.9-66.6)	13.5 (9.0-18.4)	20.9 (16.2-26.0)	14.2 (9.7-19.1)	4.4 (1.6-7.6)	0.0
		Thailand	72.8 (64.0-80.0)	64.5 (54.7-73.1)	15.2 (10.3-20.4)	23.3 (18.5-28.2)	15.9 (11.1-20.8)	6.3 (3.6-9.5)	17.2
	Lower-middle								
		Bangladesh	60.5 (44.2-76.9)	51.0 (35.0-69.4)	12.4 (7.0-18.4)	19.3 (12.9-26.1)	13.2 (8.2-18.8)	3.2 (-1.5-8.0)	23.6
		Bhutan	70.5 (63.9-77.2)	61.9 (54.6-69.7)	14.8 (10.0-19.5)	22.5 (18.2-27.0)	15.4 (10.8-20.0)	5.8 (3.4-8.5)	0.2
		India	56.1 (40.3-70.8)	46.5 (31.5-62.2)	11.5 (6.5-16.9)	17.9 (12.0-24.0)	12.3 (7.6-17.5)	2.2 (-2.1-6.5)	242.4
		Indonesia	78.3 (69.8-85.0)	71.0 (61.1-79.4)	16.6 (11.2-21.9)	25.0 (20.1-30.0)	17.1 (11.9-22.2)	7.8 (4.8-11.2)	76.1
		Myanmar	86.9 (81.5-90.8)	81.8 (75.0-87.0)	18.6 (12.9-24.2)	27.8 (22.9-32.6)	18.9 (13.4-24.2)	10.1 (6.8-13.4)	20.3
		Sri Lanka	63.3 (52.5-72.8)	54.0 (42.9-64.5)	13.1 (8.5-18.1)	20.2 (15.5-25.2)	13.8 (9.3-18.4)	3.9 (1.0-7.1)	3.9
		Timor-Leste	74.5 (64.8-82.3)	66.5 (55.6-76.0)	15.6 (10.5-20.9)	23.9 (18.9-29.0)	16.3 (11.1-21.3)	6.8 (3.7-10.2)	0.2
	Low								
		Nepal	85.4 (83.5-87.3)	79.9 (77.5-82.4)	18.2 (12.6-23.5)	27.3 (22.5-31.8)	18.6 (13.1-23.7)	9.7 (6.6-12.5)	8.3

Western Pacific									
	High								
		Australia	66.4 (58.1-73.7)	57.3 (48.5-65.6)	13.8 (9.2-18.6)	21.2 (16.9-25.8)	14.5 (10.0-18.9)	4.7 (2.3-7.4)	2.8
		Brunei Darussalam	66.1 (53.6-76.7)	57.0 (44.0-69.1)	13.7 (8.8-18.9)	21.1 (15.9-26.8)	14.5 (9.7-19.7)	4.6 (1.3-8.2)	0.0
		French Polynesia	77.5 (69.6-83.8)	70.1 (60.9-77.9)	16.4 (11.2-21.6)	24.7 (19.9-29.7)	16.9 (11.8-21.9)	7.6 (4.7-10.7)	0.1
		Japan	63.0 (43.5-79.0)	53.6 (34.4-71.9)	13.0 (7.3-19.3)	20.1 (13.3-27.3)	13.8 (8.3-19.7)	3.9 (-1.4-9.0)	16.4
		New Zealand	54.7 (46.8-62.4)	45.1 (37.4-53.0)	11.2 (7.4-15.3)	17.5 (13.7-21.5)	11.9 (8.2-15.9)	1.9 (-0.1-4.1)	0.5
		Palau	46.5 (35.3-58.1)	37.1 (27.1-48.5)	9.4 (5.6-13.8)	14.8 (10.5-19.5)	10.1 (6.5-14.2)	-0.1 (-3.2-2.9)	0.0[e]
		Republic of Korea	59.0 (40.1-75.6)	49.4 (31.3-67.8)	12.1 (6.7-18.2)	18.9 (12.2-25.8)	12.8 (7.6-18.6)	2.8 (-2.3-7.8)	4.7
		Singapore	61.4 (42.5-77.4)	52.0 (33.4-70.0)	12.6 (7.2-18.7)	19.6 (12.8-26.8)	13.3 (7.9-19.5)	3.5 (-1.4-8.4)	0.6
	Upper-middle								
		American Samoa	42.5 (30.8-55.1)	33.4 (23.2-45.5)	8.6 (4.9-12.9)	13.6 (9.3-18.5)	9.2 (5.8-13.3)	-1.1 (-4.6-2.2)	0.0[e]
		China	87.8 (82.7-91.5)	83.0 (76.5-88.0)	18.8 (13.0-24.6)	28.1 (23.1-33.0)	19.1 (13.5-24.5)	10.3 (7.0-13.8)	431.9
		Cook Islands	72.8 (63.8-80.3)	64.5 (54.5-73.5)	15.3 (10.3-20.4)	23.3 (18.5-28.2)	15.9 (11.1-20.8)	6.4 (3.6-9.5)	0.0[e]
		Fiji	75.9 (67.8-82.5)	68.2 (58.9-76.2)	16.0 (10.8-21.3)	24.2 (19.5-29.1)	16.6 (11.6-21.5)	7.2 (4.3-10.4)	0.3
		Malaysia	57.2 (46.5-67.3)	47.6 (37.1-58.3)	11.7 (7.5-16.3)	18.3 (13.8-23.1)	12.5 (8.4-16.9)	2.4 (-0.3-5.4)	3.7
		Marshall Islands	50.0 (39.2-60.7)	40.5 (30.5-51.2)	10.2 (6.3-14.5)	16.0 (11.7-20.5)	10.9 (7.2-15.0)	0.7 (-2.0-3.5)	0.0[e]
		Nauru	50.6 (38.7-62.4)	41.0 (30.0-53.0)	10.3 (6.3-14.7)	16.2 (11.6-21.3)	11.1 (7.2-15.5)	0.9 (-2.2-3.9)	0.0[e]
		Samoa	82.8 (75.8-88.1)	76.6 (68.0-83.4)	17.6 (12.1-23.1)	26.5 (21.5-31.6)	18.1 (12.9-23.3)	9.0 (5.8-12.3)	0.0
		Tonga	74.1 (65.2-81.4)	66.0 (56.0-74.8)	15.6 (10.6-20.9)	23.7 (18.9-28.5)	16.2 (11.1-21.1)	6.7 (3.9-10.0)	0.0
		Tuvalu	62.8 (53.0-71.7)	53.4 (43.4-63.3)	13.0 (8.6-17.8)	20.0 (15.5-24.8)	13.8 (9.4-18.3)	3.8 (1.3-6.7)	0.0[e]
	Lower-middle								
		Cambodia	88.9 (83.2-92.8)	84.5 (77.1-89.8)	19.1 (13.3-24.7)	28.4 (23.3-33.4)	19.4 (13.8-25.0)	10.6 (7.2-14.0)	4.9
		Federated States of Micronesia	67.1 (58.4-74.7)	58.1 (48.8-66.7)	14.0 (9.3-18.8)	21.5 (17.0-26.1)	14.6 (10.2-19.2)	4.9 (2.5-7.7)	0.0
		Kiribati	54.2 (43.8-64.2)	44.6 (34.6-54.9)	11.1 (7.0-15.6)	17.3 (13.0-22.0)	11.9 (7.9-16.2)	1.7 (-1.0-4.6)	0.0
		Lao People's Democratic Republic	79.4 (73.1-85.6)	72.4 (64.9-80.2)	16.8 (11.5-22.0)	25.4 (20.6-30.1)	17.3 (12.2-22.3)	8.1 (5.2-11.1)	1.7
		Mongolia	80.6 (74.2-85.7)	73.9 (66.2-80.3)	17.1 (11.8-22.4)	25.7 (20.9-30.4)	17.6 (12.3-22.7)	8.4 (5.5-11.5)	0.7

		Niue	94.0 (91.2-95.9)	91.4 (87.6-94.1)	20.4 (14.5-26.1)	30.1 (24.8-35.0)	20.5 (14.5-26.2)	12.0 (8.5-15.5)	0.0[e]
		Papua New Guinea	81.8 (73.1-88.2)	75.3 (64.9-83.6)	17.4 (11.9-23.0)	26.1 (21.0-31.3)	17.8 (12.5-23.1)	8.7 (5.5-12.3)	2.1
		Philippines	50.9 (40.9-60.9)	41.3 (32.0-51.4)	10.4 (6.5-14.7)	16.3 (12.2-20.8)	11.1 (7.4-15.1)	0.9 (-1.7-3.7)	14.2
		Solomon Islands	76.8 (68.5-83.5)	69.2 (59.6-77.5)	16.2 (11.1-21.5)	24.6 (19.8-29.4)	16.7 (11.7-21.7)	7.4 (4.5-10.7)	0.1
		Tokelau	82.3 (74.6-88.1)	76.0 (66.6-83.4)	17.5 (12.1-23.0)	26.3 (21.3-31.3)	17.9 (12.6-23.3)	8.9 (5.7-12.3)	0.0[e]
		Vanuatu	91.2 (87.2-94.1)	87.6 (82.2-91.6)	19.7 (14.0-25.4)	29.2 (24.1-34.1)	19.9 (14.1-25.4)	11.2 (7.9-14.7)	0.1
		Viet Nam	69.4 (60.4-77.2)	60.7 (50.9-69.7)	14.5 (9.7-19.5)	22.2 (17.5-27.1)	15.1 (10.4-19.9)	5.5 (2.8-8.6)	12.0

Appendix 13B. The Prevented Fractions for the Population and corresponding number of deaths averted for men, by country, region and income group.

					Prevented Fractions for the Population (95% confidence intervals)				
World Health Organization region	World Bank income group classification	Country	Prevalence of activity (95% confidence intervals)	Prevalence of activity amongst those that died (95% confidence intervals)	Main adjusted estimate (0% activity counterfactual)[a]	Unadjusted estimate (0% activity counterfactual)[b]	Partially adjusted estimate (0% activity counterfactual)[c]	Adjusted estimate (54.3% active counterfactual)[d]	Number of deaths averted between ages of 40-74 years (thousands)
Africa									
	High								
		Seychelles	82.3 (76.7-86.8)	76.0 (69.1-81.7)	17.5 (12.1-22.8)	26.4 (21.6-31.1)	17.9 (12.5-23.0)	5.9 (3.7-8.4)	0.1
	Upper-middle								
		Algeria	73.1 (64.9-80.0)	64.9 (55.7-73.1)	15.4 (10.3-20.6)	23.3 (18.6-28.1)	15.9 (11.2-20.7)	3.5 (1.2-6.0)	6.6
		Botswana	82.9 (78.3-86.7)	76.7 (71.0-81.6)	17.6 (12.3-23.0)	26.5 (21.8-31.0)	18.2 (12.9-23.3)	6.1 (4.0-8.4)	0.9
		Gabon	82.3 (75.8-87.4)	76.0 (68.0-82.5)	17.5 (12.0-23.0)	26.3 (21.4-31.2)	18.0 (12.7-23.1)	5.9 (3.5-8.5)	0.6
		Mauritius	72.4 (64.9-78.8)	64.1 (55.7-71.6)	15.1 (10.3-20.1)	23.1 (18.6-27.8)	15.8 (11.1-20.6)	3.3 (1.2-5.6)	0.6
		Namibia	71.1 (64.4-77.0)	62.6 (55.2-69.5)	14.9 (10.2-19.7)	22.7 (18.4-27.1)	15.5 (10.7-20.2)	3.0 (1.1-5.0)	0.7
		South Africa	71.5 (64.1-78.0)	63.0 (54.8-70.7)	15.0 (10.3-19.8)	22.8 (18.4-27.3)	15.6 (10.9-20.2)	3.1 (1.0-5.3)	31.2
	Lower-middle								
		Cabo Verde	85.9 (81.6-89.4)	80.5 (75.1-85.1)	18.4 (12.7-23.9)	27.5 (22.6-32.1)	18.8 (13.4-24.0)	6.9 (4.6-9.3)	0.1
		Cameroon	78.2 (72.6-83.0)	70.9 (64.3-76.8)	16.5 (11.4-21.5)	25.0 (20.5-29.4)	17.1 (12.0-22.0)	4.8 (2.9-7.0)	6.6

		Congo	75.2 (68.3-81.1)	67.3 (59.4-74.5)	15.8 (10.8-20.9)	24.1 (19.5-28.7)	16.4 (11.4-21.2)	4.0 (2.0-6.3)	1.2
		Côte d'Ivoire	70.9 (65.5-76.4)	62.4 (56.3-68.8)	14.9 (10.1-19.7)	22.7 (18.5-26.9)	15.5 (11.0-19.9)	2.9 (1.3-4.8)	8.6
		Eswatini	76.2 (72.7-79.8)	68.5 (64.4-72.9)	16.1 (11.2-20.9)	24.3 (20.1-28.5)	16.6 (11.8-21.2)	4.3 (2.7-6.0)	0.5
		Ghana	81.4 (77.4-85.4)	74.8 (70.0-79.9)	17.3 (11.9-22.3)	26.0 (21.4-30.5)	17.8 (12.6-22.8)	5.7 (3.7-7.8)	8.1
		Kenya	86.1 (84.0-88.2)	80.8 (78.1-83.6)	18.4 (12.9-23.8)	27.5 (22.8-32.1)	18.7 (13.1-24.0)	7.0 (4.8-9.1)	10.2
		Lesotho	93.5 (91.3-95.1)	90.7 (87.7-93.0)	20.3 (14.3-25.9)	29.9 (24.8-34.7)	20.4 (14.4-26.1)	9.1 (6.4-11.6)	1.4
		Mauritania	63.5 (56.1-70.8)	54.2 (46.5-62.2)	13.2 (8.9-17.8)	20.3 (16.3-24.5)	13.8 (9.6-18.1)	1.0 (-1.1-3.1)	0.7
		Nigeria	75.3 (69.2-80.5)	67.5 (60.4-73.7)	15.9 (11.0-21.0)	24.1 (19.6-28.4)	16.4 (11.6-21.1)	4.1 (2.2-6.2)	59.8
		São Tomé and Príncipe	90.2 (87.0-92.7)	86.2 (82.0-89.6)	19.5 (13.7-25.1)	28.9 (23.7-33.7)	19.7 (14.2-25.2)	8.1 (5.6-10.7)	0.0
		Zambia	80.9 (76.7-85.2)	74.2 (69.1-79.6)	17.2 (11.9-22.3)	25.9 (21.3-30.3)	17.7 (12.3-22.7)	5.6 (3.6-7.7)	3.5
	Low								
		Benin	86.4 (82.5-89.5)	81.2 (76.2-85.3)	18.5 (13.0-23.9)	27.6 (22.6-32.3)	18.9 (13.2-24.2)	7.1 (4.8-9.4)	2.7
		Burkina Faso	82.3 (77.6-86.9)	76.0 (70.2-81.8)	17.5 (12.2-22.8)	26.3 (21.6-31.0)	17.9 (12.7-23.0)	6.0 (3.8-8.3)	3.9
		Central African Republic	87.4 (83.7-90.3)	82.5 (77.7-86.4)	18.7 (13.2-24.1)	27.9 (23.0-32.6)	19.1 (13.6-24.2)	7.3 (5.0-9.7)	2.2
		Chad	80.4 (74.4-86.4)	73.6 (66.4-81.2)	17.1 (11.7-22.4)	25.7 (20.9-30.5)	17.5 (12.4-22.6)	5.4 (3.2-8.0)	3.7
		Comoros	90.4 (88.1-92.6)	86.5 (83.4-89.5)	19.5 (13.7-25.1)	28.9 (23.7-33.5)	19.7 (14.0-25.1)	8.2 (5.7-10.6)	0.2
		Democratic Republic of the Congo	79.2 (73.5-83.9)	72.1 (65.3-78.0)	16.8 (11.6-21.8)	25.3 (20.7-30.0)	17.3 (12.1-22.2)	5.1 (3.1-7.3)	18.1
		Eritrea	86.2 (84.1-88.4)	80.9 (78.2-83.8)	18.4 (12.9-23.8)	27.5 (22.8-32.1)	18.8 (13.3-24.0)	7.0 (4.8-9.2)	1.7
		Ethiopia	88.7 (86.8-90.5)	84.2 (81.7-86.6)	19.0 (13.4-24.4)	28.4 (23.6-33.0)	19.3 (13.8-24.5)	7.7 (5.4-10.0)	25.7
		Gambia	84.0 (79.1-88.0)	78.1 (72.0-83.3)	17.9 (12.6-23.1)	26.9 (22.0-31.5)	18.4 (13.2-23.6)	6.4 (4.2-8.8)	0.5
		Guinea	89.4 (85.8-92.1)	85.1 (80.4-88.8)	19.2 (13.5-24.8)	28.5 (23.6-33.3)	19.5 (13.9-25.0)	7.9 (5.4-10.4)	4.3
		Liberia	78.3 (72.8-82.9)	71.0 (64.5-76.7)	16.6 (11.5-21.7)	25.0 (20.5-29.6)	17.1 (12.1-22.0)	4.9 (3.0-7.1)	1.2
		Madagascar	87.2 (83.4-90.2)	82.2 (77.4-86.2)	18.7 (13.0-23.9)	27.9 (23.1-32.5)	19.0 (13.4-24.2)	7.3 (5.0-9.7)	6.5
		Malawi	87.0 (85.3-88.7)	82.0 (79.8-84.2)	18.7 (13.2-24.0)	27.8 (22.9-32.3)	19.0 (13.5-24.2)	7.3 (5.1-9.4)	5.0
		Mali	66.3 (60.2-72.4)	57.2 (50.7-64.1)	13.8 (9.4-18.3)	21.2 (17.1-25.4)	14.5 (10.1-18.8)	1.7 (0.0-3.6)	2.9
		Mozambique	95.0 (93.2-96.3)	92.8 (90.3-94.7)	20.6 (14.6-26.3)	30.4 (25.2-35.3)	20.7 (14.7-26.3)	9.4 (6.7-12.1)	10.1
		Niger	80.3 (73.5-85.7)	73.5 (65.3-80.3)	17.0 (11.8-22.4)	25.7 (20.9-30.5)	17.5 (12.4-22.6)	5.4 (3.1-8.0)	4.8

		Rwanda	89.0 (85.5-91.7)	84.6 (80.0-88.2)	19.1 (13.4-24.7)	28.5 (23.4-33.2)	19.4 (13.8-24.8)	7.8 (5.3-10.3)	2.6
		Senegal	82.4 (79.6-85.3)	76.1 (72.6-79.8)	17.5 (12.3-22.7)	26.4 (21.8-30.7)	18.0 (12.8-22.9)	6.0 (4.1-8.0)	3.2
		Sierra Leone	89.9 (86.5-92.4)	85.8 (81.3-89.2)	19.3 (13.6-24.8)	28.7 (23.6-33.5)	19.5 (13.7-25.1)	8.0 (5.6-10.5)	3.1
		Togo	90.8 (88.0-93.0)	87.0 (83.3-90.0)	19.6 (13.9-25.3)	29.0 (24.0-33.8)	19.8 (14.0-25.2)	8.3 (5.8-10.9)	2.5
		Uganda	94.8 (92.9-96.2)	92.5 (89.9-94.5)	20.6 (14.7-26.3)	30.3 (25.1-35.3)	20.7 (14.6-26.4)	9.4 (6.7-12.1)	10.2
		United Republic of Tanzania	94.2 (92.3-95.7)	91.7 (89.1-93.8)	20.4 (14.4-26.2)	30.1 (24.9-35.1)	20.6 (14.6-26.2)	9.2 (6.5-11.9)	15.9
		Zimbabwe	77.2 (70.5-82.8)	69.7 (61.9-76.6)	16.3 (11.2-21.5)	24.7 (20.0-29.3)	16.9 (11.8-21.6)	4.6 (2.5-6.9)	4.2
Americas									
	High								
		Argentina	62.4 (54.5-69.6)	53.0 (44.9-60.9)	12.9 (8.6-17.4)	20.0 (15.8-24.2)	13.6 (9.4-17.8)	0.7 (-1.4-2.7)	12.1
		Bahamas	70.0 (61.9-77.0)	61.3 (52.5-69.5)	14.6 (9.8-19.5)	22.4 (17.8-27.0)	15.3 (10.6-19.9)	2.7 (0.5-5.1)	0.1
		Barbados	70.7 (62.7-78.7)	62.1 (53.3-71.5)	14.8 (10.0-19.9)	22.6 (18.0-27.2)	15.4 (10.7-20.2)	2.9 (0.5-5.5)	0.1
		Bermuda	79.7 (72.7-85.3)	72.7 (64.4-79.8)	16.8 (11.6-22.1)	25.5 (20.7-30.4)	17.4 (12.2-22.5)	5.2 (2.9-7.8)	0.0[e]
		British Virgin Islands	80.9 (74.8-85.9)	74.2 (66.9-80.5)	17.2 (11.9-22.5)	25.8 (21.1-30.5)	17.6 (12.4-22.7)	5.6 (3.4-8.0)	0.0[e]
		Canada	74.3 (67.2-80.3)	66.3 (58.2-73.5)	15.6 (10.7-20.6)	23.8 (19.3-28.4)	16.2 (11.3-21.1)	3.8 (1.7-6.1)	9.4
		Cayman Islands	79.8 (73.0-85.3)	72.9 (64.8-79.8)	16.9 (11.5-22.2)	25.5 (20.7-30.3)	17.4 (12.2-22.5)	5.3 (3.0-7.8)	0.0[e]
		Chile	75.6 (69.4-81.8)	67.8 (60.7-75.3)	15.9 (11.0-20.9)	24.2 (19.6-28.7)	16.5 (11.7-21.3)	4.1 (2.1-6.4)	5.0
		Saint Kitts and Nevis	75.9 (68.1-82.2)	68.2 (59.2-75.8)	16.0 (10.9-21.2)	24.3 (19.5-29.1)	16.6 (11.6-21.4)	4.2 (1.9-6.8)	0.0[e]
		Trinidad and Tobago	72.8 (62.5-81.1)	64.5 (53.1-74.5)	15.3 (10.2-20.6)	23.3 (18.2-28.4)	15.9 (10.8-21.0)	3.4 (0.5-6.5)	0.7
		United States of America	68.3 (61.7-74.2)	59.4 (52.3-66.2)	14.3 (9.7-19.0)	21.8 (17.7-26.2)	14.9 (10.3-19.4)	2.2 (0.5-4.2)	97.9
		Uruguay	81.3 (75.7-85.8)	74.7 (67.9-80.4)	17.3 (11.9-22.6)	26.0 (21.3-30.7)	17.7 (12.4-22.8)	5.7 (3.5-8.0)	1.5
	Upper-middle								
		Bolivarian Republic of Venezuela	68.6 (60.0-76.0)	59.8 (50.5-68.3)	14.3 (9.6-19.2)	22.0 (17.5-26.6)	15.0 (10.3-19.7)	2.3 (0.0-4.8)	7.8
		Brazil	59.6 (51.5-67.2)	50.1 (41.9-58.2)	12.3 (8.2-16.6)	19.0 (14.9-23.2)	13.0 (9.0-17.1)	0.0 (-2.2-2.1)	49.6
		Colombia	61.2 (53.0-69.5)	51.7 (43.4-60.8)	12.7 (8.4-17.2)	19.6 (15.4-23.9)	13.4 (9.2-17.6)	0.4 (-2.0-2.7)	9.9
		Costa Rica	62.3 (54.3-69.7)	52.9 (44.7-61.0)	12.9 (8.6-17.4)	19.9 (15.9-24.2)	13.6 (9.4-17.9)	0.6 (-1.5-2.8)	0.9
		Cuba	69.1 (61.3-75.9)	60.3 (51.8-68.2)	14.4 (9.8-19.3)	22.1 (17.7-26.7)	15.1 (10.5-19.7)	2.4 (0.3-4.7)	3.6

		Dominica	86.6 (82.0-90.1)	81.5 (75.6-86.1)	18.5 (13.0-24.0)	27.7 (22.8-32.5)	18.9 (13.4-24.3)	7.1 (4.8-9.6)	0.0[e]
		Dominican Republic	65.6 (57.4-72.9)	56.5 (47.8-64.6)	13.6 (9.1-18.5)	21.0 (16.6-25.5)	14.3 (9.9-18.9)	1.5 (-0.6-3.8)	2.5
		Ecuador	75.4 (68.9-81.9)	67.6 (60.1-75.5)	15.9 (10.8-21.0)	24.1 (19.5-28.8)	16.5 (11.6-21.4)	4.1 (2.0-6.5)	3.3
		Grenada	78.1 (70.9-84.0)	70.8 (62.4-78.1)	16.5 (11.3-21.8)	24.9 (20.1-29.6)	17.1 (12.0-22.0)	4.8 (2.5-7.4)	0.0
		Guatemala	62.9 (53.3-71.6)	53.5 (43.7-63.2)	13.0 (8.4-17.7)	20.1 (15.5-24.9)	13.7 (9.4-18.2)	0.8 (-1.8-3.3)	2.1
		Jamaica	71.6 (64.1-78.1)	63.2 (54.8-70.8)	15.0 (10.2-19.9)	22.9 (18.2-27.6)	15.6 (11.0-20.4)	3.1 (1.0-5.4)	0.7
		Mexico	74.5 (68.6-79.7)	66.5 (59.8-72.7)	15.7 (10.9-20.6)	23.8 (19.4-28.3)	16.3 (11.5-20.9)	3.8 (2.0-5.9)	25.2
		Paraguay	61.9 (53.5-70.4)	52.5 (43.9-61.8)	12.8 (8.5-17.5)	19.8 (15.5-24.2)	13.5 (9.2-17.8)	0.5 (-1.9-3.0)	1.2
		Saint Lucia	73.4 (64.4-80.8)	65.2 (55.2-74.1)	15.4 (10.5-20.5)	23.5 (18.6-28.4)	16.0 (11.1-20.9)	3.6 (1.0-6.3)	0.1
		Suriname	61.9 (53.9-69.2)	52.5 (44.3-60.4)	12.8 (8.6-17.2)	19.8 (15.7-24.1)	13.6 (9.4-17.9)	0.6 (-1.6-2.7)	0.2
Eastern Mediterranean									
	High								
		Kuwait	38.7 (30.8-47.2)	30.0 (23.2-37.8)	7.8 (4.8-11.1)	12.4 (9.1-16.1)	8.4 (5.5-11.5)	-5.2 (-8.3--2.7)	0.3
		Oman	70.0 (61.6-77.2)	61.3 (52.2-69.7)	14.7 (9.9-19.6)	22.4 (17.9-27.0)	15.3 (10.6-20.0)	2.7 (0.4-5.1)	0.5
		Qatar	66.8 (57.7-74.9)	57.8 (48.1-67.0)	13.9 (9.3-18.8)	21.3 (16.8-26.1)	14.6 (10.1-19.2)	1.8 (-0.6-4.3)	0.2
		Saudi Arabia	55.1 (46.6-63.3)	45.5 (37.2-54.0)	11.3 (7.4-15.6)	17.6 (13.8-21.7)	12.0 (8.2-16.0)	-1.2 (-3.6-1.0)	4.1
	Upper-middle								
		United Arab Emirates	61.0 (52.6-69.5)	51.5 (43.0-60.8)	12.6 (8.3-17.2)	19.5 (15.3-23.9)	13.3 (9.2-17.6)	0.3 (-2.1-2.7)	0.9
		Iraq	60.5 (52.8-67.8)	51.0 (43.2-58.9)	12.5 (8.4-16.9)	19.3 (15.3-23.5)	13.2 (9.1-17.3)	0.2 (-1.8-2.2)	5.3
		Islamic Republic of Iran	66.8 (58.6-74.1)	57.8 (49.0-66.0)	13.9 (9.2-18.7)	21.3 (17.0-25.9)	14.6 (10.2-19.0)	1.8 (-0.4-4.1)	12.5
		Jordan	89.7 (85.8-92.6)	85.5 (80.4-89.5)	19.4 (13.5-24.8)	28.7 (23.6-33.6)	19.6 (13.8-25.1)	8.0 (5.5-10.6)	1.9
		Lebanon	60.2 (51.3-68.5)	50.7 (41.7-59.6)	12.4 (8.2-16.9)	19.3 (14.9-23.8)	13.1 (9.0-17.4)	0.1 (-2.3-2.5)	0.9
		Libya	68.8 (60.4-76.1)	60.0 (50.9-68.4)	14.4 (9.6-19.3)	22.0 (17.6-26.6)	15.0 (10.6-19.5)	2.3 (0.1-4.8)	1.4
	Lower-middle								
		Egypt	76.8 (69.5-82.7)	69.2 (60.8-76.5)	16.2 (11.1-21.3)	24.6 (19.9-29.2)	16.8 (11.7-21.7)	4.5 (2.3-6.9)	29.7
		Morocco	79.3 (71.2-85.5)	72.3 (62.7-80.0)	16.8 (11.4-22.2)	25.4 (20.4-30.3)	17.3 (12.0-22.3)	5.1 (2.7-7.9)	6.5
		Pakistan	75.6 (64.9-86.3)	67.8 (55.7-81.1)	15.9 (10.5-21.6)	24.1 (18.6-29.8)	16.5 (11.3-22.0)	4.1 (0.6-7.6)	46.3
		Occupied Palestine	67.6 (59.6-74.6)	58.6 (50.1-66.6)	14.1 (9.5-18.9)	21.6 (17.3-26.1)	14.7 (10.3-19.3)	2.0 (0.0-4.2)	0.6

		Territory							
		Tunisia	73.6 (66.3-79.8)	65.5 (57.2-72.9)	15.4 (10.5-20.5)	23.5 (19.0-28.2)	16.1 (11.3-20.9)	3.6 (1.5-6.0)	3.2
Europe									
	High								
		Andorra	63.2 (55.4-70.3)	53.9 (45.8-61.7)	13.1 (8.7-17.7)	20.2 (16.1-24.5)	13.8 (9.6-18.1)	0.9 (-1.2-2.9)	0.0[e]
		Austria	73.6 (67.7-78.8)	65.5 (58.8-71.6)	15.5 (10.5-20.4)	23.5 (19.2-28.0)	16.0 (11.3-20.7)	3.6 (1.8-5.7)	2.9
		Belgium	69.4 (63.2-75.0)	60.7 (53.9-67.1)	14.5 (9.9-19.3)	22.2 (18.0-26.5)	15.1 (10.7-19.6)	2.5 (0.8-4.4)	3.4
		Croatia	74.1 (67.3-80.0)	66.0 (58.3-73.1)	15.6 (10.6-20.6)	23.7 (19.1-28.4)	16.2 (11.4-20.9)	3.7 (1.7-6.0)	2.4
		Cyprus	61.6 (54.4-68.4)	52.2 (44.8-59.5)	12.7 (8.5-17.0)	19.7 (15.6-23.9)	13.4 (9.4-17.6)	0.5 (-1.4-2.4)	0.2
		Czechia	71.9 (64.9-78.0)	63.5 (55.7-70.7)	15.0 (10.1-19.9)	23.0 (18.5-27.5)	15.7 (11.0-20.3)	3.1 (1.2-5.4)	5.0
		Denmark	74.3 (68.5-79.3)	66.3 (59.6-72.3)	15.6 (10.7-20.6)	23.7 (19.3-28.1)	16.2 (11.5-20.8)	3.8 (2.0-5.8)	2.1
		Estonia	71.1 (64.2-77.2)	62.6 (54.9-69.7)	14.9 (10.1-19.9)	22.7 (18.5-27.1)	15.5 (10.9-20.1)	2.9 (1.0-5.2)	0.7
		Finland	82.8 (78.5-86.4)	76.6 (71.3-81.2)	17.6 (12.2-23.1)	26.5 (21.7-31.2)	18.0 (12.7-23.1)	6.1 (4.0-8.4)	2.4
		France	75.7 (70.1-80.6)	67.9 (61.4-73.9)	16.0 (11.0-21.0)	24.2 (19.7-28.7)	16.5 (11.6-21.2)	4.2 (2.4-6.2)	20.8
		Germany	59.8 (53.3-66.0)	50.3 (43.7-56.9)	12.3 (8.3-16.5)	19.1 (15.3-23.0)	13.1 (9.0-17.0)	0.0 (-1.7-1.7)	24.8
		Greece	66.1 (59.5-72.1)	57.0 (50.0-63.7)	13.8 (9.4-18.3)	21.1 (17.2-25.3)	14.4 (10.2-18.7)	1.6 (-0.1-3.5)	3.3
		Hungary	66.9 (59.4-73.6)	57.9 (49.9-65.5)	13.9 (9.4-18.6)	21.4 (17.2-25.8)	14.6 (10.3-19.1)	1.8 (-0.1-4.0)	5.7
		Ireland	71.7 (65.8-77.0)	63.3 (56.7-69.5)	15.1 (10.3-19.8)	22.9 (18.7-27.2)	15.7 (11.1-20.3)	3.1 (1.4-5.1)	1.1
		Italy	63.8 (57.1-70.1)	54.5 (47.5-61.4)	13.3 (8.9-17.8)	20.4 (16.4-24.5)	14.0 (9.9-18.2)	1.1 (-0.7-2.9)	14.7
		Latvia	74.6 (67.9-80.4)	66.6 (59.0-73.6)	15.7 (10.8-20.7)	23.9 (19.3-28.5)	16.2 (11.2-21.1)	3.9 (1.9-6.1)	1.5
		Lithuania	76.8 (70.7-82.0)	69.2 (62.1-75.6)	16.2 (11.2-21.3)	24.6 (20.0-29.1)	16.7 (11.8-21.6)	4.5 (2.5-6.7)	2.3
		Luxembourg	73.6 (67.7-78.7)	65.5 (58.8-71.5)	15.5 (10.7-20.5)	23.5 (19.1-27.9)	16.0 (11.3-20.7)	3.6 (1.8-5.7)	0.1
		Malta	63.8 (55.8-71.1)	54.5 (46.2-62.6)	13.2 (8.7-17.8)	20.4 (16.2-24.8)	13.9 (9.7-18.2)	1.0 (-1.1-3.2)	0.1
		Netherlands	74.7 (69.0-79.7)	66.7 (60.2-72.7)	15.7 (10.7-20.6)	23.9 (19.3-28.2)	16.3 (11.4-21.1)	3.9 (2.1-5.9)	5.0
		Norway	70.4 (64.0-76.1)	61.8 (54.7-68.4)	14.8 (10.1-19.5)	22.5 (18.2-26.9)	15.4 (10.8-19.8)	2.8 (1.0-4.8)	1.2
		Poland	68.5 (61.0-75.2)	59.6 (51.5-67.3)	14.3 (9.6-19.0)	21.9 (17.5-26.3)	15.0 (10.4-19.5)	2.3 (0.3-4.4)	18.2
		Portugal	62.5 (54.0-70.2)	53.1 (44.4-61.6)	12.9 (8.7-17.5)	20.0 (15.7-24.5)	13.6 (9.4-18.0)	0.7 (-1.6-3.0)	3.1
		Slovakia	68.9 (61.5-75.4)	60.1 (52.1-67.6)	14.4 (9.7-19.2)	22.0 (17.7-26.4)	15.0 (10.4-19.6)	2.4 (0.4-4.5)	2.6
		Slovenia	72.3 (65.4-78.3)	64.0 (56.2-71.0)	15.1 (10.3-20.0)	23.1 (18.7-27.6)	15.8 (11.1-20.4)	3.3 (1.3-5.5)	0.8

		Spain	77.1 (71.5-81.9)	69.6 (63.0-75.5)	16.3 (11.1-21.4)	24.6 (20.2-29.1)	16.8 (11.8-21.7)	4.6 (2.6-6.7)	14.1
		Sweden	78.5 (73.4-82.9)	71.3 (65.2-76.7)	16.6 (11.5-21.6)	25.1 (20.4-29.6)	17.2 (12.1-22.1)	4.9 (3.0-7.0)	2.9
		Switzerland	78.3 (72.2-83.3)	71.0 (63.8-77.2)	16.6 (11.4-21.6)	25.0 (20.3-29.6)	17.1 (12.1-21.9)	4.9 (2.8-7.1)	2.2
		United Kingdom	68.5 (62.2-74.2)	59.6 (52.8-66.2)	14.3 (9.7-18.9)	21.9 (17.8-26.1)	15.0 (10.5-19.4)	2.3 (0.6-4.1)	17.1
	Upper-middle								
		Armenia	76.7 (69.3-82.7)	69.1 (60.5-76.5)	16.2 (11.1-21.3)	24.5 (19.9-29.3)	16.7 (11.8-21.7)	4.4 (2.2-7.0)	1.4
		Belarus	86.3 (81.3-90.2)	81.1 (74.7-86.2)	18.5 (12.9-24.0)	27.6 (22.7-32.4)	18.8 (13.3-24.2)	7.0 (4.6-9.6)	9.0
		Bosnia and Herzegovina	77.2 (69.7-83.3)	69.7 (61.0-77.2)	16.3 (11.0-21.5)	24.7 (19.9-29.6)	16.8 (11.8-21.9)	4.6 (2.2-7.2)	1.9
		Bulgaria	64.4 (56.4-71.7)	55.2 (46.8-63.3)	13.3 (8.8-17.9)	20.6 (16.3-25.0)	14.1 (9.8-18.5)	1.2 (-1.0-3.4)	4.4
		Kazakhstan	73.9 (65.6-80.8)	65.8 (56.5-74.1)	15.6 (10.6-20.7)	23.6 (18.8-28.4)	16.1 (11.2-21.1)	3.7 (1.4-6.2)	9.0
		Romania	67.9 (60.3-74.7)	59.0 (50.8-66.7)	14.2 (9.5-19.0)	21.8 (17.4-26.2)	14.8 (10.3-19.4)	2.1 (0.1-4.3)	11.1
		Russian Federation	83.4 (79.3-87.6)	77.4 (72.3-82.8)	17.7 (12.3-23.0)	26.7 (21.9-31.4)	18.2 (12.8-23.2)	6.2 (4.1-8.5)	127.3
		Serbia	65.2 (56.5-73.0)	56.0 (46.9-64.8)	13.6 (9.0-18.3)	20.8 (16.5-25.4)	14.2 (9.8-18.6)	1.4 (-0.9-3.8)	4.5
		Turkey	78.3 (71.6-83.7)	71.0 (63.2-77.7)	16.6 (11.4-21.8)	25.0 (20.3-29.7)	17.1 (12.0-22.0)	4.9 (2.7-7.3)	21.1
	Lower-middle								
		Georgia	82.7 (76.9-87.4)	76.5 (69.4-82.5)	17.6 (12.3-22.9)	26.5 (21.7-31.2)	18.0 (12.7-23.2)	6.0 (3.8-8.5)	3.0
		Kyrgyzstan	89.1 (84.0-92.8)	84.7 (78.1-89.8)	19.1 (13.1-24.6)	28.5 (23.4-33.4)	19.4 (13.7-25.0)	7.8 (5.1-10.5)	2.4
		Republic of Moldova	87.9 (83.3-91.3)	83.2 (77.2-87.7)	18.9 (13.2-24.4)	28.1 (23.0-32.9)	19.2 (13.6-24.5)	7.5 (5.0-10.0)	3.4
		Ukraine	81.3 (75.1-86.3)	74.7 (67.2-81.1)	17.3 (11.9-22.7)	26.0 (21.2-30.8)	17.7 (12.5-23.0)	5.7 (3.5-8.2)	40.8
		Uzbekistan	86.7 (81.1-90.8)	81.6 (74.5-87.0)	18.6 (13.1-24.1)	27.7 (22.7-32.7)	18.9 (13.3-24.3)	7.1 (4.7-9.8)	10.5
	Low								
		Tajikistan	80.1 (72.4-86.0)	73.2 (64.1-80.7)	17.0 (11.7-22.3)	25.6 (20.7-30.6)	17.5 (12.2-22.8)	5.3 (3.0-8.0)	2.0
South-East Asia									
	Upper-middle								
		Maldives	74.2 (65.4-81.5)	66.2 (56.2-75.0)	15.6 (10.6-20.8)	23.7 (18.8-28.6)	16.2 (11.3-21.2)	3.8 (1.2-6.5)	0.1
		Thailand	78.2 (71.5-83.7)	70.9 (63.0-77.7)	16.5 (11.5-21.8)	25.0 (20.3-29.7)	17.1 (12.1-22.0)	4.8 (2.7-7.3)	30.4
	Lower-middle								
		Bangladesh	83.9 (77.5-90.3)	78.0 (70.1-86.4)	17.9 (12.4-23.5)	26.9 (21.8-31.9)	18.3 (12.8-23.7)	6.4 (3.8-9.2)	49.1

		Bhutan	82.3 (79.4-85.2)	76.0 (72.4-79.6)	17.5 (12.1-22.8)	26.3 (21.8-30.7)	17.9 (12.7-23.0)	6.0 (4.0-8.0)	0.2
		India	75.3 (63.7-84.2)	67.5 (54.4-78.4)	15.9 (10.4-21.4)	24.1 (18.6-29.7)	16.5 (11.2-22.0)	4.1 (0.8-7.4)	485.0
		Indonesia	76.5 (68.4-83.1)	68.9 (59.5-77.0)	16.2 (11.1-21.4)	24.4 (19.6-29.3)	16.7 (11.7-21.7)	4.4 (2.0-7.1)	101.8
		Myanmar	91.9 (88.7-94.2)	88.5 (84.2-91.7)	19.8 (13.9-25.6)	29.4 (24.3-34.2)	20.1 (14.2-25.6)	8.6 (5.9-11.2)	25.7
		Sri Lanka	79.8 (72.8-85.3)	72.9 (64.5-79.8)	16.9 (11.6-22.3)	25.5 (20.6-30.3)	17.4 (12.3-22.6)	5.3 (3.0-7.8)	8.8
		Timor-Leste	89.7 (85.0-93.1)	85.5 (79.4-90.2)	19.3 (13.5-24.7)	28.7 (23.6-33.6)	19.6 (13.7-25.3)	8.0 (5.4-10.6)	0.3
	Low								
		Nepal	88.0 (85.5-90.5)	83.3 (80.0-86.6)	18.9 (13.3-24.4)	28.1 (23.3-32.9)	19.2 (13.7-24.5)	7.5 (5.2-9.8)	11.0
Western Pacific									
	High								
		Australia	73.0 (66.0-78.9)	64.8 (56.9-71.8)	15.3 (10.3-20.5)	23.4 (18.9-28.0)	15.9 (11.1-20.6)	3.5 (1.5-5.7)	5.0
		Brunei Darussalam	78.8 (69.4-86.0)	71.6 (60.7-80.7)	16.7 (11.3-22.4)	25.2 (20.1-30.4)	17.2 (11.9-22.5)	5.0 (2.2-8.1)	0.1
		French Polynesia	86.5 (81.3-90.4)	81.3 (74.7-86.5)	18.5 (13.0-24.0)	27.7 (22.6-32.7)	18.9 (13.2-24.2)	7.1 (4.7-9.7)	0.1
		Japan	66.2 (49.1-79.9)	57.1 (39.6-73.0)	13.7 (8.3-19.5)	21.2 (14.6-28.1)	14.4 (9.1-20.2)	1.6 (-2.7-5.9)	37.1
		New Zealand	60.7 (53.9-67.2)	51.2 (44.3-58.2)	12.5 (8.5-16.8)	19.4 (15.6-23.4)	13.2 (9.3-17.3)	0.3 (-1.6-2.0)	0.8
		Palau	71.7 (62.2-79.6)	63.3 (52.8-72.6)	15.0 (10.1-20.0)	22.9 (18.1-27.9)	15.7 (10.8-20.5)	3.1 (0.6-5.8)	0.0[e]
		Republic of Korea	70.5 (54.4-82.7)	61.9 (44.8-76.5)	14.7 (9.1-20.8)	22.6 (16.3-29.1)	15.4 (9.9-21.2)	2.7 (-1.7-7.0)	13.9
		Singapore	65.7 (49.1-79.2)	56.6 (39.6-72.1)	13.6 (8.2-19.6)	21.0 (14.7-27.7)	14.3 (9.1-20.1)	1.5 (-3.0-5.9)	1.1
	Upper-middle								
		American Samoa	50.7 (39.2-62.0)	41.1 (30.5-52.6)	10.3 (6.3-14.9)	16.2 (11.6-21.1)	11.1 (7.1-15.3)	-2.3 (-5.7-0.8)	0.0[e]
		China	84.0 (78.6-88.3)	78.1 (71.4-83.7)	18.0 (12.5-23.3)	26.8 (21.9-31.7)	18.4 (13.1-23.5)	6.4 (4.1-8.9)	581.9
		Cook Islands	90.2 (86.4-93.1)	86.2 (81.2-90.2)	19.4 (13.6-24.9)	28.9 (23.8-33.7)	19.7 (14.0-25.1)	8.1 (5.6-10.7)	0.0[e]
		Fiji	89.2 (84.9-92.3)	84.9 (79.3-89.1)	19.1 (13.4-24.7)	28.5 (23.6-33.4)	19.5 (13.9-24.8)	7.8 (5.3-10.4)	0.5
		Malaysia	65.4 (56.4-73.4)	56.2 (46.8-65.2)	13.6 (9.1-18.4)	20.9 (16.5-25.6)	14.3 (9.8-18.9)	1.4 (-0.9-3.9)	7.4
		Marshall Islands	63.0 (53.3-71.7)	53.6 (43.7-63.3)	13.0 (8.5-17.8)	20.1 (15.6-24.9)	13.8 (9.4-18.3)	0.8 (-1.7-3.4)	0.0[e]
		Nauru	65.1 (54.7-74.2)	55.9 (45.1-66.2)	13.5 (8.8-18.5)	20.8 (16.1-25.8)	14.2 (9.6-19.0)	1.4 (-1.3-4.1)	0.0[e]
		Samoa	91.8 (88.5-94.2)	88.4 (84.0-91.7)	19.8 (14.0-25.5)	29.4 (24.2-34.3)	20.0 (14.2-25.6)	8.6 (5.9-11.2)	0.1
		Tonga	91.5 (88.1-94.1)	88.0 (83.4-91.6)	19.7 (14.0-25.3)	29.3 (24.3-34.2)	19.9 (14.1-25.4)	8.5 (5.9-11.1)	0.0

		Tuvalu	82.5 (76.3-87.3)	76.2 (68.6-82.4)	17.6 (12.3-22.9)	26.3 (21.5-31.3)	18.0 (12.6-23.2)	6.0 (3.7-8.6)	0.0[e]
	Lower-middle								
		Cambodia	90.2 (85.6-93.4)	86.2 (80.2-90.6)	19.4 (13.5-25.1)	28.8 (23.8-33.8)	19.7 (14.0-25.0)	8.1 (5.4-10.8)	4.9
		Federated States of Micronesia	63.4 (54.0-71.8)	54.1 (44.4-63.4)	13.1 (8.7-17.8)	20.2 (15.7-24.9)	13.8 (9.4-18.4)	0.9 (-1.5-3.4)	0.0
		Kiribati	65.5 (56.3-73.7)	56.3 (46.7-65.6)	13.6 (9.0-18.4)	20.9 (16.4-25.7)	14.3 (9.7-18.8)	1.5 (-1.0-4.0)	0.0
		Lao People's Democratic Republic	88.3 (86.6-90.0)	83.7 (81.5-86.0)	19.0 (13.4-24.5)	28.2 (23.3-32.8)	19.3 (13.8-24.5)	7.6 (5.3-9.9)	2.2
		Mongolia	82.2 (77.1-86.4)	75.8 (69.6-81.2)	17.5 (12.1-22.8)	26.2 (21.4-30.8)	17.9 (12.7-23.0)	5.9 (3.8-8.2)	1.3
		Niue	92.2 (89.0-94.6)	88.9 (84.6-92.3)	20.0 (14.1-25.6)	29.4 (24.3-34.4)	20.1 (14.2-25.7)	8.7 (6.1-11.3)	0.0[e]
		Papua New Guinea	88.6 (83.0-92.5)	84.1 (76.8-89.3)	19.0 (13.2-24.7)	28.3 (23.3-33.3)	19.3 (13.7-24.7)	7.7 (5.0-10.4)	3.2
		Philippines	69.9 (62.1-76.6)	61.2 (52.7-69.0)	14.6 (10.0-19.4)	22.3 (17.9-26.9)	15.2 (10.6-19.9)	2.6 (0.5-4.9)	34.6
		Solomon Islands	86.7 (81.8-90.5)	81.6 (75.3-86.6)	18.6 (13.1-24.0)	27.7 (22.8-32.5)	18.9 (13.2-24.2)	7.1 (4.8-9.7)	0.2
		Tokelau	95.5 (93.3-97.0)	93.5 (90.4-95.6)	20.7 (14.6-26.3)	30.5 (25.3-35.5)	20.8 (14.8-26.6)	9.6 (6.7-12.3)	0.0[e]
		Vanuatu	92.8 (89.6-95.1)	89.8 (85.4-93.0)	20.1 (14.2-25.8)	29.7 (24.5-34.6)	20.3 (14.4-25.8)	8.8 (6.1-11.5)	0.1
		Viet Nam	80.1 (73.9-85.2)	73.2 (65.8-79.6)	17.0 (11.7-22.2)	25.6 (20.9-30.3)	17.5 (12.3-22.5)	5.3 (3.1-7.7)	28.8

Number of deaths averted for males and females may not add up to the estimate for both sexes. This is because the distributions of deaths across males and females are not even, and differences are magnified when multiplied by a derivative of activity prevalence which also varies considerably by sex.

a: The main estimates calculated using Formula 3 (see Box 1), the adjusted relative risk, and 0% activity counterfactual.

b: The unadjusted estimates calculated using Formula 2 (see Box 1), the unadjusted relative risk, and 0% activity counterfactual.

c: The partially adjusted estimates calculated using Formula 2 (see Box 1), the adjusted relative risk, and 0% activity counterfactual.

d: The adjusted estimates calculated using Formula 5 (see Appendix 10, pp23-25), the adjusted relative risk, and 54.3% activity counterfactual.

e: Deaths averted based on an imputed estimate of total deaths between the ages of 40-79 years. This was derived using the median ratio of deaths to total population for countries in the same region/income categories (Appendix 9, p22).
